# Reactive oxygen species mediated apoptotic death of colon cancer cells: therapeutic potential of plant derived alkaloids

**DOI:** 10.3389/fendo.2023.1201198

**Published:** 2023-07-25

**Authors:** Vinod K. Nelson, Mohana Vamsi Nuli, Juturu Mastanaiah, Mohamed Saleem T. S., Geetha Birudala, Yahya F. Jamous, Omar Alshargi, Kranthi Kumar Kotha, Hari Hara Sudhan, Ravishankar Ram Mani, Alagusundaram Muthumanickam, Divya Niranjan, Nem Kumar Jain, Ankur Agrawal, Arvind Singh Jadon, Vinyas Mayasa, Niraj Kumar Jha, Adriana Kolesarova, Petr Slama, Shubhadeep Roychoudhury

**Affiliations:** ^1^ Raghavendra Institute of Pharmaceutical Education and Research, Anantapur, India; ^2^ Department of Pharmacology, Balaji College of Pharmacy, Anantapur, India; ^3^ College of Pharmacy, Riyadh ELM University, Riyadh, Saudi Arabia; ^4^ Faculty of Pharmacy, Dr. M.G.R. Educational and Research Institute, Chennai, India; ^5^ Vaccines and Bioprocessing Centre, King Abdulaziz City for Science and Technology (KACST), Riyadh, Saudi Arabia; ^6^ Department of Pharmaceutics, College of Pharmaceutical Sciences, Dayananda Sagar University, Bengaluru, India; ^7^ Faculty of Pharmaceutical Sciences, UCSI University, Kuala Lumpur, Malaysia; ^8^ School of Pharmacy, ITM University, Gwalior, India; ^9^ Amity Institute of Pharmacy, Amity University, Gwalior, India; ^10^ GITAM School of Pharmacy, GITAM University Hyderabad Campus, Rudraram, India; ^11^ Department of Biotechnology, School of Engineering and Technology, Sharda University, Greater Noida, India; ^12^ Department of Biotechnology, School of Applied & Life Sciences (SALS), Uttaranchal University, Dehradun, India; ^13^ School of Bioengineering & Biosciences, Lovely Professional University, Phagwara, India; ^14^ Department of Biotechnology Engineering and Food Technology, Chandigarh University, Mohali, India; ^15^ Faculty of Biotechnology and Food Sciences, Slovak University of Agriculture in Nitra, Nitra, Slovakia; ^16^ Laboratory of Animal Immunology and Biotechnology, Department of Animal Morphology, Physiology and Genetics, Faculty of AgriSciences, Mendel University in Brno, Brno, Czechia; ^17^ Department of Life Science and Bioinformatics, Assam University, Silchar, India

**Keywords:** oxidative stress, mutation, IGF-1, colon cancer progression, alkaloids, HIF-1α, IGFBP-3, apoptosis

## Abstract

Colorectal cancer (CRC) is one of the most deaths causing diseases worldwide. Several risk factors including hormones like insulin and insulin like growth factors (e.g., IGF-1) have been considered responsible for growth and progression of colon cancer. Though there is a huge advancement in the available screening as well as treatment techniques for CRC. There is no significant decrease in the mortality of cancer patients. Moreover, the current treatment approaches for CRC are associated with serious challenges like drug resistance and cancer re-growth. Given the severity of the disease, there is an urgent need for novel therapeutic agents with ideal characteristics. Several pieces of evidence suggested that natural products, specifically medicinal plants, and derived phytochemicals may serve as potential sources for novel drug discovery for various diseases including cancer. On the other hand, cancer cells like colon cancer require a high basal level of reactive oxygen species (ROS) to maintain its own cellular functions. However, excess production of intracellular ROS leads to cancer cell death *via* disturbing cellular redox homeostasis. Therefore, medicinal plants and derived phytocompounds that can enhance the intracellular ROS and induce apoptotic cell death in cancer cells *via* modulating various molecular targets including IGF-1 could be potential therapeutic agents. Alkaloids form a major class of such phytoconstituents that can play a key role in cancer prevention. Moreover, several preclinical and clinical studies have also evidenced that these compounds show potent anti-colon cancer effects and exhibit negligible toxicity towards the normal cells. Hence, the present evidence-based study aimed to provide an update on various alkaloids that have been reported to induce ROS-mediated apoptosis in colon cancer cells *via* targeting various cellular components including hormones and growth factors, which play a role in metastasis, angiogenesis, proliferation, and invasion. This study also provides an individual account on each such alkaloid that underwent clinical trials either alone or in combination with other clinical drugs. In addition, various classes of phytochemicals that induce ROS-mediated cell death in different kinds of cancers including colon cancer are discussed.

## 1 Introduction

Globally, cancer is one of the highest mortality-causing diseases. Though there is a drastic improvement in the current treatment schedules followed for various cancers, there is no marked decrease of death rate (1, 2). Among the various dreadful cancers, colorectal cancer (CRC) stood in third and second globally regarding prevalence and death rates, respectively (3, 4). Moreover, a rapid rise in the cases and death rates of CRC has been predicted soon (5). By 2035, a global rise of 2.5 million cases has been estimated (6). However, the CRC incidence varies from country to country, and the maximum number of cases have been reported from the developed world (7). Colorectal tumors were more influenced by gender and sex, where male populations have been affected more significantly than the females (8). In addition, the other risk factors, such as age, consumption of alcohol, diet with high fat and low fruits, vegetables, and maintaining low physical activities, influences the level of progression of the disease (5). Growing evidence also suggests that mutations in the growth factors such as insulin-like growth factor 1 (IGF-1) also shows a massive impact on the development of colon cancer *via* activating rat sarcoma/mitogen-activated protein kinase/phosphatidylinositol-3 kinase/protein kinase B (RAS/MAPK/PI3K/AKT) pathways (4, 9). On the other hand, hormonal alterations also show significant role in production of ROS, which plays an important role in CRC development. Various studies have revealed that hormones like thyroid hormones, corticosteroids and catecholamines are also involved in the generation of ROS by modulating various signaling molecules and pathways like NADPH oxidase (NOX) pathway, mitochondrial electron transport chain (ETC), nitric oxide (NO), and nuclear factor erythroid 2-related factor 2 (Nrf2), apart from their normal functions (10, 11). In addition, some diseases like inflammatory bowel disorder (IBD) also serves as significant risk factors for colon cancer (12, 13).

Recently, there has been a huge advancement in the screening and treatment techniques of CRC. Among the various available treatments, surgery is the preferred treatment option for colon cancer patients (14). However, these treatments cannot effectively reach low-income patients and can only extend the survival time of CRC patients (4, 14). Furthermore, many effective chemotherapeutic drug candidates such as 5-fluorouracil, oxaliplatin, capecitabine, and irinotecan that are clinically recommended for colon cancer treatment either individually or in combination exhibit severe toxicity to normal cells (15, 16). Therefore, there is an urgent need for novel drug treatment with ideal characteristics to treat colorectal carcinoma. From ancient days natural products, specifically medicinal plants and herbal products, have played a significant role in identifying novel treatments against various diseases (17–24). Moreover, several studies have reported plant-derived components as safe to use, and they exhibited minimal toxicity towards non-cancerous cells (2, 25). Hence, these phytocompounds have also been suggested for use together with other clinically recommended chemotherapy drugs to reduce toxicity.

In general, cancer cells require a high level of reactive oxygen species (ROS) compared to normal cells due to elevated metabolic rate and mitochondrial dysfunction, and this makes the cancer cells more vulnerable to oxidative stress. Hence, an increased level of ROS than required can trigger the death-inducing signals in cancer cells *via* irreversible damage to various biomolecules. In contrast to cancer cells, normal cells develop potent antioxidant proteins that can nullify the toxic effects of ROS (26). Therefore, minimal elevation of intracellular ROS than the threshold limit *via* intervention with external agents can make the cancer cells more sensitive to oxidative stress than the normal cells (27–29). There are several phytocompounds, such as curcumin, capsaicin, sulforaphane, alpha-lipoic acid, and piperine belonging to the class of alkaloids, flavonoids, terpenoids, and phenolic compounds that are known to initiate ROS-mediated apoptotic cell death. Among the various chemical classes, alkaloids are the most divergent and vigorously investigated class of phytochemicals in different diseases including cancer. Various alkaloids like vincristine, vinblastine, evodiamine, sanguinarine, matrine, tetrandrine, camptothecin, and berberine have already proved their anti-cancer potentials *via* targeting different kinds of essential signaling molecules such as MAPK, extracellular signal-related kinases 1/2 (ERK1/2), tumor suppressor protein (p53), p38 mitogen-activated protein kinase, c-Jun N-terminal kinase (JNK), and PI3K/Akt. Hence, forced activation of ROS in the cancer cells through small molecules like alkaloids derived from medicinal plants and altering the signaling molecules that initiate the death process can improve the cancer condition. Moreover, several kinds of alkaloids like berberine, camptothecin and epigallocatechin as such or as derivatives have been under clinical trials at different stages (30; 31). Some of them have till now exhibited a good safety profile as compared to other recommended drugs (32, 33). Hence, this evidence-based study focused on the alkaloids and their ROS-mediated apoptotic signaling pathways. In addition, other classes of phytocompounds that induce ROS-mediated apoptosis and have been used in clinical trials either individually or in combination with other clinical drugs have also been looked at. Several studies have revealed that the synergetic or combination effect of medicinal plants or phytocompounds provides a novel therapeutic tool. Moreover, combinations of herbal compounds with existing clinical drugs have also been reported to increase the specificity and minimize the toxic effects of existing chemotherapy drugs. In this way, the current review acts as a standalone reference to all the classes of herbal compounds that involve ROS-mediated apoptotic cell death.

## 2 Methodology

The most relevant literature to conduct the study was extracted from electronic databases SCOPUS, and PubMed. The key words and the phrases used for collecting the highly relevant articles included colorectal cancer, alkaloids, ROS-mediated apoptosis, medicinal plants, phytochemicals, current therapies, side effects, hormones and growth factors, clinical trials, and related terms. Later, the findings from the selected studies were summarized by focusing on the alkaloids that induce ROS mediated apoptosis in colon cancer. The articles not published in English language were excluded.

## 3 ROS: sources and role in cancer development

**Figure 1:**
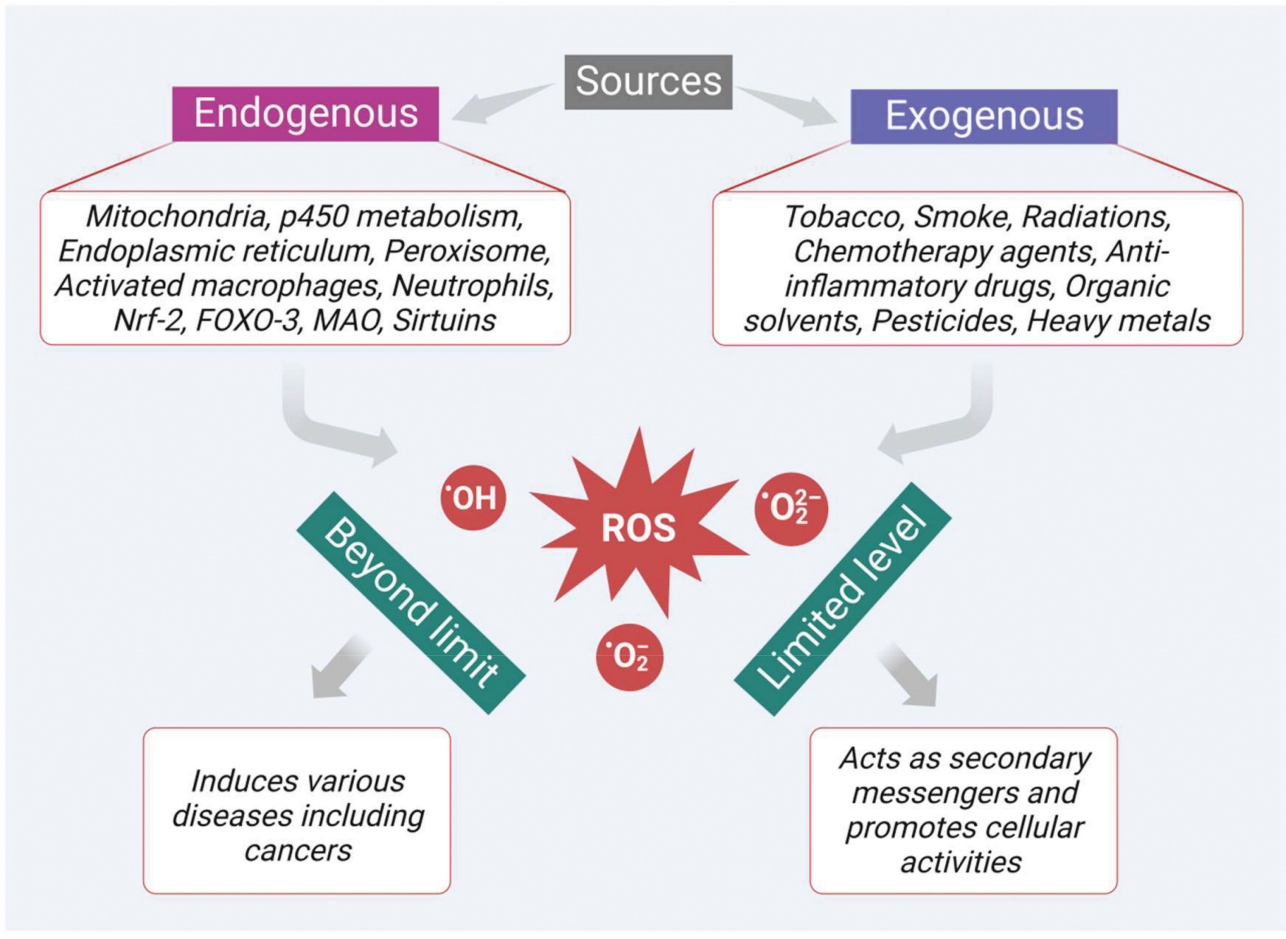
Sources of ROS generation which play dual role as secondary messengers that promote cellular activities at limited levels and as inducers of various diseases including cancer at excessive levels.

ROS are highly active and most unstable oxygen containing metabolic products, generally produced by various endogenous and exogenous sources. Among the multiple endogenous sources, mitochondria-based metabolic pathways, p450 metabolism, endoplasmic reticulum, peroxisome, activated macrophages, and neutrophils mainly contribute to ROS generation (**Figure 1**). Besides, there are other resources such as monoamine oxidase, sirtuins, forkhead box O3 (FOXO3), alpha-ketoglutarate dehydrogenase (α-KGDH), nuclear factor erythroid 2-related factor 2 (Nrf2) and mitochondrial p66^shc^ (proapoptotic protein) which also contribute to ROS generation (34). In addition, the innate immune response shown by the host against the pathogens also generates ROS as a defense mechanism. However, persistent or chronic inflammation leads to excessive production of ROS and develops various kinds of diseases including colon cancer. In a recent study, Sahoo and his group revealed that lipopolysaccharides (LPS)-induced chronic inflammation in the intestine may also lead to cancer in the gut. They observed that LPS treatment can enhance the levels of pro-cancer genes and help in cell proliferation in the colonoids obtained from the dogs suffering from IBD. Therefore, the persistent or chronic inflammatory diseases like IBD also play a part in the development of colon cancer though oxidative stress (13, 35).

In addition, there are many exogenous sources like tobacco smoking, radiation, chemotherapeutic agents, anti-inflammatory drugs, chemicals containing quinones, organic solvents, pesticides, and other heavy metals (like cadmium, chromium, and arsenic) that also produce ROS intermediates by interacting with other molecules. In general, ROS like hydroxyl radical (^•^OH), superoxide radical (O_2_^•–^), and hydrogen peroxide (H_2_O_2_) at a limited level are crucial to cellular activities (**Figure 1**). They act as secondary messengers, promoting various essential cellular activities such as signal transduction, immune responses, and gene transcription. However, when the levels of ROS increase beyond the limit, it leads to oxidative stress and damages various vital biomolecules such as lipids, proteins, deoxyribonucleic acids (DNA), and ribonucleic acids (RNA). Moreover, ROS also initiate the development of various diseases, such as cardiovascular diseases, cataracts, neurodegenerative diseases, inflammation, diabetes mellitus, and cancer (**Figure 1**). Furthermore, ROS plays an important role in the initiation, development, and progression of cancer. Besides, the cancer cells themselves require a high level of ROS in contrast to the normal cells due to the high metabolic rate and increased energy demand. These increased levels of ROS *via* multiple sources interact with various essential signaling molecules thereby activating various growth factors like epidermal growth factor (EGF), and IGF-1, and inducing mutation in their receptors.

**Figure 2:**
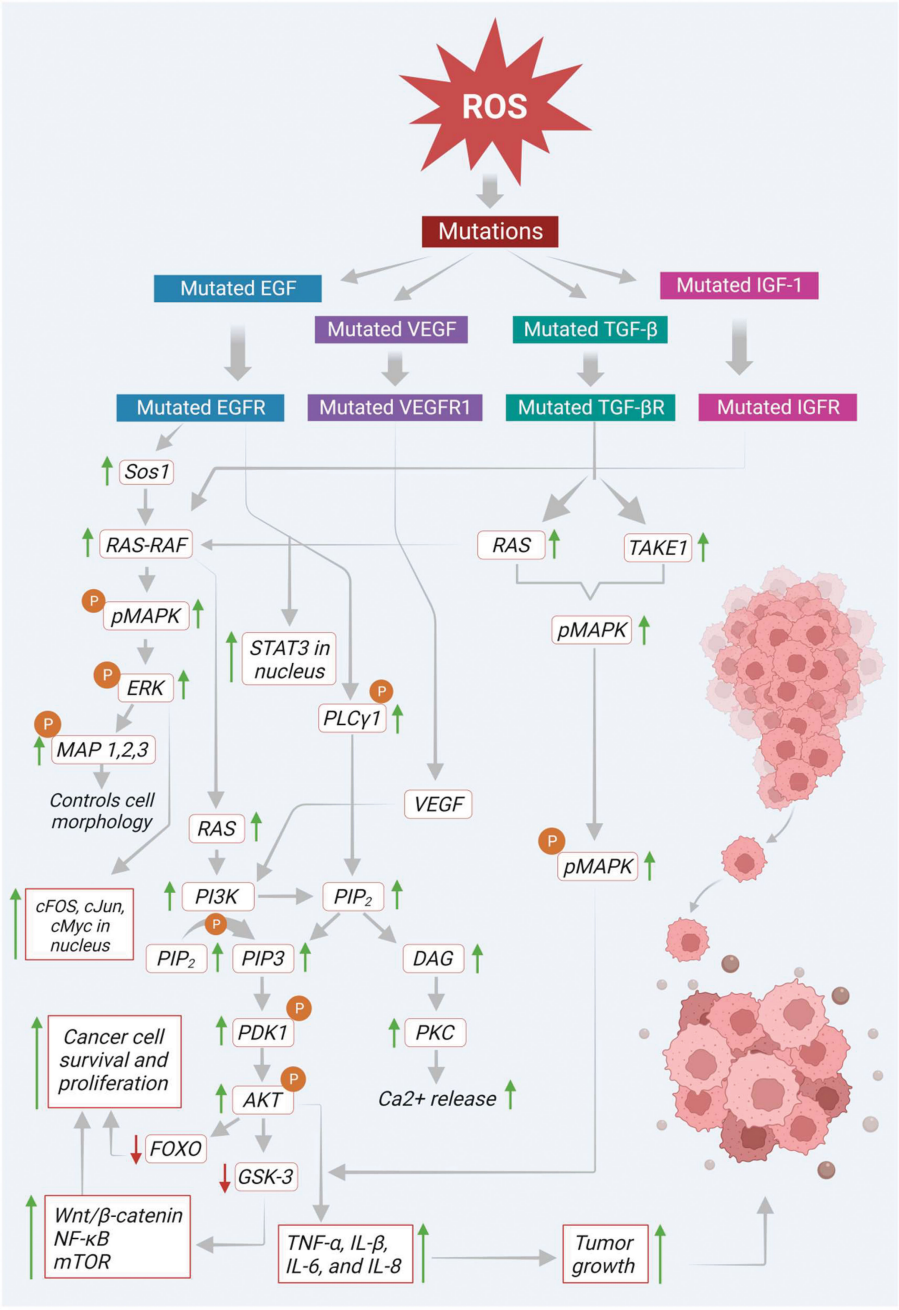
Basal levels of ROS initiate mutation in various transmembrane receptors thereby modulating multiple signaling molecules/pathways that involve tumor growth and progression.

Mutated EGF receptor (EGFR) elevates the expression of its downstream signaling molecules such as rat sarcoma - rapidly accelerated fibrosarcoma (RAS-RAF), MAPK, AKT, PI3K and mammalian target of rapamycin (mTOR). In the initial stage of the pathway, the EGFR initiates son of sevenless homolog 1 (Sos 1) gene-mediated activation of RAS-RAF molecules. Then the activated RAS-RAF initiates the phosphorylation of MAPK, which later activates ERK1 and ERK2. The activated ERK immediately phosphorylates the various components of cytoskeleton like MAP 1, 2, and 4 in the cytoplasm, which controls the cell morphology and other functions. In addition, the activated ERK also shifts to the nucleus and helps in the transcription of various signaling molecules like proto-oncogene fos proto-oncogene (c-Fos), transcription factor Jun (c-Jun), cellular myelocytomatosis oncogene (c-Myc), erythroblast transformation specific (ETS) domain-containing protein ETS domain transcription factor ELK1 (Elk-1) and cyclic adenosine monophosphate (cAMP) dependent transcription factor ATF2 (activating transcription factor 2) (36). Mitogen-activated extracellular signal-regulated kinase (MEK) and ERK signaling molecules, proto-oncogene B-Raf (BRAF) (a downstream proto-oncogene of the RAF family) play a significant role in the activation of RAS. On the other hand, RAS also triggers the activation of PI3K signaling molecules, which promotes the conversion of PI-3, phosphatidylinositol 4,5-bisphosphate (PIP_2_) to phosphatidylinositol (3,4,5)-trisphosphate (PIP_3_). This PIP_3_ activates protein kinase B/Akt *via* phosphoinositide-dependent kinase 1 (PDK1) dependent phosphorylation. Then the activated Akt inactivates the transcription activity of FOXO and its interlinked downstream signals *via* phosphorylation and finally promotes the survival, proliferation, and growth of cancer cells (37). Besides Akt activation, it also inhibits glycogen synthase kinase-3 (GSK-3) *via* phosphorylation. This inactivation of GSK-3 protein leads to the activation of various signaling molecules such as (wingless gene) WNT/β-catenin, nuclear factor kappa B (NF-κB), mTOR and its respective downstream signals (**Figure 2**).

In addition, activated EGFR directly phosphorylates and activates phosphoinositide-specific phospholipase C gamma 1 (PLC-γ1). This PLC-γ1 facilitates the generation of PIP_3_ and diacylglycerol (DAG) from PIP_2_. DAG further triggers PKC activation and enhances intracellular calcium (Ca^+2^) release, ultimately leading to carcinogenesis (38). Moreover, EGFR also promotes transcriptional activation of signal transducer and activator of transcription 3 (STAT3) in the nucleus *via* direct interaction and enhances its biological functions such as tumor growth, differentiation, and apoptosis (**Figure 2**). On the other hand, angiogenesis is another critical factor that plays a prominent role in the colon cancer progression.

Besides, ROS also initiate the activation of vascular endothelial growth factor (VEGF) *via* upregulation of the activity of hypoxia-inducible factor 1-alpha (HIF-1α). This activated VEGF binds to its respective receptor to further activate its downstream signaling molecules (39). There are three types of VEGF receptors such as VEGF receptors 1, 2, and 3 (VEGFR-1, -2, and -3). Moreover, they are all different in their activities and sites of action (40). Among the various VEGFR receptors, VEGFR-1 is found explicitly in various cancers, epithelial and inflammatory cells and promotes biological functions *via* interacting with VEGF. This receptor predominately helps the cancer cells to migrate during angiogenesis (41). Furthermore, it also shows a significant contribution to inflammatory disease conditions, including various cancers, through the activation of downstream signals such as PI3K/Akt/MAPK/ERK, resulting in the release of various inflammatory cytokines, including tumor necrosis factor alpha (TNF-α), and interleukins (IL-1β, IL-6, and IL-8). As shown in **Figure 2**, these cytokines finally promote tumor growth in respective tissues (42).

In addition, ROS also induce transforming growth factor-beta (TGF-β), which plays a vital role in various fibrotic diseases. Growing evidence suggests that TGF-β also shows impact on tumor growth and progression, which is mediated by ROS. In cancer cells, ROS initiated TGF-β was reported to induce the MAPK signaling pathway *via* activating Ras and TGF-β-activated kinase 1 (TAK1) (34). The activated MAPK further triggers its downstream signaling molecules like ERK1 and ERK2. Finally, this MAPK and TGF-β together with other cytokine molecules, recommend epithelial-mesenchymal transition (EMT), where the tumor epithelial cells adopt mesenchymal-like characteristics that can help in tumor progression and metastasis (**Figure 2**).

Furthermore, ROS also induce IGFs that play a pivotal role in cancer survival and progression. Several studies revealed that ROS-mediated IGF-I activation also activates its corresponding receptor IGF-1 *via* phosphorylation. As shown in **Figure 2**, this activated IGF-1 turns on its downstream signaling, such as MAPK, Akt, mTOR and NF-κB *via* induction of Ras-Raf proto-oncogenes (9). The pathways mentioned above are involved in ROS-mediated signaling in cancer development. The subsequent sections highlight growth factors and corresponding signaling in cancer progression.

## 4 ROS mediated apoptotic signaling

ROS are short-lived metabolic by-products that generally possess an unpaired electron in their respective outermost shells. Because of the unpaired electron, the ROS are highly reactive and show both dangerous and beneficial effects (43, 44). In fact, at the level or below the threshold limit, ROS assist in the development of various cancers *via* initiation of mutations in various signaling molecules like p53 as well as upregulation of oncogenes like Ras and c-Myc. However, cancer cells initiate apoptotic cell death mechanisms due to ROS accumulation beyond the threshold limit (45). Normally, ROS-associated apoptotic cell death can be triggered *via* activation of intrinsic or mitochondrial signaling and upregulation of extrinsic or death receptor signaling.

**Figure 3:**
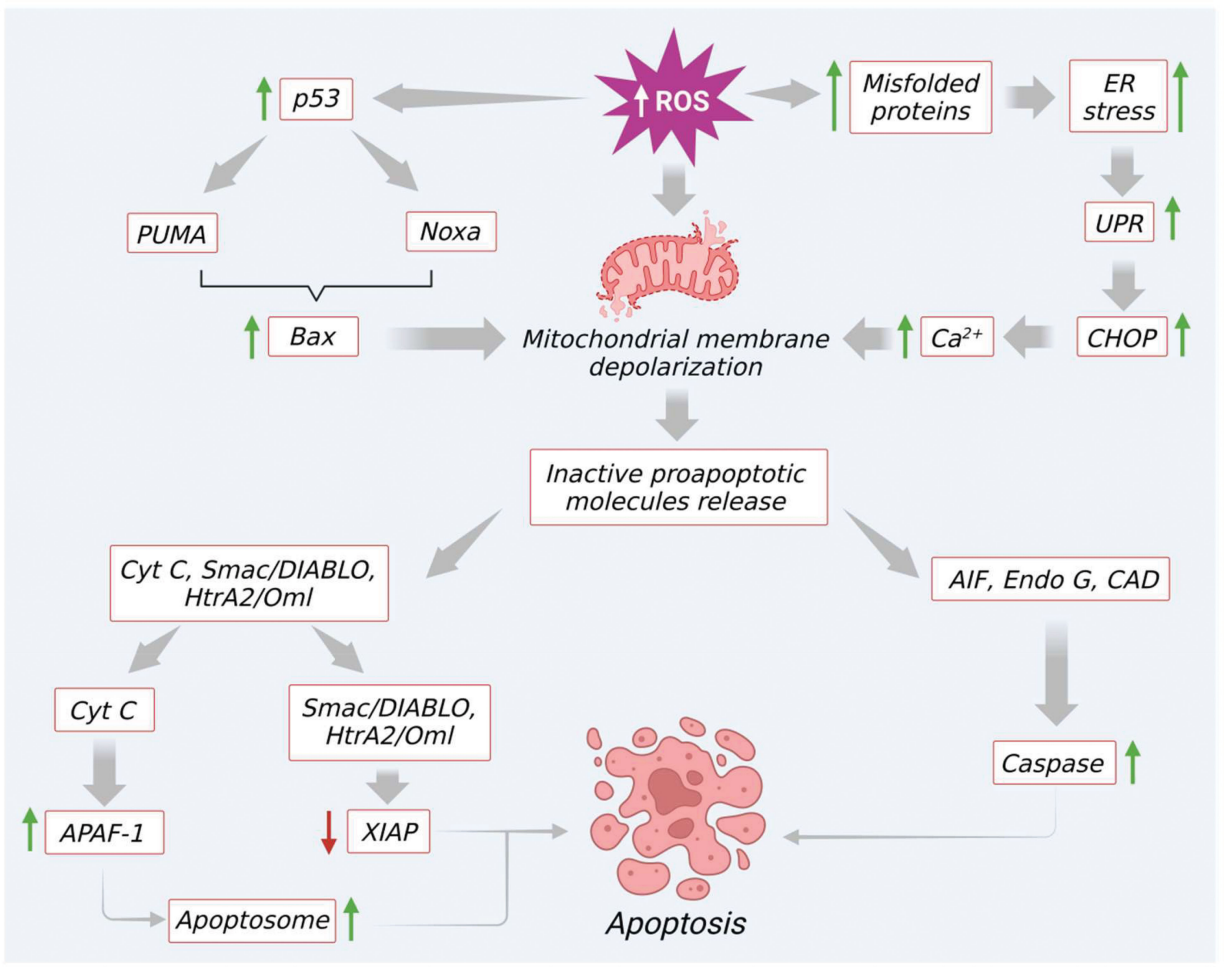
Excessive ROS generation induce mitochondrial mediated apoptosis through modulation of various signaling molecules.

In the intrinsic pathway, excessive ROS production (e.g., by H_2_O_2_) modulates mitochondrial permeability transition (MPT) pore and mitochondrial membrane depolarization. As a result, two very essential groups that consist of inactive pro-apoptotic molecules are released in the cytoplasm. One such group contains signaling molecules like cytochrome c, second mitochondria-derived activator of caspase (Smac)/DIABLO (a direct IAP binding protein with low PI), and serine protease Htr (with high-temperature requirements) A2 (Omi) (nuclear-encoded mitochondrial serine protease). Significantly, all these molecules are involved in caspase-dependent apoptotic pathways (43). To be specific, cytochrome c facilitates the proteolytic maturation of both caspases-3 and 9 *via* allosteric activation of apoptotic peptidase activating factor 1 (APAF-1). This activation finally leads to the formation of apoptosomes (46, 47). In a similar manner, as shown in **Figure 3**, other pro-apoptotic proteins like Smac/DIABLO as well as HtrA2/Omi also initiate the apoptotic process *via* inhibition of IAP like XIAP (47, 48). The second group released from the mitochondrial membrane comprises signaling molecules like apoptosis-inducing factor (AIF), endonuclease G, and caspase-activated DNAse (CAD). After immediate release AIF, endonuclease G, and CAD proteins slide inside the nucleus and initiate caspase-dependent apoptotic features in the cells like DNA fragmentation as well as chromatin condensation (47, 49). Besides, the Bcl-2 family proteins also control the release of various apoptotic signaling molecules including cytochrome c. In the Bcl-2 family, proteins such as Bax, Bak, Bid, Bad, Bim, Bik, and Blk facilitate apoptosis. The other remaining proteins like B-cell leukemia/lymphoma proteins (Bcl-2, Bcl-x, Bcl-XL, Bcl-XS, Bcl-w) and BAG (Bcl-2–associated athanogene), favor inhibition of apoptosis signaling (50, 51). Interestingly, these Bcl-2 family proteins can be significantly tuned by tumor suppressor protein p53. Several studies revealed that p53 directly interacts with Bcl-2 family proteins and helps in mitochondrial membrane depolarization and increases the release of apoptotic signals. To be specific, p53 plays a role in promoting p53 upregulated modulator of apoptosis (Puma) and Bcl2 homology domain 3 (BH3) (Noxa), the two essential proteins of the Bcl2 family (**Figure 3**). A previous report reveals that Puma, in particular, enhances Bcl-2-associated X protein (BAX) and Noxa supports p53 mediated apoptotic signaling (3, 52). Additionally, excessive ROS upregulation influences apoptosis through the elevation of Ca^2+^ levels, which increases mitochondrial permeability (52).

Furthermore, an elevated level of ROS or prolonged oxidative stress induces endoplasmic reticulum (ER) stress *via* increasing protein misfolding. This intrinsic pathway promotes the unfolded protein response and activates C/EBP homologous protein (CHOP) by triggering the induction of IRE1α, ASK, and p38 MAPK proteins. This finally leads to apoptosis by upregulation of Bcl-2 apoptotic protein. At the same time, excess ROS also prompts the discharge of Ca^2+^ from the ER lumen. Furthermore, due to the proximity of mitochondria to the ER, a maximum amount of Ca^2+^ is absorbed into mitochondria. This condition leads to Ca^2+^ overload in mitochondria and initiates the opening of MPTs, and facilitates the release of molecules like ATP and cytochrome c. These molecules further enhance ROS production and apoptotic signaling, as shown in **Figure 3**.

On the other hand, ROS also induces apoptosis *via* the regulation of signaling molecules involved in the extrinsic pathway. In this pathway, several death receptors like death receptor 1 (DR1) or tumor necrosis factor receptor 1 (TNF-R1), death receptor 2 (DR2) or type-II transmembrane protein (Fas), death receptor 3 (DR3) or TNF receptor family (TRAMP), death receptor 4 (DR4) or TNF-related apoptosis-inducing ligand-receptor-1 (TRAIL-R1), death receptor 5 (DR5) or TNF-related apoptosis-inducing ligand-receptor-1(TRAIL-R2) and death receptor 6 (DR6) or TNF receptor superfamily member 21(TNFRSF21), that are derived from the TNF-R superfamily, have been found to be involved prominently in the apoptosis process. Generally, the extrinsic apoptotic pathway gets triggered *via* induction of transmembrane death receptors through interacting with various receptor-specific ligands such as Fas ligand (FasL), TNF-α, Apo3 ligand (Apo3L), and Apo2 ligand (Apo2L) (53). However, ligands like FasL, TNF-α, and Apo are mainly produced by activated macrophages and interact with specific death receptors, thereby initiating the apoptosis process in the cancer cells (54, 55). In the first step, the FasL and TNF-α interact with transmembrane proteins Fas or DR2 and TNFR1 or DR1 that consist of respective death domains. Both death receptors initiate apoptosis signaling in different manners.

**Figure 4:**
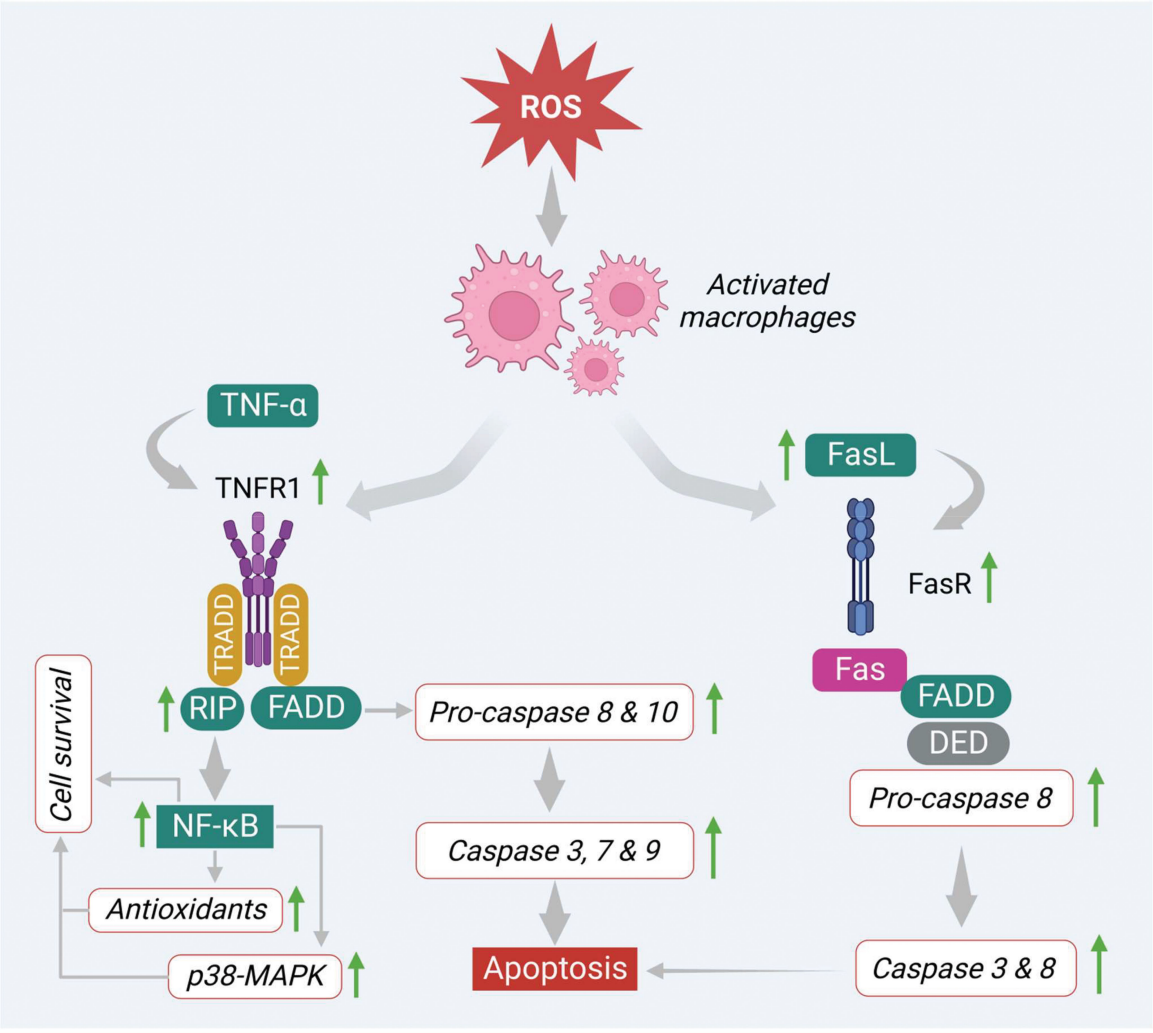
ROS-mediated extrinsic apoptosis *via* alteration of numerous intermediate signals.

In the TNF-α/TNFR1 pathway, the immediate interaction of TNF-α ligand to its receptor initiates the activation of TNFR1 *via* trimerization (**Figure 4**). This step engages other corresponding adapter molecules like TNFR type 1-associated DEATH domain protein (TRADD) and forms a complex in the cytoplasm. This complex activates and attracts a few more partners or signaling molecules, which decides the fate of the pathway (either cell survival or cell death). In this process, the TRADD complex attracts and activates other signaling molecules like tumor necrosis factor receptor 2 (TRAF2) and receptor-interacting protein kinases (RIP). This, in turn, triggers NFκB and MAPK-mediated cell survival process by increasing the levels of antioxidants as well as anti-apoptotic proteins such as Bcl-XL, X-linked inhibitor of apoptosis protein (XIAP), and inhibitors of apoptosis proteins (cIAP 1, 2) (53, 56) (**Figure 4**). On the other hand, the TRADD complex also stimulates apoptosis *via* recruiting FAS-associated death domain (FADD) protein. This complex promotes procaspases 8 and 10, which regulate the expression of caspases 3, 6 and 7 as shown in **Figure 4**.

Similarly, FasL activates apoptosis by binding to its corresponding receptor DR2 or Fas. This step activates the DR2 or Fas receptor *via* trimerization and recruits the other adaptor molecules like FADD. These linkages further recruit pro-caspase-8 through DED. This finally promotes the generation of DISC and triggers the downstream caspases such as caspases 3 and 6 and initiates the apoptotic signals in the cancer cells by inducing apoptotic features like nuclear condensation, DNA damage, and membrane blebbing (**Figure 4**).

**Figure 5:**
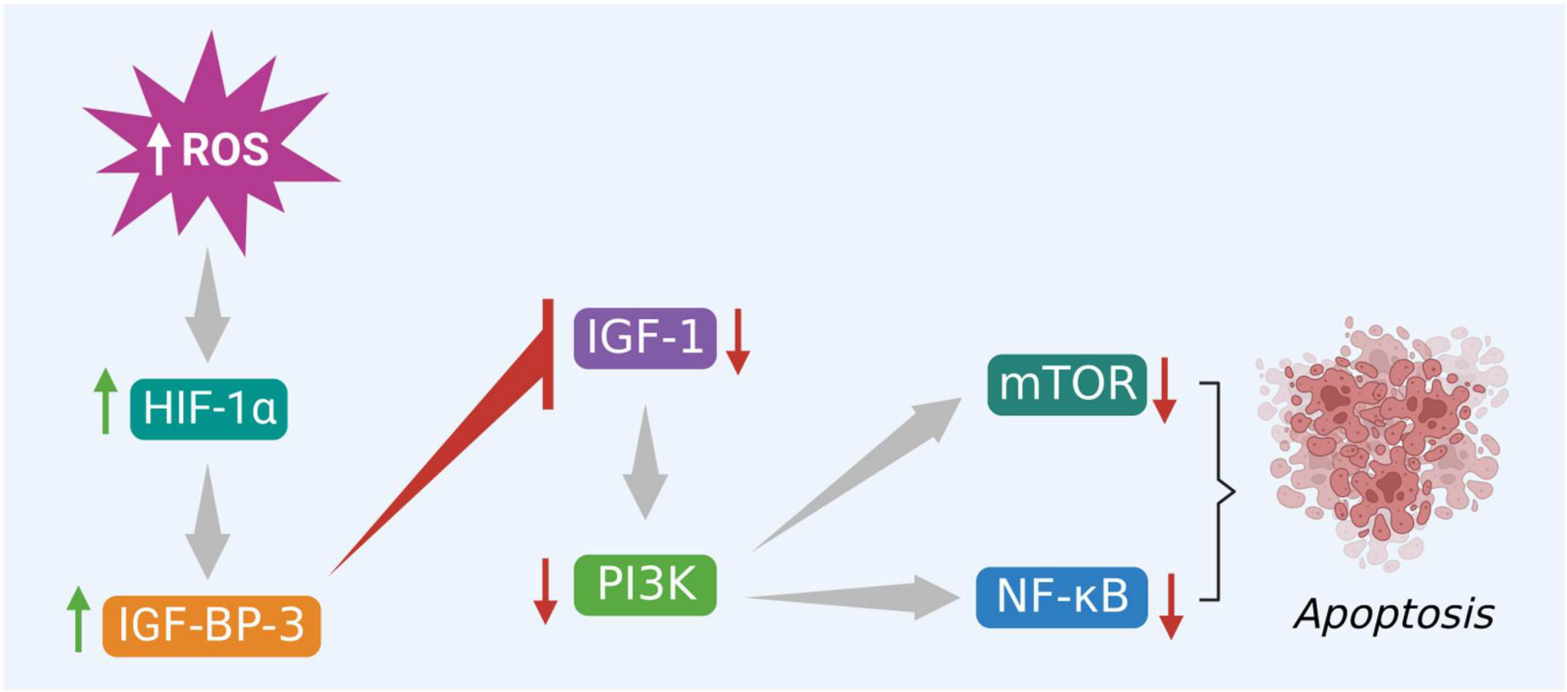
Excessive ROS levels induce apoptosis of cancer cells *via* activation of HIF-1α and its interlinked IGFBP-3 protein. IGFBP-3 inhibits PI3K/Akt/mTOR and triggers apoptosis.

In the same way, excessive ROS levels also initiate apoptosis *via* activation of PI3K/Akt/mTOR signaling pathway by altering the binding activity of various hormones and growth factors to their respective receptors. This evidence-based study specifically highlights the IGF-1 and its role in apoptosis. Several reports suggest that excessive intracellular ROS production trigger HIF-1α mediated apoptosis in cancer cells *via* activation of IGF-1 binding protein 3 (IGFBP-3) and inhibition of IGF-1 signaling. This step blocks the binding of IGF-1 to its receptor and subsequently inactivates the downstream signaling molecules like PI3K, Akt, and mTOR that help in tumor growth and progression, shown in **Figure 5**. Finally, this inhibits the NF-κB protein and promotes apoptosis. Therefore, the phytocompounds like alkaloids that induce ROS and modulate different kinds of signaling molecules, including hormone-related growth factors, can be exploited in colon cancer treatment (57).

## 5 Current treatment strategies of colon cancer and their limitations

Several studies revealed that colon cancer treatment depends on the tumor stage and location. In the early stage, a tumor can be surgically eliminated from the site. However, in the advanced stage, chemotherapy as a single drug or combined with other drugs or radiation is the only option. Among the chemotherapy drugs, fluoropyrimidines have been highly recommended for the treatment of colon cancer. However, fluoropyrimidine derivatives like 5-fluorouracil alone have not been found to be effective. In addition, it also exhibited multiple side effects such as neutropenia, stomatitis, nausea, alopecia, photosensitivity, cardiotoxicity, and sub-acute multifocal leukoencephalopathy. Therefore, its usage as a single drug is limited for colon cancer treatment (58). Subsequently, a platinum derivative named oxaliplatin has been introduced in the standard colon cancer treatment, and it was co-administered with 5-fluorouracil and leucovorin (folinic acid) to reduce the toxicity. This drug has specifically been used in the advanced stage of colon cancer. However, in clinical studies, oxaliplatin drug exhibited characteristic side effects as compared to other platinum derivatives including generation of neurotoxicity, hematotoxicity, gastrointestinal tract toxicity, neurolopathy, thrombocytopenia and nephrotoxicity. In addition to these toxicities, oxaliplatin increases CRC patients’ death rate when included in the treatment regimen (58, 59). Irinotecan hydrochloride, a camptothecin derivative, was introduced to improve the CRC treatment. This drug has been recommended in combination with 5-fluorouracil, leucovorin, and oxaliplatin to treat metastatic CRC. Interestingly, this combination increased the survival time by more than 30 months. However, the active metabolite of irinotecan (SN-38), an active metabolite of irinotecan hydrochloride showed severe toxicity, including neutropenia, by developing polymorphic glucuronidation. Additionally, two monoclonal antibodies such as cetuximab and bevacizumab, belonging to the class of EGFR and angiogenesis antagonists, respectively, have been added to the irinotecan hydrochloride treatment strategy. Cetuximab was also reported to specifically bind to and inhibit EGFR, whereas bevacizumab inhibited VEGF. Together with an irinotecan hydrochloride treatment regimen (comprising 5-fluorouracil/leucovorin), both cetuximab and bevacizumab are considered helpful in managing metastatic CRC patients. The inclusion of cetuximab can significantly reduce the progression of metastatic CRC. On the other hand, the inclusion of bevacizumab in the irinotecan hydrochloride treatment regimen can increase the survival rate of CRC patients by over 4 months as compared to other standard treatments. However, cetuximab, as a single drug or combined with irinotecan treatment, was associated with various adverse effects such as asthenia, leukopenia, abdominal pain, and neutropenia (60). Similarly, adverse effects like gastrointestinal hemorrhage and bowel ischemia have been associated with bevacizumab (61). Besides, panitumumab, another monoclonal antibody, and an EGFR inhibitor, have recently been introduced specifically to treat metastatic CRC patients with RAS wild type *via* interaction with PI3K/Akt/mTOR signaling molecules. This drug also showed severe toxicity related to skin including dermatitis, pruritus, paronychia, and erythema (62).

In addition, capecitabine, the first oral prodrug of 5-fluorouracil, was used in combination with intravenous irinotecan. However, this combination was administered with or without bevacizumab to treat metastatic CRC. Capecitabine administration was associated with toxicities such as hand-foot syndrome, leukopenia, and proteinuria. In addition, this drug has also shown toxicities like 5-fluorouracil (63, 64). Another prodrug of 5-fluorouracil, known as tegafur, is orally used to treat colorectal cancer liver metastases in combination with 5-fluorouracil. This combination blocks the crucial metabolizing enzyme of 5-fluorouracil and increases the serum concentration of 5-fluorouracil. Hence, in the current treatment regimen, this combination along with oral leucovorin has been included as a typical treatment for stage III colon cancer. Moreover, tegafur also shows serious side effects like neutropenia, thrombocytopenia, mucositis, and asthenia (65, 66). Recently, regorafenib, a kinase inhibitor has been approved for the treatment of metastatic CRC. This drug inhibits various kinases such as fms-related receptor tyrosine kinase 1 (FLT1), TEK receptor tyrosine kinase, rapidly accelerated fibrosarcoma (Raf-1) proto-oncogene, KIT proto-oncogene receptor tyrosine kinase, serine/threonine kinases, and kinase insert domain receptor (KDR). This drug has also proved its efficacy by increasing the survival time by 2.5 months. Moreover, this drug shows few side effects compared to other currently used chemotherapeutic drugs (64). Recently, another monoclonal antibody named dostarlimab, an anti-programmed cell death protein PD-1, has been introduced in colon cancer treatment regimen. The clinical study results show that dostarlimab can completely eradicate colon cancer. Moreover, this drug has also exhibited minimal toxicity in the CRC patients (67). Another monoclonal antibody, a PD-1 inhibitor, nivolumab was introduced to treat microsatellite instability high CRC. The study results revealed that nivolumab possess a better disease control rate of microsatellite instability high CRC (68). At the same time, when co-administrated with ipilimumab (a CTLA4 inhibitor), nivolumab improved cancer conditions and showed 80% disease control rate (69–71). Similarly, another monoclonal antibody named prembrolizumab was introduced to efficiently treat microsatellite instability high defective DNA mismatch repair CRC with a much higher rate of overall response and progression free survival rate. In the study, prembrolizumab has been found to increase the survival time by 8.3 months and the overall survival rate by 43.8% (72). However, almost all existing drugs, including immunotherapy, sooner or later have been becoming less effective, mainly due to resistance, associated toxicities, and immune escape (73, 74), as detailed in **Table 1**. Hence, there is an urgent need to develop a potential therapeutic tool against CRC. Among the various sources, medicinal plants and their derived compounds play essential roles in drug discovery. They show various pharmacological functions, including anti-cancer and immunomodulatory effects. Several plant derived compounds as such or combined with other clinical drugs have been used to treat various cancers. Phytocompounds like berberine, epigallocatechin, and lycopene have recently undergone clinical trials to manage colon cancer at different stages. Hence, this evidence-based study highlights phytochemicals, specifically the alkaloids that induce ROS-mediated apoptosis.

**Table 1:** Problems associated with current drug therapies for colon cancer.

Drug	Side effects/Adverse effects	Reference
Fluoropyrimidine (5-fluorouracil)	Neutropenia, Stomatitis, Nausea, Alopecia, Photosensitivity, Cardiotoxicity, and Sub-acute multifocal leukoencephalopathy	(58)
Oxaliplatin	Neurotoxicity, Hematotoxicity, Gastrointestinal tract toxicity, Neurolopathy, Thrombocytopenia and Nephrotoxicity. Increases the death rate of CRC patient.	(58, 59)
Irinotecan hydrochloride	SN-38, an active metabolite of irinotecan shows side effects like Neutropenia due to polymorphic glucuronidation	(58, 59)
Cetuximab	Asthenia, Leukopenia, Abdominal pain, and Neutropenia	(60)
Bevacizumab	Gastrointestinal hemorrhage and Bowel ischemia	(61)
Panitumumab	Severe skin toxicities including Dermatitis, Pruritus, paronychia, and Erythema	(62)
Capecitabine	Hand-foot syndrome, Leukopenia, and Proteinuria	(63, 64)
Tegafur	Neutropenia, Thrombocytopenia, Mucositis, and Asthenia	(65, 66)

## 6 Role of phytocompounds in inducing ROS mediated apoptosis in colon cancer

Cancer is the second leading cause of mortality and a substantial global health concern. Despite significant advancements in treatment modalities, cases of various cancers and deaths have still been on the rise. In 2020, nearly 19.3 million new cancer cases and 10 million new deaths have been reported, accounting for the extreme increase of cases and deaths worldwide (75). Among the various cancers, CRC stood second in terms of cancer-related deaths (6, 75). This continuous rise in CRC cases is due to the lack of efficient screening and treatment methods. In addition, colon cancers were being detected at an advanced stage or at metastasis stage in most cases, which made it difficult to treat the patients. Moreover, currently recommended treatments such as surgery, radiation, chemotherapy, and combination treatments also exhibit serious challenges like non-specificity to cancer cells, side effects as well as drug resistance, thus limiting the efficacy of various treatment options (76).

Given the severity of the disease, there is an urgent need to find a novel treatment with minimal toxicity to the CRC patients. Due to the never-matched chemical library and other favorable factors, medicinal plants and derived phytochemicals act as potential resources for novel drug discovery against various diseases, including cancer (77, 78). In fact, plant secondary metabolites such as alkaloids, terpenoids, flavonoids, steroids, saponins, and phenolic compounds have been held responsible for their antitumor activity as well as other biological functions of the medicinal plants (76, 79, 80). For example, taxol, vincristine, vinblastine, and podophyllotoxin form the primary secondary metabolites with proven anticancer potentials *via* modulating various signaling molecules of death pathways (78, 81, 82). However, studies revealed that in most cases these anticancer phytochemicals also show toxicity towards non-cancerous cells, which is a serious challenge to the treatment (83). Hence, there is an urgent need for unique and ideal therapeutic tools that can selectively target cancer cells alone.

Generally, cancer cells require a high level of ROS to run normal biological functions such as differentiation and development and maintain an increased metabolic rate. In addition, ROS at the required level also help in tumor progression, metastasis, and angiogenesis. However, the level of ROS exceeds the limit, leading to oxidative damage to the cells, specifically in cancer cells (27). This discrete damage to cancer cells alone is due to the increased mitochondrial dysfunction, which makes the cancer cells more sensitive to oxidative stress than the normal cells. Hence, a slight increase in ROS levels by using external agents can induce cell death through various death mechanisms in cancer cells. Among the different kinds of programmed cell deaths, apoptosis is a well-studied and vital pathway involved in several diseases, including cancer. In cancer, the malignant cells maintain shallow levels of apoptosis, which result in tumor growth and progression. Hence, forced activation of apoptotic signaling molecules *via* up-regulation of ROS through introduction of small molecules from various sources can help to treat cancer better. Mounting evidence also suggests that medicinal plants and their derived components are better sources for novel drug discovery because of their minimal toxic effects.

Among the several classes of phytocompounds, alkaloids serve as a vital source for identification of novel drug candidates against various diseases including cancer. In addition, they can also induce ROS-mediated cell death by regulating various intrinsic and extrinsic apoptotic signaling molecules in different cancers, including colon cancer (24, 84). Alkaloid compounds that induce ROS-mediated apoptosis in colorectal cancer have been discussed in detail. Additionally, various classes of phytocompounds that initiate ROS-mediated apoptosis in different cancer cells are mentioned in **Supplementary Tables 1–6**. In this way, the current evidence-based review serves as a complete reference for all kinds of phytochemicals that promote ROS-associated apoptosis *via* targeting various signaling molecules, including IGF-1 in cancer cells.

### 6.1 Alkaloids as anti-colon cancer agent

Alkaloids are plant-derived naturally occurring secondary metabolites. These compounds mostly contain nitrogen as the heteroatom, which imparts basic nature to these molecules. There are several kinds of alkaloids based on heterocyclic rings that show numerous medicinal potentials against malaria, neurodegenerative diseases, viral infections, arrhythmia, hepatitis, asthma, bacterial infections, hypertension, eye infections as well as different kinds of cancers, including colon cancer (85). Various kinds of alkaloids that induce ROS-mediated apoptosis in colon cancer, either alone or in combination with other drugs, are discussed below.

#### 6.1.1 Isoquinoline alkaloids

These compounds are known to show potential anti-colon cancer activity *via* targeting cancer cells alone through initiating ROS-dependent apoptosis. Evidence suggests that benzophenanthridine alkaloids belonging to the class of isoquinoline type of alkaloids can trigger ROS-mediated cell death in cancer cells. Among this class of compounds, sanguinarine is a naturally derived alkaloid isolated from *Macleaya cordata*, belonging to the family Papaveraceae. This compound killed the colon cancer cells like human colon adenocarcinoma (SW480) and human colon cancer (HCT116), as well as reduced the SW480 and HCT-116 cell-generated tumor growth in an orthotopic model of nude BALB/c mice. Sanguinarine also induced intrinsic apoptotic cell death in colon cancer cells *via* increasing ROS-mediated mitochondrial permeabilization and releasing important signaling molecules like cytochrome c and ATP. In fact, apoptosis was initiated through the activation of Bax (dependent), which disrupted the association of serine-threonine kinase receptor-associated protein (STRAP) and maternal embryonic leucine zipper kinase (MELK) proteins. Then ultimately activated the caspase 3 protein and its function (86). In another study, sanguinarine induced ROS mediated apoptotic cell death in colon cancer cells through DNA damage wherein the apoptotic activity was p53 independent (87). In addition, 6-methoxydihydrosanguinarine, a natural sanguinarine derivative, induces apoptotic cell death in various cancer cells *via* ROS production (88). Berberine, another isoquinoline type of alkaloid isolated from the Berberis genus, also produces an anti-colon cancer effect by upregulating ROS and apoptotic signals. In a study, this compound was reported to trigger apoptosis through ROS generation in colon cancer cells like human colorectal adenocarcinoma (HT-29) and HCT-116 cells *via* increasing the expression of lnc RNA cancer susceptibility candidate 2 (lncRNAs CASC2). lncRNA CASC2 later interacts with AU-rich element RNA-binding factor 1 (AUF1) to block the translation of Bcl-2 triggering apoptosis (89). This compound also initiated apoptotic mediated cell death in mouse conditionally immortalized epithelial (IMCE) cells that were inherited with adenomatous polyposis coli (Apc) multiple intestinal neoplasias (min) mutation. This investigation also revealed that berberine kills IMCE *via* inducing ROS, which initiates the release of AIF from mitochondria and triggers apoptosis in caspase-independent manner. Moreover, berberine did not show toxicity towards normal colon epithelial cells i.e., young adult mouse colon epithelial cells (90). In addition, berberine inhibits the migration of SW480 and HCT-116 cells *via* activation of AMPK through upregulation of ROS and downregulation of glucose levels. Thus activated AMPK decreases the activity of integrin β1 protein and initiates cell death (91). In another investigation, berberine showed ROS-mediated apoptosis in human colon carcinoma (SW620) cells by increasing JNK, c-Jun, and p38 MAPK phosphorylation. On the other hand, these compounds also enhance the release of cytochrome c and activate caspases 3 and 8 to support Fas-mediated apoptosis (92). Li and the team also studied the effect of berberine on azoxymethane or dextran sulfate sodium-induced colon tumors in mice. In their study, berberine reduced the tumor size to 60% *via* activation of AMPK and reduction of mTOR activities. They concluded that the tumor regression activity is associated with reduced activity of cyclin D1, nonhistone nuclear protein (Ki-67), cyclooxygenase-2 (COX-2), survivin, NF-κB, and increased activity of caspase-3 function. This compound also inhibits the migration and metastasis of SW620 and human colon cancer cell line (LoVo) cells *via* down-regulating the expression of COX-2, prostaglandin E2 (PGE2), Janus kinase 2 (JAK2), and STAT3 signaling molecules. Besides, it also suppresses the solid tumor growth in BALB/c mice and lung metastasis developed by SW620 and LoVo cells (93, 94). On the other hand, berberine inhibits colon tumor growth *via* targeting signaling molecules like TNF-α, EGFR, and Wnt/β-catenin signals (95–97). Furthermore, recent combination studies of berberine with *Andrographis paniculata* extract also showed an excellent anti-colon cancer effect, where they inhibited the growth of HT-29 cells and colon carcinoma cell line (RKO) cells and their corresponding *in vivo* tumor growth. This activity was initiated by an increase in the ROS levels, which finally altered the genes involved in DNA replication (98). Furthermore, when combined with evodamine an indole alkaloid, berberine enhances the anti-tumor activity. This combination promotes apoptosis in cancer coli-2 (Caco-2) and HT-29 cells *via* modulation of Nrf2-mediated pathways (99). Cepharanthine, a biscoclurine or bis benzylisoquinoline alkaloid extracted from *Stephania cepharantha Hayanta*, also significantly induced oxidative stress-mediated apoptosis in p53 mutated colon cancer cells HT-29 and SW620 cells. Cepharanthine initiated apoptosis in HT-29 cells, which is linked with cell cycle arrest at growth 1 (G1) phase and cyclin-dependent kinase inhibitor 1 or CDK-interacting protein 1 (p21Waf1/Cip1) level increase (100). Coptisine, another isoquinoline alkaloid extracted from *Coptis chinensis Franch*, reportedly triggered ROS-dependent apoptosis in HCT-116 cells. It reduced the growth and migration and finally promoted caspase-mediated apoptosis in HCT-116 cells by altering mitochondrial membrane potential as well as modulating various signaling molecules like Bcl-2, Bcl-XL, XIAP, Bax, Bad, cytochrome c, Apaf-1, AIF, caspase-3, PI3K and Akt. In addition, coptisine reduces the HCT-116 generated tumor growth by inducing apoptosis in nude BALB/c mice (101). In another study, this compound was reported to induce cell cycle arrest at G1 phase as well as inhibit HCT-116 xenograft tumor growth *via* targeting Milk Fat Globule EGF And Factor V/VIII Domain Containing (MFG-E8) gene and rat sarcoma- extracellular signal-regulated kinase (RAS-ERK) pathways (101–103). Moreover, alkaloids like neferine and isoliensinine isolated from *Nelumbo nucifera* (lotus) plants have shown significant anticancer potential by enhancing the chemo- sensitivity of colon cancer cells towards cisplatin treatment. These two alkaloids and cisplatin increased the ROS levels in human CRC (HCT-15) cells, leading to caspase-3 dependent apoptosis through the deactivation of MAPK/PI3K/Akt/mTOR signaling molecules. In addition, this combination was also found to decrease the resistance towards cisplatin and increase apoptotic markers in colon cancer stem cells *via* uplifting Ca^2+^ level and damaging mitochondrial membrane integrity (104, 105). Recent studies revealed that palmatine, another isoquinoline alkaloid, induces apoptosis in colon cancer cells like HCT-116, SW480, HT-29, and, in HCT-116 cell xenograft tumor *via* increasing ROS levels and decreasing mitochondrial membrane potential. This leads to release of cytochrome c protein. Palmatine is believed to target various signaling molecules such as aurora kinase A (AURKA), Bcl-xl, Bcl2, caspase 3 and 9 to induce apoptosis (106). Tetrandrine, a bisbenzylisoquinoline alkaloid derived from the roots of *Stephania tetrandra*, also triggers oxidative stress-mediated cell death in HCT-116 cells through modulation of E2F transcription factor 1 (E2F1) and p53/p21Cip1 levels (107, 108). Nano formulation of this alkaloid showed an enhanced anticancer effect against Lovo cells through ROS-mediated upregulation of JNK and caspase-3 activity (109). Similarly, xylopine, an isoquinoline alkaloid isolated from the stem of *Xylopia laevigata*, also induces oxidative stress-mediated apoptosis in HCT-116 cells by triggering apoptosis in p53 independent manner *via* induction of cell cycle arrest at growth 2 (G2)/mitosis and cytokinesis (M) phase, and caspase-3 activity (110). For more clarification, the above-mentioned compounds are presented in the **Supplementary Table 7**.

#### 6.1.2 Indole alkaloids

Indole alkaloids also show a prominent anticancer effect. Camptothecin, a well-studied compound under the class of indole alkaloids, extracted from *Camptotheca acuminata* belonging to the family Nyssaceae showed potent anticancer effects (111). Evidence suggests that this molecule exhibits anti-tumor activity against various cancer cells, including colon cancer. Notably, the anticancer effect of camptothecin is associated with the apoptosis mechanism, which is mediated by ROS (112). A study by Park and colleagues reported that camptothecin kills the HCT-116 cells through activating apoptosis signaling associated with TNF-related apoptosis-inducing ligand (TRAIL). This study also found that camptothecin induces DNA damage and activates Bax, p53, and p21 proteins. This step finally leads to the death of colon cancer cells. Their investigation also proved that hypoxia condition reduces the apoptotic cell death of HCT 116 cells *via* decreasing Bax. The study concluded that ROS are essential for camptothecin’s anti-tumor activity (113). In addition, camptothecin, along with other chemotherapeutic drugs, initiates apoptosis in colorectal adenocarcinoma (DLD1) and HCT-116 colon cancer cells *via* increasing mitochondrial fission and decreasing complex I activity as well as mitochondrial size. This mitochondrial change provokes excess ROS production, which helps to activate apoptotic signaling (114). Furthermore, Wenzel and coworkers found that camptothecin at 50µM can induce apoptotic cell death in HT-29 colon cancer cells through upregulating caspase-3 activity *via* increasing mitochondrial superoxide ROS production. This caspase-3 activation triggers the apoptotic characteristics, like loss of plasma membrane integrity and nuclear fragmentation, in the HT-29 cells. In addition, this study also revealed that camptothecin modulates various signaling molecules like Bcl-2, Bax, Bak, p21, COX-2 and NF-κB in HT-29 cells, which promote apoptosis. However, ascorbic acid treatment at 50uM reverses the changes made by camptothecin in various signals and inhibits the apoptosis process *via* scavenging the superoxide radicals in HT-29 cells (115). In another study, Guo et al. showed that prodrug formulation of camptothecin *via* conjugating with lysine or arginine amino acids enhances water solubility, tumor penetration capacity as well as pharmacokinetic properties. This study also proved that prodrug formulation of camptothecin increases the mitochondrial ROS production much better than the camptothecin alone, thereby decreasing the murine colorectal carcinoma (CT-26) cells induced tumor size in Bagg albino (BALB/c) mice *via* initiating apoptosis process (116). Besides, the semi-synthetic derivative of camptothecin like irinotecan hydrochloride (IH) also shows anti-colon cancer activity. Moreover, SN-38, the active metabolite of IH, significantly improves the patients’ colon cancer condition and survival rate up to 30 months. Nowadays, IH is highly recommended to treat metastatic CRC in combination with other clinical drugs like 5-fluorouracil and oxaliplatin (117). A study conducted by Britten and his colleagues showed that irinotecan reduces the growth of HT-29 cells generated tumors drastically when co-administered with other compounds like 6-hydroxymethylacylfulvene and 5-fluorouracil (118). In another experiment, Allegrini and his team demonstrated that irinotecan, when co-administered with the antiangiogenic drug thrombospondin-1 (TS-1) to nude mice bearing HT-29 cells generated tumor, a significant reduction in the size of the tumor occurs as compared to the control as well as individual treatment. At the same time combination of TS-1 with irinotecan did not affect much growth of HT-29 cells (119, 120). Moreover, this combination is non-toxic to the normal cells. In another study, an anti-folate agent used in combination with SN-38, an active metabolite of irinotecan to treat 5-fluorouracil resistant HT-29 cells and found a significant growth inhibition of the cells. This combination also potentially reduced the HT-29 cell-oriented solid tumor in BALB/c nude mice (119, 120). Results from the clinical study also revealed that when irinotecan was given in combination with oxaliplatin, capecitabine, and bevacizumab for 8 cycles to a patient suffering from advanced tumor provided promising results by increasing the survival time. Similarly, irinotecan co-administered with 5-fluorouracil and oxaliplatin also showed a significant recovery in advanced colon cancer patients (121, 122). Additionally, all the indole alkaloids that induce ROS mediated apoptosis in colon cancer are presented in **Supplementary Table 7** for detailed clarification.

#### 6.1.3 Capsaicinoids

Capsaicinoids are another alkaloid class that induce ROS-mediated apoptosis in colon cancer cells. Capsaicin is a significant alkaloid that belongs to the class of capsaicinoids, reported to up-regulate apoptotic signals in colon cancer cells through increasing intercellular ROS (123). In a study conducted by Yang and his team, capsaicin was found to reduce the viability of colon cancer cells like human colon carcinoma (Colo320DM) and LoVo cells through initiating caspase-dependent apoptosis, which is linked with an increase in intracellular ROS production and altering mitochondrial membrane potentials (124). In another investigation, a group of researchers reported that capsaicin can trigger apoptosis in HT-29 colon cancer cells *via* activating peroxisome proliferator-activated receptor –γ (PPAR- γ) as well as AMPK but not the vanilloid receptor (125). Lu et al. showed that this compound induces apoptotic cell death in colo 205 cells as well as its corresponding tumor xenograft through targeting various signaling molecules like Fas, cytochrome c, Bax, Bcl-2 p53, p21, and caspases 3, 8 and 9. In addition, this apoptotic activity is strongly associated with increased intracellular ROS, Ca^2+^ production, and damage to mitochondrial membrane integrity (125–127). Furthermore, capsaicin in combination with 3,3′- diindolylmethane enhances the anti-cancer potential by inducing apoptosis in colon cancer cells like HCT116, SW480, LoVo, Caco-2, and HT-29 *via* targeting apoptotic markers like Fas, NF-κB, p53 and other proteins (128). Capsaicin, combined with resveratrol, have also been reported to induce apoptotic cell death in HCT-116 cells by increasing nitric oxide (NO) concentration and p53 level. The increase in p53 level decreases murine double minute 2 (Mdm2) and increases Bax expression to promote apoptosis (129). **Supplementary Table 7** provides further information on capsaicinoids.

#### 6.1.4 Carbazole alkaloids

Carbazole alkaloids constitute another plant-based alkaloid class, exhibiting an anti-colon cancer effect. Clausenidin is the one belonging to the carbazole alkaloid class, isolated from the roots of *Clausena excavata* and also reported to initiate caspase-9 dependent apoptosis in HT-29 cells through cell cycle arrest at quiescent phase (G0)/G1 phase, triggered by increasing ROS mediated mitochondrial membrane depolarization (130). More details can be found in **Supplementary Table 7**.

#### 6.1.5 Diterpenoid alkaloids

Besides, diterpenoid alkaloids too exhibit anti-colon cancer effect (**Supplementary Table 7**). Lappaconitine hydrochloride (LH) is one such compound and a derivative of C 18-diterpenoid alkaloid. Song and colleagues suggested that this compound demonstrates an anti-colon cancer effect by inhibiting proliferation and increasing apoptosis in HCT-116 cells. Moreover, this activity is associated with increased ROS and decreased mitochondrial membrane potential. They also found that LH reduces the tumor volume of the HCT-116 cells-oriented xenograft model *via* altering the MAPK pathway (131).

#### 6.1.6 Piperidine alkaloids

Piperine, a piperidine alkaloid obtained from black pepper also reported to trigger apoptotic cell death in HT-29 cells through cell cycle arrest at the G1 phase (**Supplementary Table 7**). This activity is achieved *via* targeting various markers, including cyclin D1, D2, and cyclin-dependent kinase inhibitor (p21^WAF1^ and p27^KIP1^) (132).

#### 6.1.7 Amide alkaloids

Amide alkaloids are an essential group of alkaloids derived from plants. They also show potent anti-colon cancer effects (**Supplementary Table 7**). Piperlongumine is a critical compound from this class, extracted from the fruits of *Piper* species. Reports suggested this compound also induces apoptosis in HCT-116 *via* increasing ROS and decreasing antioxidant enzyme glutathione S-transferase. It targets JNK, MEK, and ERK proteins but not Bax, p21, and p53 to induce selective apoptosis in colon cancer cells. In addition, piperlongumine is also used in combination with oxaliplatin, a first-line drug of choice for metastatic colon cancer. This combination enhanced anti-colon cancer activity by increasing the level of ROS-mediated apoptosis, which is linked with upregulation of mitochondrial dysfunction and ER related apoptotic markers in HCT-116 and LoVo cells (133, 134).

#### 6.1.8 Carboline alkaloids

Carboline alkaloids also show prominent anti-cancer potential (**Supplementary Table 7**). Kim et al. (135) reported another alkaloid harmine, a carboline derivative, extracted from *Peganum harmala*. This compound can induce ROS-dependent apoptosis in HCT-116 cells. In a study, they investigated the anti-colon cancer potential of harmine hydrochloride. This compound found found to activate apoptotic cell death in HCT-116 cells through increasing apoptotic [capases 3 and 9, poly (ADP-ribose) polymerases (PARP)] as well as pro-apoptotic proteins (Bax) and decreasing anti-apoptotic protein (Bcl-2) markers. These investigations also revealed that the harmine hydrochloride compound mainly alters ERK/PI3K/Akt/mTOR signaling pathway to initiate apoptosis. In addition, this compound also triggered apoptosis in SW620 cells by inducing arrest at the sub-G1 phase and decreasing mitochondrial membrane potential. Notably, harmine promotes caspase-dependent apoptotic cell death in SW620 cells through downregulation of ERK/PI3K/Akt/mTOR-related pathways (45, 135, 136).

#### 6.1.9 Quinolizidine alkaloids

Likewise, a study revealed the anti-colon cancer effect of oxymatrine (**Supplementary Table 7**), a quinolizidine alkaloid extracted from the roots of *Sophora flavescens* – a Chinese traditional medicine. These compounds induce apoptosis in colon cancer cells *via* ROS generation. Moreover, earlier reports suggests that co-administration of oxymatrine with doxorubicin produces a better anti-colon cancer effect *via* enhancing apoptotic signals (cleaved caspase-3, cleaved caspase-9 and Bax/Bcl-2 ratio) in HT-29 and SW620 cells mediated by ROS generation. In addition, this combination also reduces the growth of HT-29 generated tumor xenograft in a dose dependent way *via* modulating E-cadherin and N-cadherin level. Besides, oxymatrine also increases the sensitivity of 5-fluorouracil resistant cells like HCT-8 and initiates apoptosis by promoting inhibition of EMT and NF-κB activity (137, 138).

#### 6.1.10 Proto alkaloids

In a few studies, researchers reported colchicine, a proto alkaloid, to show an anti-colon cancer effect through up-regulation of ROS and its associated apoptotic markers. They showed that colchicine induces apoptosis in HT-29 cells by regulating ROS and decreasing mitochondrial membrane integrity. This compound was ultimately reported to trigger apoptosis by activating BAX, caspase 3, p38 and deactivating AKT signaling molecules. In another investigation, this compound reportedly initiated ROS-mediated apoptosis in HCT-116 and Colo-205 cells by depolymerizing microtubules and arresting the cell cycle at the G2/M transition state (139, 140).

### 6.2 Alkaloids and other phytocompounds in clinical trials against colorectal cancer

Existing drugs for colon cancer treatment have been showing various side effects, including resistance. Therefore, there is a rise in demand for novel therapeutics to overcome the toxicities and limitations (2). Multiple pieces of evidence support medicinal plants and their derived phytochemicals as a suitable and potent source of drug discovery against various diseases. In this evidence-based study, various classes of phytocompounds have been discussed, including the alkaloids that are of clinical value for colon cancer treatment. Such compounds include berberine, epigallocatechin-3-gallate (EGCG), curcumin, lycopene, fisetin, and resveratrol. These compounds have recently been used in different phases of clinical trials against CRC. In a clinical study, a patient suffering from CRC was advised to take 0.3 g of berberine daily two times, and there was no sign of a tumor in the later stage. Berberine was strongly recommended to decrease the chances of recurrence of colorectal adenoma and polypoid lesions. Moreover, this drug was safe and showed no severe side effect (33). In addition, EGCG is also in the early stage of CRC clinical trials. The chemopreventive effect of Teavigo (purified tea extract with 94% EGCG) has been evaluated. CRC patients with curative resections were administered orally with 450 mg of EGCG twice daily. Blood was drawn from the patients at 0, 3, 6, 9, 12, 15, and 18 months and a colonoscopy was done after a year (141). Furthermore, lycopene, a carotenoid, was also tested against metastatic CRC and was found to decrease skin toxicity associated disease as a single drug or in combination with panitumumab (31). Likewise, resveratrol, a stilbenoid compound, was also studied against metastatic CRC with liver metastases. A 5.0 g daily dose of the compounds administered for two weeks, could markedly induce caspase-dependent apoptosis in metastatic liver cells of CRC patients (142, 143).

Similarly, curcumin, a polyphenol compound, was also effective against various cancers, including CRC. In addition, some other phytocompounds that act as immune boosters or immune modulators help in blocking colon tumor progression. One such compound is fisetin, a flavonoid compound, evaluated in patients suffering from CRC for its anti-inflammatory effect. CRC patients under chemotherapy were fed with 100 mg of fisetin for 7 weeks. At the end of chemotherapy, various inflammatory markers were evaluated, and fisetin was found to reduce the levels of IL-8 and high-sensitivity C-reactive protein (hs-CRP) in CRC patients. Hence, fisetin may be suggested as an adjuvant treatment to the conventional therapies followed to CRC patients (32). Other details regarding the phytocompounds that are under clinical trials aere provided in **Table 2**.

**Table 2:** Phytocompounds assessed in clinical trials for colon cancer treatment.

Phytocompound	Biological source and Class of the compound	Structure	Information (type of clinical trial, number of patients, period, and outcome)	Status of the study	Reference
Berberine	Plant: *Berberis vulgaris*, *Berberis thunbergii*, *Berberis darwinii*. Family: Berberidaceae. Plant: *Rhizoma coptidis*, Family: Ranunculaceae. Type: Isoquinoline alkaloid	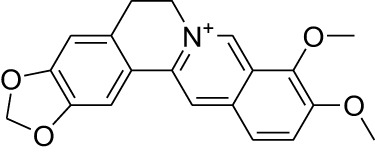	In Phase II clinical study, fixed dose of berberine hydrochloride, reduces the chances of reoccurrence and generation of another new colorectal adenomas. This study was done with 1000 participants for 3 years	Completed	(33), NCT03281096
Camptothecin	Plant: *Camptotheca acuminate*. Family: Nyssaceae. Type: Alkaloid	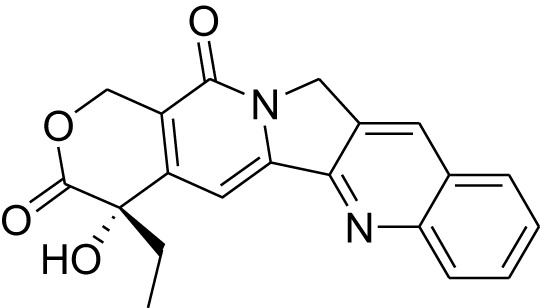	In clinical trials phase II, 100 mg administration of Camptothecin to metastatic colon cancer patients shows partial response in patients with liver metastasis. In this study 67 patients with metastatic colorectal cancer were participated for nearly 2 years.	Completed	(30)
Curcumin	Plant: Curcuma longa. Family: Zingiberaceae. Type: Polyphenol compound	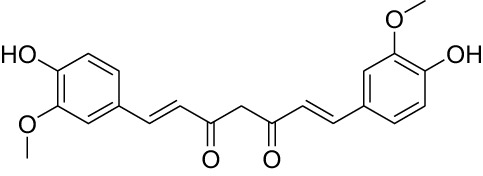	In phase I clinical study, curcumin capsules containing 3.6g of curcumin administered to patients suffering with advanced CRC that shows resistance to available chemotherapy drugs. This study was done on 23 patients suffering with advanced colon tumor were for 4 months.	Completed	(144)
Epigallocatechin	Plant: *Camellia sinensis.* Family: Theaceae. Type: Polyphenol compound	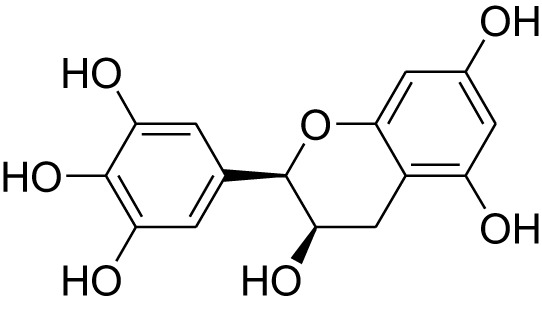	In early phase I clinical trials, Teavigo™ a natural extra purified green tea extract with 94% Epigallocatechin induces alteration in methylation pattern as compared to control. In this study fifty after surgery colon cancer patients were taken part for 1 year study.	Study in progress (estimated date of completion-June, 2024)	(141), NCT02891538
Fisetin	Plant: *Fragaria ananassa*, Family: Rosaceae. Plant: *Malus domestica*, Family: Rosaceae. Type: Flavonoid	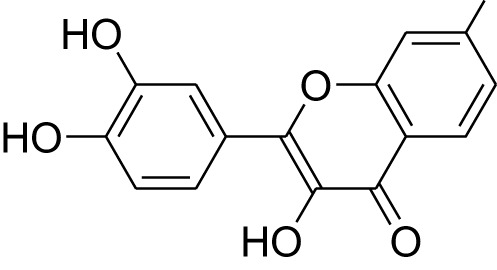	In double-blind, randomized placebo-controlled clinical trial, the fisetin was evaluated for its anti-inflammatory effect in the colon cancer patient receiving chemotherapy treatment. In this study 100mg of fisetin administered to the patient prior to the chemotherapy and found the decreased level of inflammatory markers. This study was done on 37 colon cancer patients for seven weeks.	Completed	(32), IRCT2015110511288N9
Lycopene	Plant: *Solanum lycopersicum*. Family: Solanaceae. Type: Carotenoids	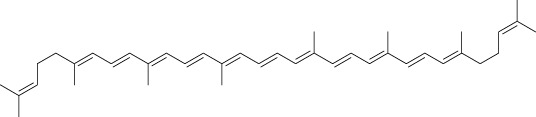	In clinical trials phase II, 20 mg of lycopene reduced the skin toxicity of metastasis CRC patients with panitumumab treatment. In this study 28 patients received with anti-EGFR inhibitor were participated for 12 weeks.	Completed	(31), NCT03167268
Resveratrol	Plant: *Vaccinium uliginosum*, Family: Ericaceae. Plant: *Vitis vinifera*, Family: Vitaceae. Plant: *Ribes nigrum*, Family: Grossulariaceae. Type: Polyphenol compound	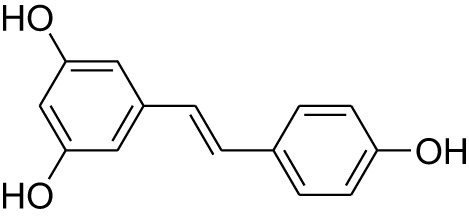	In Phase I clinical study, resveratrol of 20 mg in the form of tablet was administered to the patient with CRC. This compound found to alter Wnt signaling in colon cancer. 11 colon cancer patients were involved in this study for 14 days.	Completed	(142, 143), NCT00256334

## 7 Conclusions and future perspective

Colon cancer globally occupies the third and second positions regarding of prevalence and death rate, respectively. However, there is a vast improvement in the screening and early detection techniques as well as treatment schedules. Therefore, the CRC death rate has slightly decreased in recent times. Yet the incidence of the disease is increasing. This is due to the rise of multiple side effects and limitations in the existing therapies that are followed against CRC. Hence, there is an immediate need for typical, potential, and alternate treatment regimens that can overcome the existing limitations. Several documented evidence suggests that phytocompounds that induce ROS-mediated apoptosis in cancer cells could pose a better and alternative cancer treatment. Moreover, these compounds exhibit minimal toxicity and high specificity to cancer cells in most cases. Their selective cytotoxicity of ROS induced on cancer cells are due to the features like continuous mitochondrial dysfunction and compromised antioxidant defense mechanism. This leads the cancer cells to become more susceptible to oxidative stress. Therefore, slight increase of ROS than the threshold limit by using external agents like phytocompounds can lead the cancer cells to oxidative stress induced apoptotic cell death. However, normal cells can nullify the minimal ROS due to its proper antioxidant defense mechanism and escape from the ROS mediated cell death. Hence, researchers now-a-days focus on plants and their derived compounds that induce ROS for identifying novel drug candidates against various diseases including CRC.

**Figure 6:**
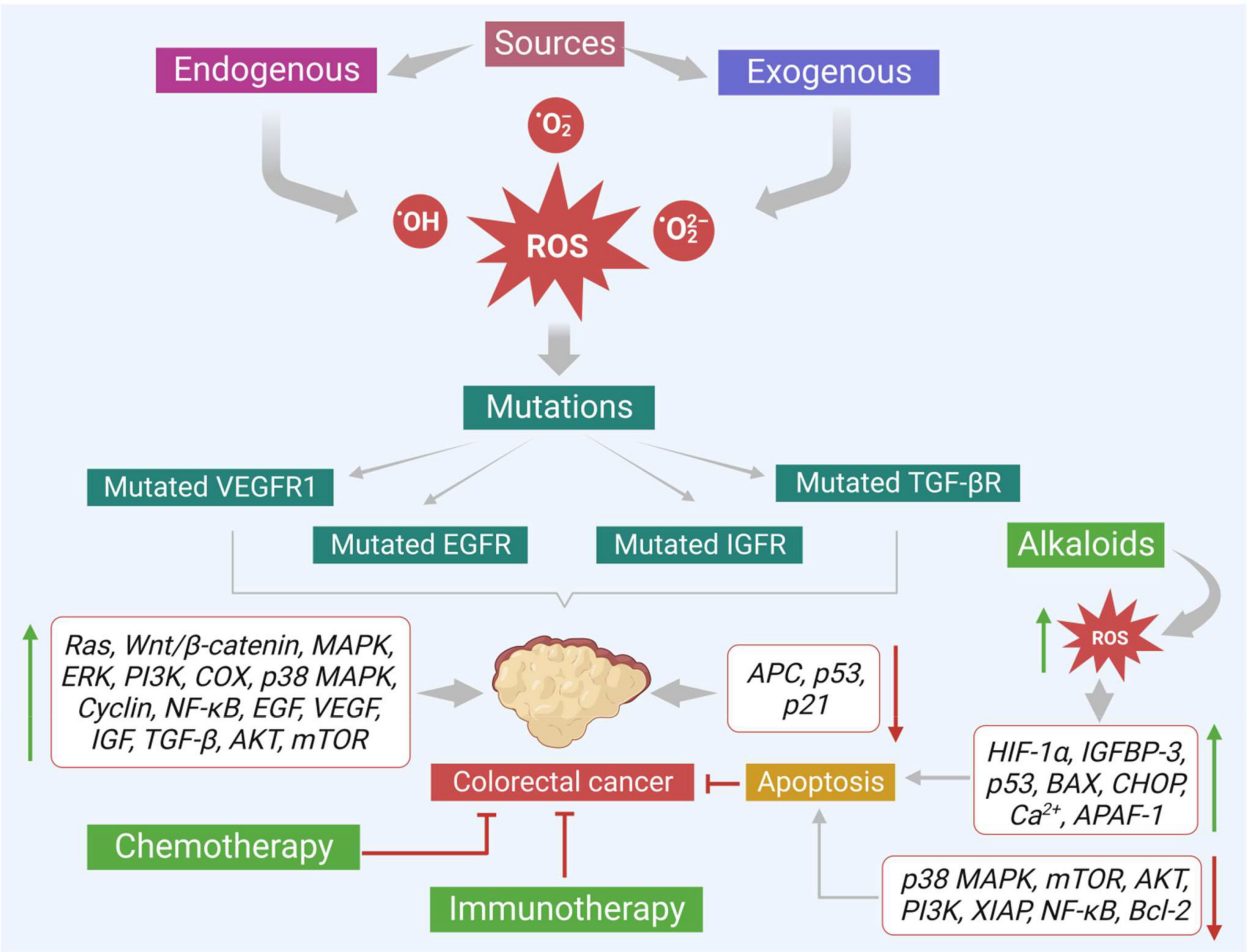
Various sources that promote colon cancer progression. Effects of alkaloids in reducing the colon cancer growth *via* increasing ROS production and modulating the important signals that trigger apoptosis are also shown.

This evidence-based study discusses important plant-based alkaloids that induce cell death *via* ROS-mediated apoptosis in colon cancer. Alkaloids can induce ROS-associated apoptosis *via* altering multiple signaling molecules that involve various vital characters like proliferation (Ki-67, STRAP, MELK, JNK, PI3K/Akt/mTOR/Wnt/β-catenin, ERK, MEK, MAPK-p38), cell cycle (cyclin D1, D2, surviving, c-Jun), inflammation (TNF-α, COX-2, NF-κB), oxidative stress (Nrf2, GSK-3β), tumor suppression (p53, E2F1, AMPK, PPAR- γ), metastasis or angiogenesis (IGF-1, MFG-E8, integrin β1, ERK, EGFR, E-cadherin, N-cadherin, PGE2) and apoptosis (cytochrome C, AIF, Bax, p21, p21WAF1, p27KIP1, p53/p21Cip1, caspases 3 and 9, XIAP, Bcl-2, IncRNA CASC2, AUF1, PARP) of colon cancer cells (**Figure 6**). Many of these alkaloids have shown promising results in various colon cancer preclinical studies. For example, alkaloids like coptisine, camptothecin, capsaicin, lappaconitine, oxymatrine, sophoridine, aleutianamine, and palmatine have shown positive results in different kinds of preclinical models and effectively reduced colon tumor growth. Similarly, preclinical and clinical data suggest berberine (an isoquinoline alkaloid) as a future drug candidate to treat colon cancer. Moreover, berberine works well against CRC and shows negligible toxicity. Furthermore, most alkaloids currently available in the market against various diseases are largely extracted from medicinal plants. Therefore, plant-derived alkaloids provide a potential and novel therapeutic source for effectively managing CRC.

Recent evidence suggests that diet and its related factors (hormonal alteration) are also the leading causes for the generation of 70 to 90% of CRC cases. Food material influences the gut microbiome composition (bacterial strains), which plays a vital role in the colon cancer progression. The gut microbiome is known to be occupied with two significant strains Bacteroides and Prevotella. However, a diet containing fruits and vegetables increases the Prevotella strain in the gut, which can break down the dietary phytochemicals in the lumen into tiny fatty acids like butyric acid. Multiple pieces of evidence revealed that butyric acid exhibits promising anti-cancer and anti-inflammatory effects. Taken together, dietary phytochemicals like alkaloids, which can modulate the gut microbiome, improve the host’s immunity, up regulate the intracellular ROS, and thus could be of promising therapeutic value against CRC. Hence, it may be concluded that consuming diet that contains fruits and vegetables that possess secondary metabolites like alkaloids can better control various kinds of cancers including colon cancer.

## Author contributions

Conceptualization: VKN, SR; Writing—original draft: VKN, MN, JM, MS, NeKJ; Writing—review and editing: VKN, GB, YJ, OA, HS, AM, DN, AA, AJ, KKK, RRM, VM, NiKJ, AK, PS, SR; Illustrations: NiKJ; Supervision: PS, SR. All authors contributed to the article and approved the submitted version.

## Glossary

**Table:**

AIF	Apoptosis-inducing factor
AKT	Protein kinase B
APAF1	Apoptotic peptidase activating factor 1
APC	Adenomatous polyposis coli
ATF2	Activating transcription factor 2
AUF1	U-rich element RNA-binding factor 1
AURKA	Aurora kinase A
BAG	Bcl-2-associated athanogene
BAX	Bcl-2-associated X protein
BRAF	Proto-oncogene
CAD	Caspase-activated DNase
CAMP	Cyclic adenosine monophosphate
CHOP	C/EBP homologous protein
C-MYC	Cellular myelocytomatosis oncogene
DAG	Diacylglycerol
DIABLO	Direct IAP-binding protein with low pI
DNA	Deoxyribonucleic acid
E2F1	E2F transcription factor 1
EGF	Epidermal growth factor
EGFR	Epidermal growth factor receptor
ELK-1	ETS-domain transcription factor
EMT	Epithelial–mesenchymal transition
ERK1/2	Extracellular signal-regulated kinases
ETS	Erythroblast transformation specific
FADD	Fas-associated death domain
FASL	Fas ligand
FOXO3	Forkhead box O3
G2	Growth 2
GSK-3	Glycogen synthase kinase-3
HIF-1α	Hypoxia-inducible factor 1-alpha
HTRA2 (OMI)	Nuclear-encoded mitochondrial serine protease
IGF-1	Insulin-like growth factor 1
IGFBP-3	Insulin-like growth factor (IGF) binding protein-3
IL	Interleukin
JNK	c-Jun N-terminal kinase
KDR	Kinase insert domain receptor
KI-67	Non-histone nuclear protein
α-KGDH	Alpha-ketoglutarate dehydrogenase
lncRNA CASC2	Long non-coding RNA cancer susceptibility candidate 2
MAPK	Mitogen-activated protein kinase
MEK	Mitogen-activated extracellular signal-regulated kinase
MELK	Maternal embryonic leucine zipper kinase
MFG-E8	Milk Fat Globule EGF And Factor V/VIII Domain Containing
MPTP	Mitochondrial permeability transition pore
mTOR	Mammalian target of rapamycin
NF-kB	Nuclear factor-kappa B
NRF2	Nuclear factor erythroid 2-related factor 2
p21Waf1	Cyclin-dependent kinase inhibitor 1 or CDK-interacting protein 1
P38	p38 mitogen-activated protein
P53	Tumor suppressor protein
PDK1	Phosphoinositide-dependent kinase-1
PI3K	Phosphatidylinositol 3-kinases
PIP2	Phosphatidylinositol 4, 5-bisphosphate
PIP3	Phosphatidylinositol (3, 4, 5)-trisphosphate
PLCγ1	Phospholipase C gamma 1
PUMA	p53 upregulated modulator of apoptosis
RAF	Rapidly accelerated fibrosarcoma
RAS	Rat sarcoma
RIP	Receptor-interacting protein kinases
RNA	Ribonucleic acid
ROS	Reactive oxygen species
SMAC	Second mitochondria-derived activator of caspase
SOS1	Son of sevenless homolog 1
STAT3	Signal transducer and activator of transcription 3
STRAP	Serine-threonine kinase receptor-associated protein
TAK1	Transforming growth factor-β (TGF-β)-activated kinase 1
TGF-β	Transforming growth factor beta
TNFR1	Tumor necrosis factor receptor 1
TNFRSF21	Tumor necrosis factor receptor superfamily member 21
TNF-α	Tumor necrosis factor-alpha
TRADD	Tumor necrosis factor receptor type 1-associated death domain protein
TRAIL-R1	TNF-related apoptosis-inducing ligand receptor 1
TRAMP	Tumor necrosis factor (TNF) receptor family
VEGF	Vascular endothelial growth factor receptor
VEGFR-1	Vascular endothelial growth factor receptor 1
XIAP	X-linked inhibitor of apoptosis protein
WNT	Wingless-related integration site.
