# Supplementary material for: Reactive oxygen species mediated apoptotic death of colon cancer cells: therapeutic potential of plant derived alkaloids
Source: Front Endocrinol (Lausanne). 2023 Jul 25;14:1201198. doi: 10.3389/fendo.2023.1201198 (PMC10408138; doi:10.3389/fendo.2023.1201198)
Supplement: Supplementary file 1 [file Table_1.docx]

**Supplementary Table 1: Alkaloids that induce ROS mediated apoptosis in cancer.**

| **Compound** | **Plant origin** | **Structure** | **Mode of action** | **Model (Cell/Animal)** | **References** |
| --- | --- | --- | --- | --- | --- |
| Capsaicin (Capsaicinoids) | *Capsicum annuum* |  | ↑ ROS, ↑TRPV1, ↑Ca^2+^, ↑Mitochondrial dysfunction, ↑Caspase-9 ↓Bcl-2, ↓PI3K, ↓AKT, ↓NF-κB, ↑MAPK, ↑JAK, ↓STAT-3 | Prostate cancer cells - BXPC-3, AsPC-1,  Glioma cells- U251 cells,  Prostate cancer cells- PC-3, LNCaP and RWPE-1 cells & PC-3 Athymic nude male mice Xenograft model | (Pramanik, Boreddy, and Srivastava 2011; NavaneethaKrishnan, Rosales, and Lee 2019; Zhou et al. 2022) |
| Chelerythrine (Phenanthridine alkaloid) | *Chelidonium*  *majus* |  | ↑ROS, ↑ER, ↑UPR ↑GRP78,↑CHOP, ↓STAT3 and ↑caspase activity. | Liver cancer-HepG2 cells. Prostate cancer- PC-3 cells. Lung cancer- NCI-H1703, SK-LU-1 cells. Renal cancer-RCC cells. Lung cancer- H446, H1688 cells. | (Lin et al. 2022; Wu et al. 2018; Heng and Cheah 2020; Yun et al. 2021) |
| Evodiamine (Quinolone alkaloid) | *Evodia rutaecarpa* |  | ↑ROS, ↑MAPK, ↑Mitochondrial Dysfunction, ↑Cytochrome c release, ↓PI3K, ↓AKT | Gastric cancer- BGC-823 cells. Skin cancer- A-375 cells. Thyroid cancer- SW1736, KAT4B cells. Brain cancer- U251, LN229 cells. Urothelial cancer-5637, HT1197 cells. | (Liu, Sun, et al. 2022; Wang, Deng, et al. 2018; Fang et al. 2014; Liu, Li, et al. 2020; Shi et al. 2017; Chien et al. 2021) |
| Neferine (Bis-benzylisoquinoline) | *Nelumbo nucifera* |  | ↑ROS, ↑Mitochondrial Dysfunction, ↑Cytochrome c  ↑Caspase-3, 8 &9, ↑BAX, ↓Bcl-2, ↓PI3K/AKT, ↓MAPK. | Esophageal cancer-KYSE30, KYSE150, KYSE510 cells. Liver cancer- HepG2 cells. Skin cancer- A375, C32 cells & A375 cells Male BALB/c mice xenograft model. | (Poornima, Quency, and Padma 2013; Xie et al. 2020; An et al. 2020) |
| Piperine (Amide alkaloid, piperidine derivative) | *Piper nigrum* |  | ↑ROS, ↑Mitochondrial Dysfunction, ↑Cytochrome c  ↑Caspase-3, 8 &9 ↑JNK, ↑p38, ↑BAX, ↓Bcl-2, ↓PI3K/AKT, ↓ERK, ↓NF-κB, ↓MAPK, ↑JNK, ↑p38, ↓ERK | Skin melanoma cancer cells- SKMEL-28, B16-F0 cells. Liver cancer-Hep G2 cells. Oral cancer- KB cells. | (Fofaria, Kim, and Srivastava 2014) |
| Piperlongumine (Amide alkaloid) | *Piper longum L* |  | ↑ROS, ↑Mitochondrial Dysfunction, ↑Cytochrome c  ↑Caspase-3, 8 &9, ↓PI3K/AKT, ↑Nrf2, ↓ERα | Thyroid cancer- IHH-4, WRO, 8505c, KMH-2 cells. Intestinal cancer-INT-407 cells. Liver cancer- HUH-7, HepG2 cells. Breast cancer-MCF-7, BT474 cells & MDA MB-231 cells female NOD SCID gamma Xenograft model . Ovarian cancer- A2780, OVCAR3, SKOV3 cells. Bone cancer- MG-63, 143B, KHOS/NP (mouse) cells.  Prostate cancer- DU145 cells | (Kung et al. 2021; Rawat et al. 2020; Nelson et al. 2022; Jin et al. 2014; Li, Sharkey, and King 2015; Rawat and Nayak 2022; Zhang, Chen, et al. 2022; Gong et al. 2014; Jeong et al. 2019). |
| Berberine (Isoquinoline, Protoberberine type of alkaloid) | *Coptis chinensis* |  | ↑ROS, ↑DNA Damage, ↑p53, ↑Bax, ↓Bcl-2, ↑Cytochrome c release, ↑Caspase-9, ↑Caspase-3 | Renal cancer cells- ACHN and 786-O | (Lopes et al. 2020) |
| Tetrandrine (Bisbenzylisoquinoline alkaloid) | *Stephania tetrandra* |  | ↑ROS, ↑Mitochondrial Dysfunction, ↑Cytochrome c  ↑Caspase-3, 8 &9, ↑DNA damage, ↑BAX, ↓STAT-3, ↓EGFR,↓HER2 pathway | Cervical cancer-HeLa cells. Nasopharyngeal cancer- NPC-TW 076 cells. Gastric cancer- BGC823 cells. Prostate cancer- PC-3, DU145 cells. Lung cancer- A549 cells. Breast cancer- SUM-149, SUM-159, MCF-7, MDA-MB-231, 4T1 (BALB/c mouse) cells. Brain cancer- RT-2, U87 cells. Liver cancer- Huh7, HepG2 cells & HepG2 cells male BALB/c xenograft model. Leukemia- NB4 cells. Hemangioendothelioma- EOMA cells. | (Lin, Peng, et al. 2016; Liu et al. 2011; Qin et al. 2013; Liu, Liu, and Li 2016). |

**Supplementary Table 2: Flavonoids that induce ROS mediated apoptosis in cancer.**

| **Compound** | **Plant origin** | **Structure** | **Mode of action** | **Model (Cell/Animal)** | **References** |
| --- | --- | --- | --- | --- | --- |
| Apigenin (Trihydroxyflavone) | *Petroselinum crispum, Thymus vulgaris, Origanum vulgare.* |  | ↑ROS, ↑Mitochondrial Dysfunction, ↑Cytochrome c, ↑Caspase-3, 8 &9, ↑ JNK, ↓ ERK, ↑ p38, ↓PI3K/AKT, ↓NF-κB, ↓ AR, ↑ AMPK, ↓ Wnt/β-catenin | Mesothelioma cells- MSTO-211H, H2452 cells.  Prostate cancer- 22Rv1, PC-3, DU145, PWR-1E, LNCaP cells.  Liver cancer- Hep3B and HepG2 cells.  Colon cancer- HCT-116, HT-29 cells. | (Lee et al. 2020; Shukla and Gupta 2008; Yang, Song, et al. 2021; Morrissey et al. 2005). |
| Chrysin (Dihydroxyflavone) | *Passiflora caerulea, Oroxylum indicum Passiflora incarnata,* |  | ↑ ROS, ↓ Mitochondrial Membrane potential, ↑ Cytochrome c release, ↑ER, ↑UPR ↑GRP78 ↓ AR signalling, ↓ STAT-3, ↓PI3K/AKT, ↓NF-κB, | Prostate cancer- DU 145, PC-3 cells. Bladder cancer- T-24, 5637 cells. | (Nelson et al. 2022; Xu et al. 2018; Ryu et al. 2017) |
| Eupalitin (O-methylated favonol) | *Ipomopsis*  *aggregate* |  | ↑ ROS, ↑ S phase ↑ Caspase-3 | Prostate cancer- PC-3 cells. | (Kaleem et al. 2016) |
| Juglanin (Flavonol) | *Juglans mandshurica* |  | ↑ ROS, ↑ Cell cycle arrest, ↑ JNK, ↑ caspase -3, -8, & -9. | Breast cancer cells-MCF-7, SKBR3, MDA-MB-231, BT474 & MCF-7 cells Male BALB/c nude mice xenograft model. | (Sun, Dong, and Wu 2017) |
| Auriculasin (Prenylated isoflavone) | *Flemingia*  *philippinensis* |  | ↑ ROS, ↑ Mitochondrial dysfunction, ↑ Keap1, ↑ AIFM1, ↑ Bax, ↓ Bcl-2, ↑ Cleaved PARP, ↓ AKT/mTOR/p70s6k | Prostate cancer-LnCaP cells. Colon cancer-HCT-116, SW480 cells. | (Wang et al. 2022; Nelson et al. 2022; Cho et al. 2018) |
| Licochalcone A (Chalconoid) | *Glycyrrhiza inflata* |  | ↑ ROS, ↑ DR3  ↑ DR5, ↑ Caspases-3, ↑Fas, ↑Bad, ↑Bax, ↑PUMA, ↓ PKCε, ↓p70S6K, ↓ Akt | Liver cancer- Hep G2 cells | (Wang, Zhang, et al. 2018) |
| Licochalcone D (Chalconoid) | *Glycyrrhiza*  *inflata* |  | ↑ ROS, ↓ EGFR, ↓ MET kinase, ↑G2/M cell cycle arrest, ↑ ER stress, ↑ caspase activity. | Lung cancer-HCC827, HCC827GR cells. | (Oh et al. 2020) |
| Licochalcone B (Chalconoid) | *Glycyrrhiza uralensis* |  | ↑ ROS, ↑ G2/M, ↑ Caspase 8 & 9 activity. | Lung cancer- HCC827, HCC827GR cells.  Liver cancer – HepG2 cells. | (Wang, Liao, et al. 2019; Oh et al. 2019). |
| Lupiwighteone (Isoflavone) | *Anthyllis hermanniae* |  | ↑ ROS, ↓ Cyclin B1/D1, ↓ CDK1/2/4/6, ↑ Bax  ↑ Cytochrome c  ↑ Cleaved Caspase-9  ↑ Cleaved Caspase-3  ↑ Cleaved PARP-1, ↓ P-Akt, ↑ Nrf2, ↓ Keap1 | Neuroblastoma- SH-SY5Y cells. Breast cancer- MCF-7, MDA-MB-231 cells. | (Ren, Yang, et al. 2015; Won and Seo 2020). |
| Kaempferol (Flavonol) | *Schima wallichii Korth,*  *Fragaria chiloensis* |  | ↑ ROS, ↑ cleaved PARP, ↑ p38 MAPK ↑ p53,  ↑ p21, ↑ caspase-3 | Colon cancer- HCT-116 cells. | (Choi, Kim, et al. 2018). |
| Silibinin (Flavonolignan) | *Silybum marianum* |  | ↑ ROS, ↑ p38, ↑ JNK, ↓ MEK, ↓ ERK1/2, ↓ PI3K/Akt | Fibrosarcome cells- HT-1080 cells. Breast cancer cells – MCF-7 cells. | (Duan et al. 2011; Jiang et al. 2015) |
| Quercetin (Flavonol) | *Vitis vinifera, Rubus idaeus,*  *Brassica oleracea var. italic, Solanum lycopersicum* |  | ↑ ROS, ↑ DNA damage, ↑ p53, ↑ Bax, ↑ PUMA, ↑NOXA, caspase-9, ↓ PI3K/Akt, ↓ mTOR, ↓ NF-κB, ↓ VEGF | Osteosarcoma cells- MG-63.  Glioma -U87 & U251 cells & **Male Sprague-Dawley rats C6 glioma xenograft model**.  Os, teosarcoma cells- U2-OS & U2-OS/MTX300,  Cervical cancer cells- HeLa, Breast cancer cells- MDA MB-453, 231 & MCF-7 cells BALB/c mouse Xenograft model, Leukaemia cells- U937, HL-60 cells & P39 cell NOD.CB17-PrkdC^scid^/J, lineage, Colon cancer- CT-26 BALB/c mouse Xenograft model.  Liver cancer-HepG2 cells & HepG2 cells BALB/c mouse tumor model | (Wu et al. 2020; NavaneethaKrishnan, Rosales, and Lee 2019). |
| Casticin (Tetramethoxyflavone) | *Artemisia annua, Fructus viticis, Vitex rotundifolia* |  | ↑ ROS, ↑ NOX, ↑ p38 MAPK, ↑ cytochrome c release, ↑ caspase-9, ↓ PI3K/AKT pathway | Gastric cancer- BGC-823, SGC-7901, MGC-803 cells. Colon cancer-HT-29, HCT-116, SW480 cells. Urinary bladder cancer- T24 cells. Esophageal cancer-TE-1, ECA-109 cells. Oral cancer- SCC-4 cells. Lung cancer- H460, A549, H157 cells. | (Zhou, Tian, et al. 2013; Chou, Peng, et al. 2018; Zhou, Peng, et al. 2013; Qiao et al. 2019; Chung and Kim 2016; Tang et al. 2013) |
| Sophoranone (Dihydroxyflavanone) | *Radix sophorae tonkinensis* |  | ↑ ROS, ↑ mitochondrial permeability, ↑cytochrome c release, ↑ Caspase activity. | Leukemia-U937 cells | (Kajimoto et al. 2002) |
| Fisetin (Tetrahydroxyflavone) | *Malus pumila, Diospyros species, Cucumis sativus* |  | ↑ROS, ↑NOX, ↑p38 MAPK, ↑Bax, ↑cytochrome c release, ↑caspase-9, ↓PI3K/AKT, ↑JNK | Cervical cancer- HeLa cells. Renal cancer- Caki cells. Thyroid cancer- TPC-1 cells. Oral cancer- HSC3, SCC-4 cells. Blood cancer- U266 cells. | (Afroze et al. 2022; Abotaleb et al. 2018; Min, Nam, and Kwon 2017; Su et al. 2017) |
| Isorhamnetin (3'-O-methylated metabolite of quercetin) | *Hippophae rhamnoides L., Ginkgo biloba L.*  *and Oenanthe javanica* |  | ↑ROS, ↓PCNA, ↓ cyclin A/B1, ↑p21WAF1/CIP1, ↑cytochrome c release, ↑caspase- 3, 8, & 9 | Liver cancer-Hep3B cells | (Choi 2019) |
| Nobiletin (Methoxyflavone) | *Citrus reticulate, Citrus sinesis, Citrus miaray, Citrus depressa, and Citrus tangerine.* |  | ↑ROS, DNA damage, ↑Mitochondrial dysfunction, ↑Cleaved PARP ↑gasdermin D, ↑gasdermin E | Ovarian cancer cells- A2780 and OVCAR3 | (Zhang et al. 2020) |
| Hesperetin (Flavanone glycoside) | *Citrus reticulata, Citrus limon* |  | ↑ROS, ↑NOX, ↑ p53, Bax, ↑MOMP, ↑caspase- 3, & 9 | Esophageal cancer- Eca109 cells. Breast cancer- MCF-7 cells. Gastric cancer- SGC-7901, MGC-803,  HGC-27 cells & Gastric cells male BALB/c mice xenograft model. | (Zhang et al. 2015; Wu et al. 2016; Palit et al. 2015)  ­ |
| Myricetin (Flavonol) | *Syzygium cumini, Vitis vinifera* |  | ↑ROS, ↑NOX, ↑Bax, ↑Bak, ↓SOD, p53, ↑caspase-3, & 9, ↓Bcl-2 ↓PI3K/Akt, ↑CD95, ↑TNFR1, ↑p38 MAPK, ↓NF-κB | Lung cancer- A549 cells.  Breast cancer- MDA-MB-231, MDA-MB-468, MCF-7, SK-BR-3 cells.  Colon cancer- HT-29, HCT116, SW480, SW620 cells.  Liver cancer- diethylnitrosamine (DEN), 2-acetylaminofluorene (2-AAF) induced Liver cancer in Male Sprague Dawley rats. | (Rajendran et al. 2021; Knickle et al. 2018; Zhu et al. 2020; Seydi et al. 2016) |
| Naringenin (Trihydroxyflavone) | *Vitis vinifera,*  *Citrus sinensis* |  | ↑ROS, ↑Bax, ↑Bad, ↓Bcl-2, ↓NF-κB, ↑p53, ↓PI3K/Akt, ↑p38 MAPK, ↑STAT3 | Colon cancer-RKO, DLD-1 cells. Cervical cancer- HeLa cells. Liver cancer-HepG2 cells. | (Lee, Kang, et al. 2008; Totta et al. 2004). |
| Luteolin (Tetrahydroxyflavone) | *Clerodendrum cryptophyllum* |  | ↑ROS, ↑ER stress, ↑PERK, ↑eIF2α, ↑ATF4, ↑CHOP, ↑caspase 12 | Glioblastoma- U251MG and U87MG cells. | (Wang et al. 2017) |
| Acacetin (Methylated flavone) | *Carthamus tinctorius, Scaparia dulcis,*  *Tephroseris kirilowii.* |  | ↑ROS, ↑p53, ↓Bcl-2, ↓NF-κB, ↑caspase-3&9, ↓PI3K/Akt, ↑p38 MAPK, ↓STAT3 | Leukaemia- Jurkat cells. Gastric cancer- AGS cells. Glioblastoma- U87 cells. Bone cancer- HOS, SJSA cells. Colon cancer- SW480, HCT-116 cells. | (Watanabe et al. 2012; Pan et al. 2005; Shendge, Chaudhuri, and Mandal 2021; Prasad, Sharma, and Yadav 2020; Wang, Lin, et al. 2020) |
| Eupatorin (Trimethoxyflavone) | *Lantana montevidensis, Tanacetum vulgare* |  | ↑ROS, ↑Bax ↑caspase-3, ↑JNK, ↑cytochrome c, ↑cleaved PARP | Colon cancer- HT-29, SW948 cells. Leukemia- HL-60, U937, Molt-3 cells. | (Namazi Sarvestani et al. 2018; Estévez et al. 2014). |
| Wogonin (Methylated flavone) | *Scutellaria baicalensi* |  | ↑ROS, ↑p53, ↑Bax, ↑Bak, ↑caspase-3 & 9, ↓Bcl-2, ↓PI3K/Akt, ↓STAT3 | Lung cancer- A549, A427 cells. Brain cancer- U251, U87 cells. Cervical cancer- HeLa cells. Pancreatic cancer- Panc-1, Colo-357 cells. Breast cancer- MCF-7 cells. Prostate cancer- DU145, LNCaP, 22Rv1 cells. Colon cancer- HCT-116 cells. Liver cancer- HepG2 cells. | (Wang and Cui 2019; Qian et al. 2014; Lee, Rhee, and Lee 2009; Sun, Guo, et al. 2022; Yu and Kim 2011; Li, Sun, et al. 2016; Tsai et al. 2012; He et al. 2012). |
| Baicalein (Dihydroxyflavone) | *Scutellaria baicalensis* |  | ↑ROS, ↑Bax, ↑Bak, ↓Bcl-2, ↑caspase-3 & 9, ↓NF-κB, ↓PI3K/Akt | Bladder cancer- 5637 cells. Leukemia- HL-60, CCRF-CEM cells. Bone cancer- MG-63 cells, HOS, U2OS, 143B cells. Oral cancer- Cal27 cells. Breast cancer- MDA-MB-231 cells. | (Choi et al. 2016; Pang et al. 2022; Wan and Ouyang 2018; Lee, Li, et al. 2008; Liu et al. 2019; Li, Lu, et al. 2017; Ye et al. 2015; Chen, Wu, et al. 2013; Wang et al. 2004). |
| Tangertin (Pentamethoxyflavone) | *Citrus reticulata* |  | ↑ROS, ↑p53, ↑Bax ↑cytochrome c, ↑caspase 3 & 9, ↑ cleaved PARP, ↑JNK, ↓Akt, ↓Bcl-2 | Lung cancer- H1299, H1975 cells. Bone cancer- U2OS cells. Gastric cancer- AGS cells. Colon cancer- HCT-116, KM12C, HCT-15 cells. | (Dey et al. 2020; Dong et al. 2014; Gurunathan et al. 2019). |
| Oroxylin A (O-methylated flavone) | *Scutellaria baicalensis Georgi* |  | ↑ROS, ↑cleaved PARP, ↓Akt, ↓mTOR, ↑JNK, ↑p38 MAPK, ↑Bax, ↑caspase activity, ↓Bcl-2 | Colon cancer-HCT-116, CaCo-2 cells. Brain cancer-PC-12 cells. Breast cancer- MDA-MB-231, MCF-7 cells. | (Qiao et al. 2016; Q. Han et al. 2017). |
| Galangin (Trihydroxyflavone) | *Alpinia officinarum* |  | ↑ROS, ↑JNK, ↑p53, ↓PI3K/Akt, ↑Bax, ↓Bcl-2, ↑cleaved PARP, ↑caspase-3 | Gastric cancer-MGC 803 cells.  Cervical cancer- HeLa cells. Renal cancer- 786‑0, Caki‑1 cells. | (Liang et al. 2021; Rampogu, Gajula, and Lee 2021; Cao et al. 2016). |

**Supplementary Table 3: Terpenoids that induce ROS mediated apoptosis in cancer.**

| **Compound** | **Plant origin** | **Structure** | **Mode of action** | **Model (Cell/Animal)** | **References** |
| --- | --- | --- | --- | --- | --- |
| Andrographolide (Diterpene lactone) | *Andrographis paniculata* |  | ↑ROS, ↑p53, ↑Bax, ↓Bcl-2, ↑cleavedPARP, ↑JNK, Akt/mTOR, ↓NF-κB, ↑caspase-3 | Bone cancer- HOS, U2OS, SAOS-2, MG-63.  Blood cancer- THP-1, H929 cells. Gastric cancer-SNU601, SNU638, AGS cells.  Breast cancer- MDA MB-231 cells. Colon cancer- T84, COLO 205 cells. | (Wang, Li, et al. 2020; Doi et al. 2021; Lim et al. 2017; Banerjee et al. 2016; Banerjee et al. 2017) |
| Artesunate (Sesquiterpenoid) | *Artemisia annua* |  | ↑ ROS, ↑p53, ↑JNK, ↑p38MAPK, ↑BAX, ↓Bcl-2, ↓PI3K/Akt, ↑ cleaved PARP | Breast cancer- MCF-7, T47D and MDA-MB-231.  Pancreatic cancer- BxPC-3, PANC-1 cells. Leukaemia cancer- CEM, Molt-4, Hut78, Jurkat A3, J16 cells.  Colon cancer- SW480, HCT-116 cells & CT-26 cells BALB/c Xenograft mice model. | (Efferth et al. 2007; Hamacher-Brady et al. 2011; Huang et al. 2022). |
| Costunolide (Sesquiterpene) | *Magnolia sieboldii, Laurus nobilis, Saussurea lappa* |  | ↑ ROS, ↑p53, ↑BAX, ↓Bcl-2, ↑p38 MAPK, ↑JNK ↓NF-κB, ↓PI3K/Akt, ↑ cleaved PARP | Oral Cancer- YD-10B, YD-9, Ca9-22 cells. Renal cancer- 786-O, A-498, ANCH, 769-P cells. Gastric Cancer- HGC-27, SNU-1 cells. Leukaemia- HL-60 cells. Ovarian cancer- MPSC1, A2780, SKOV3 cells. Lung cancer- A549 cells. Bladder Cancer- T24 cells. Prostate cancer- PC-3, DU-145 cells. Colon cancer- HCT-116 cells. | (Huang et al. 2021; Z. Wang, Zhao, and Gong 2016; Zhuge et al. 2018; Fu et al. 2020; Xu et al. 2021; Rasul, Bao, et al. 2013; Lee et al. 2001; Chen et al. 2017; Yang et al. 2011)  ­ |
| Saxifragifolin  (Triterpenoid saponins) | *Androsace umbellata* | **** | ↑p38 MAPK, ↑Bax, ↓Bcl-2, ↑caspase-3, ↑cleaved PARP | Breast cancer-MCF-7, MDA-MB-231. | (Shi et al. 2013) |
| Celastrol (Pentacyclic triterpenoid) | *Tripterygium wilfordii, Tripterygium regelii* |  | ↑ ROS, ↑p53, ↑BAX, ↓Bcl-2, ↑p38 MAPK, ↑JNK ↓NF-κB, ↓PI3K/Akt, ↑ cleaved PARP, ↑ caspase-3 | Gastric cancer- SGC-7901, BGC-823 cells. Lung cancer-H460, PC-9, H520 cells. Bone cancer- HOS, MG-63, U-2OS, Saos-2 cells & HOS cells female BALB/c nude mouse xenograft model. Ovarian cancer- SKOV3 cells. | (Chen et al. 2020; Zhao, Wang, et al. 2022; Niu et al. 2020; Li, Zhang, et al. 2015) |
| Carvacrol | *Citrus aurantium, Origanum vulgare, Thymus vulgaris, Lepidium favum* |  | ↑ROS, ↑p38 MAPK, ↑JNK, ↓PI3K/Akt, ↑Bax. ↓Bcl-2, ↑ caspase activity. | Prostate cancer- DU145, PC-3 cells. Lung cancer- A549, H460, H1299 cells. Choriocarcinoma- JAR, JEG3 cells. | (Khan et al. 2019; Khan et al. 2017; Sampaio et al. 2021; Lim et al. 2019; Khan et al. 2018) |
| Oleanolic acid (Pentacyclic triterpenoid) | *Vitis vinifera* |  | ↑ROS, ↑p53, ↓PI3K/Akt/mTOR, ↑JNK, ↑Bax, ↓Bcl-2, ↑ cleaved PARP, ↑ caspase-3 | Pancreatic cancer-Panc-28 cells. Breast cancer- MCF-7 cells. Liver cancer- HepG2 cells & HepG2 cells male BALB/c mice xenograft model. | (Wei et al. 2013; Chu et al. 2017) (Wang et al. 2013; Tang et al. 2022) |
| Tanshinone (norditerpenoid quinone) | *Salvia miltiorrhiza* |  | ↑ROS, ↑ cell cycle arrest, ↑p21, ↑ER stress, ↓DRAM | Liver cancer- HepG2, Huh7 cells. | (Cheng et al. 2013) |
| Helenalin (sesquiterpene lactone) | *Arnica montana, Arnica chamissonis* |  | ↑ROS, ↑ER, ↑REDD-1, ↑ATF4, ↑CHOP, ↑JNK ↓NF-κB, | Renal cancer- Caki, ACHN cells. Colon cancer- HCT-116, HT-29 cells. Breast cancer- MCF-7. Pancreatic cancer- L3.6pl cells. Prostate cancer- DU145, PC-3 cells. | (Jang et al. 2013; Hoffmann et al. 2011; Yang, Zhang, et al. 2021) |
| Jungermannenone (ent-Kaurane Diterpenoid) | *Jungermannia fauriana* |  | ↑ROS, ↑mitochondrial damage, ↑DNA damage, ↓c-Myc | Lung cancer-A549 cells. Leukaemia cancer- U937, NB4 cells. Prostate cancer- PC-3 cells. | (Sun et al. 2021; Yue et al. 2018; Guo et al. 2016) |
| Linalool | *Cinnamomum osmophloeum,*  *Lavandula angustifolia* |  | ↑ROS, ↓Mitochondrial trans membrane potential, ↑cell cycle arrest | Human oral carcinoma cells- KB cells | (Pushpalatha et al. 2021) |
| Oridonin | *Isodon Rubescens* |  | ↑ROS, ↓gamma-glutamyl transpeptidase 1 (GGT1), ↓glutathione peroxidase 4 (GPX4) | Esophageal cancer cells-TE1 cells | (Zhang, Wang, et al. 2021) |
| Cucurbitacin B | *Helicteres angustifolia, Licaniaintra petiolaris, Casearia arborea, and Cucumis prophetarum* |  | ↑ROS, ↑γH2AX, ↑DNA damage, ↑caspase-8, -9 and -3, ↓cyclin D1, ↓cyclin-dependent kinase 4 (CDK4), ↑JAK, ↓STAT-3 | Human cervical cancer- HeLa cells and Breast cancer cells- MCF-7 cells | (Zhang et al. 2012; Ren, Sha, et al. 2015) |
| Carnosol | *Rosmarinus officinalis, Origanum vulgare* |  | ↑ROS, ↑DNA damage, ↓Mitochondrial potential ↑cell cycle arrest, ↑p21/WAF1, ↑ beclin1, ↓p27, ↑pERK1/2 | Breast cancer cells-MDA MB-231 | (Dhaheri et al. 2014) |
| Isoalantolactone | *Inula helenium* L |  | ↑ROS, ↑DNA Damage, ↑ER stress, ↓p-STAT3, ↓STAT3, ↑p38 MAPK, ↑Bax, ↑cleaved caspase-3, ↓Bcl-2, ↓ SIRT1, | Prostate cancer- LNCaP, PC-3, DU145 cells.  Pancreatic cancer- PANC-1 cells. Colon cancer- HCT-116, HCT-15 cells. Breast cancer- MDA-MB-231, MCF-7 cells | (Chen, Li, et al. 2018; Khan, Ding, et al. 2012; Wu, Shao, et al. 2022; Li, Qin, et al. 2016; Rasul, Di, et al. 2013) |
| Alantolactone | *Inula helenium* |  | ↑ROS, ↑Mitochondrial dysfunction ↑Cell cycle arrest, ↓Bcl-2, ↑Bax, ↑cleaved PARP, ↑CDK1/1B, ↓cyclin D1, ↓RAC-alpha serine/threonine-protein kinase (AKT), ↓ PINK1, ↓SIRT1 | Hepatocarcinoma- HepG2 cells. Gastric cancer-BGC 823 cells. Glioblastoma-U87, U373, LN229 cells. | (Kang et al. 2019; Zhang and Zhang 2019; Khan, Yi, et al. 2012) |
| Escin (Triterpene saponins) | *Aesculus hippocastanum* |  | ↑ROS, ↑ Mitochondrial membrane potential dysfunction, ↑cell cycle arrest, ↑caspase-3&9, ↑cleaved PARP, ↑Bax, ↓Bcl-2, ↑p38 MAPK, ↓ERK | Bone Cancer-HOS, Saos-2, MG-63, U2-OS. Ovarian cancer- A2780 cells. Renal cancer- 786-O, Caki-1 cells. | (Zhu et al. 2017; Wei et al. 2022; Yuan et al. 2017) |
| Parthenolide | *Tanacetum parthenium* |  | ↑ROS, ↓glutathione, ↓Mitochondrial membrane potential, ↑DNA damage, ↑caspases-7, -8, &-9, ↑GADD153, | Prostate cancer- PC-3 cells. Bone cancer- Saos-2, MG-63 cells. Leukaemia- Meg‐01, K562, SEM, RS4;11, REH, Kasumi‐4, KCL‐22 cells. Breast cancer- MDA MB-231 cells & MDA-MB231 cells female nude athymic mice xenograft model. Liver cancer-Hep 3B, SH-J1 cells. | (Nelson et al. 2022; Flores-Lopez et al. 2018; Wen et al. 2002; Zunino, Ducore, and Storms 2007) |
| Pristimerin (quinonemethide triterpenoid) | *Maytenus ilicifolia* |  | ↑ROS, ↑Mitochondrial dysfunction, ↑Bax, ↓Bcl-2, ↑ cytochrome C release, , ↑ cycle arrest, ↑ caspase-3, ↑ASK1, ↑ JNK, ↑UPR, ↓EGFR | Breast cancer-MDA-MB-231, MDA-MB-468 cells & MDA-MB-231 cells female BALB/c nude mice xenograft model. Skin cancer- A431, A388 cells. Glioblastoma-U87 cells. Liver cancer- HepG2 cells. Colon cancer- HCT-116 cells & HCT-116 cells female BALB/c nude mice. Prostate cancer- LNCaP, PC-3 cells. | (Zhao et al. 2019; Al-Tamimi et al. 2022; Yousef et al. 2016; Liu et al. 2013; Guo et al. 2013; Yan et al. 2013) |
| Bigelovin (Sesquiterpene lactone) | *Inula helianthus aquatic* |  | ↑ROS, ↑caspase-3, ↑cell cycle arrest, ↑DNA damage, ↑cleaved PARP, ↑ Beclin-1, ↓ AKT, ↓mTOR, ↑DR-5 | Colon cancer-HCT-116, HT-29 cells. Liver cancer- HepG2 cells. | (Vallejo, Salazar, and Grijalva 2017; Li, Song, et al. 2017; Wang, Zhou, et al. 2018) |
| Germacrone (Monocyclic sesquiterpene) | *Curcuma zedoaria, Curcuma phaeocaulis* |  | ↑ROS, ↑Mitochondrial dysfunction, ↑ caspase 3, ↑GSDME | Liver cancer-HepG2 cells. Breast cancer-  MCF-7, MDA-MB-231 cells. | (Sun, Zhong, et al. 2022; Chen et al. 2011) |
| Furanodienone (Sesquiterpene) | *Rhizoma Curcumae* |  | ↑ROS, ↑p-p38, ↑MAPK, ↑p-JNK, ↓p-ERK, ↑caspase-3, 8 & 9 | Colon cancer- RKO, HT-29 cells. | (Jiang, Wang, and Hu 2017) |
| Deoxyelephantopin (Sesquiterpene lactone) | *Elephantopus scaber* |  | ↑ROS, ↓glutathione, ↓thioredoxin reductase, ↓Mitochondrial dysfunction, ↓Bcl-2, cytochrome c release, ↑caspases-3, ↑cleaved PARP, ↓NF-κB | Liver cancer- HepG2 cells.  Bone cancer- MG-63, U2OS, Saos2 cells. | (Zou et al. 2017; Mehmood et al. 2017) |

**Supplementary Table 4: Polyphenolic compounds that induce ROS mediated apoptosis in cancer.**

| **Compound** | **Plant origin** | **Structure** | **Mode of action** | **Model (Cell/Animal)** | **References** |
| --- | --- | --- | --- | --- | --- |
| Curcumin | *Curcuma longa* |  | ↑ROS, ↑ER stress, ↓Mitochondrial membrane potential, ↑Caspase activity | Colon cancer cells-HCT-116, HT-29. KM12C cells. Liver cancer cells-HepG2, Hep-3B. Kidney cancer cells- Caki-1. Prostate cancer-LNCaP & PC-3 cells BALB/c xenograft model. | (Khaket et al. 2020; Termini et al. 2020) |
| Tannic acid | *Caesalpinia spinosa, Rhus semialata, Quercus infectoria* |  | ↑ROS, ↑ER stress, ↑DNA fragmentation, activity, ↓Wnt/β-catenin, ↑TRAIL, ↑caspase activity | Prostate cancer- C4-2, PC-3 cells. Embryonic carcinoma- EC; NCCIT cells. Liver cancer- HepG2 cells. | (Nagesh et al. 2020; Mhlanga et al. 2019; Sp et al. 2020). |
| Zingerone | *Zingiber officinale* |  | ↑ROS, ↑Mitochondrial dysfunction, ↓ Bcl-2, ↑Bax, ↑caspase-3 & 9 | Colon cancer- HCT-116 cells. | (Su et al. 2019) |
| Eugenol | *Syzygium aromaticum, Eugenia caryophyllata* |  | ↑ROS, ↑ p53, ↓Mitochondria membrane potential, ↑cytochrome c release, ↓ Bcl-2, ↓ glutathione, ↑ DNA fragmentation, ↑ cleaved PARP, ↑caspase-3 | Leukemia- HL-60, U937 cells. Colon cancer- HCT-15, HT-29 cells. Breast cancer-MCF-7 cells. | (Jaganathan et al. 2011; Yoo et al. 2005; Vidhya and Devaraj 2011) |
| Carnosic acid (Phenolic diterpene) | *Rosmarinus officinalis* |  | ↑ROS, ↑p53, ↑Bax, ↓Mdm2, ↓Bcl-2, ↓Bcl-xl, ↑caspase-3 & 9, ↑cleaved PARP, ↓cyclin D1, ↓survivin, ↓ STAT-3 | Colon cancer- HCT-116 cells. | (Kim et al. 2016) |
| Epicatechin | *Camellia sinensis* |  | ↑ROS, ↑DNA fragmentation, ↑DR4&DR5, ↑Bad, ↑Bax | Breast cancer-MDA-MB-231, MCF-7 cells | (Pereyra-Vergara et al. 2020) |
| Epigallocatechin-3-gallate | Green tea *Camellia sinensis* |  | ↑ROS, ↓Mitochondria membrane potential, ↑caspase-3, ↑Bax, ↓Bcl-2 ↓PI3K, ↓Akt | Leukaemia cells- UF-1 & UF-1 cells SCID mouse model, B-Lynphoblastoid cells- Ramos (RA-1), Bladder cancer- T24 cells, Liver cancer- SMMC7721 cells, Pancreatic cancer-PANC-1 cells, Lung cancer- H1299 cells. | (NavaneethaKrishnan, Rosales, and Lee 2019; Gu et al. 2018; Li et al. 2009) |
| Resveratrol | *Vitis vinifera, Polygonum cuspidatum* |  | ↑ROS, ↑p53, ↑cell cycle arrest, ↓p21, ↑p27, ↑cyclin E, ↓Bcl-2, ↑p38 MAPK, ↑ER stress, ↑ eIF2, ↓Notch1, ↑PTEN, ↓Akt | Prostate cancer- LNCaP, PC-3 and DU-145. Brain cancer- Neuro 2A cells & Neuro 2A cells Syngeneic A/J mice. Ovarian cancer- A2780, SKOv3 cells. Skin cancer-A375SM cells. Leukaemia cells- MOLT-4, HL-60 cells. Colon cancer- HCT-116, SW620 cells. Anaplastic thyroid carcinoma-THJ-16T, THJ-11T cells. | (Chen et al. 2004; Kim, Park, and Woo 2019; Heo et al. 2018) |
| Gallic acid | *Camellia sinensis, Cynomorium coccineum, Myriophyllum spicatum* |  | ↑ROS, ↑ p53, ↑Mitochondrial dysfunction, ↑cytochrome c release, ↑caspases 3, 8 & 9, ↑p38MAPK, ↑JNK, ↓ERK, ↓Akt | Prostate cancer- LNCaP cells.  Lung cancer- NCI-H460 cells & NCI-H460 cells xenograft mouse model | (Ji et al. 2009; Russell et al. 2012; Chen, Chen, et al. 2013)- |
| Rosmarinic acid | *Salvia officinalis* |  | ↑ROS, ↑cycle arrest, ↓NF-κB, ↓p-Akt, ↓p-ERK, ↓VEGF, ↓MMP-2, ↓MMP-9, ↓NLRP3 | Bone cancer- U2OS, MG63 cells. | (Chaitanya et al. 2022) |
| Hispidulin (Phenolic flavonoid) | *Saussurea involucrata* |  | ↑ROS, ↑ER stress, ↓Mitochondrial membrane integrity, ↓Bcl-2, ↑Bax, ↑cytochrome C release, ↑cleaved caspase‑3, cleaved PARP, capase-3, ↓P13k/Akt. | Lung cancer-NCI-H460, A549 cells & NCI-H460 cells male athymic BALB/c mouse xenograft model. Liver cancer-HepG2 cells | (Lv et al. 2020; Gao, Wang, and Peng 2014) |
| Sparstolonin B | *Sparganium stoloniferum* |  | ↑ROS, ↓glutathione, ↑cell cycle arrest, ↓PI3K, ↓Akt, ↑cleaved caspase-3 | Prostate cancer- DU-145, PC-3 & PC-3 cells male BALB/c xenograft model. Brain tumor- SK-N-BE(2), NGP, IMR-32, SH-SY5Y, SKNF-1 cells. | (Liu et al. 2021; Kumar et al. 2014) |
| Caffeic acid (hydroxycinnamic acid) | *Salvia miltiorrhiza* |  | ↑ROS, ↑p53, ↑DNA fragmentation, ↓uncleaved caspase-3, ↓Bcl-2 | Cervical cancer-HTB-34 cells. | (Choi, Tran, et al. 2018) |

**Supplementary Table 5: Glycosides that induce ROS mediated apoptosis in cancer.**

| **Compound** | **Plant origin** | **Structure** | **Mode of action** | **Model (Cell/Animal)** | **References** |
| --- | --- | --- | --- | --- | --- |
| Ouabain (Cardiac glycosides) | *Acokanthera schimperi, Strophanthus gratus*. |  | ↑ROS, ↑ ER-stress, ↑Ca^+2^, ↓Mitochondrial membrane potential, ↑ caspase-3, 8, &9, ↑DR4, ↑DR5, ↑FADD, ↑ TRAIL | Prostate cancer- DU145 cells. Bone cancer-U2OS Tercells. | (Chang et al. 2019; Chou, Liu, et al. 2018) |
| Atractyloside  (Perhydrophenanthrenic glycoside) | *Atractylis gummifera* |  | ↑ROS, ↑ER stress, ↑Ca^+2^, ↑Mitochondrial dysfunction | Leukaemia- HL-60 cells. | (Zhang et al. 2006) |
| Icariin (Flavonol glycoside) | *Epimedium koreanum,*  *Epimedium brevicornum.* |  | ↑ ROS, ↑ER stress, ↓glutathione (GSH), ↑caspase 9, ↑p-PERK, ↑GRP78, ↑ATF4, ↑p-eIF2α, ↑CHOP, ↑ PUMA, ↓Bcl-2 | Breast cancer-MDA‐MB‐231, MDA‐MB‐453, MCF-7/TAM (tamoxifen resistant cell) 4T1 (Mouse breast cancer). Colon cancer-HCT116 cells. Esophageal cancer- EC109, TE1 cells. | (Song, Chen, et al. 2020; Fan et al. 2016; Kim et al. 2020) |
| Gypenosides (Dammarane type- triterpene glycosides) | *Gynostemma pentaphyllum* |  | ↑ ROS, ↑Ca^+2^, ↑Mitochondrial dysfunction, ↑Cytochrome c release, ↑DNA damage, ↑Bax, ↓Bcl-2, ↑ER stress, ↑GADD153, ↑GRP78, ↑ATF6-α, ↑ATF4-α | Colon cancer-Colo-205, SW-480, Caco-2, SW-480, SW620 cells & female CT-26 cells BALB/c mice xenograft model. Esophageal cancer- Eca 109 cells. Leukemic Cancer- HL-60, WEHI-3 cells & WEHI-3 cells Male BALB/c mice xenograft model, HL-60 cells female athymic nude mice xenograft model. Oral cancer- SCC-4 & SSC-4 cells male athymic BALB/c nude mouse xenograft model. Liver cancer- Huh-7 cells. Bladder cancer- T24, 5637 cells. | (Kong et al. 2015; Chen et al. 2006; Wang et al. 2007; Li et al. 2022; Chen et al. 2009; Yan, Wang, Niu, et al. 2014; Yan, Wang, Wang, et al. 2014; Hsu et al. 2011) |

**Supplementary Table 6: Other compounds that induce ROS mediated apoptosis in cancer.**

| **Compound** | **Plant origin** | **Structure** | **Mode of action** | **Model (Cell/Animal)** | **References** |
| --- | --- | --- | --- | --- | --- |
| Altholactone (Styryl lactone derivative) | *Goniothalamus species* |  | ↑ROS, ↑BAX, ↓Bcl-2, ↑MAPK-p38, ↓PI3K, ↓Akt, ↓STAT3 ↓p65, ↓TNF-α, ↓IL-6, ↓NF-κB | Prostate cancer- DU-145 cells. Bladder cancer- T24 cells | (Jiang et al. 2017; Zhao and Li 2014) |
| Acetylshikonin (naphthoquinone pigment) | *Lithospermum erythrorhizon, Arnebia species* |  | ↑ROS, ↑DNA damage, ↑cell cycle arrest, ↑γH2AX, ↑PARP, ↑FOXO3, ↑Bax, ↑Bad, ↑p21, ↑p27, ↑caspase-3, 6, 7, 8 & 9 | Colon cancer- HCT-15, LoVo cells. Renal cancer- A498, ACHN cells. | (Lim et al. 2021; Lim et al. 2022) |
| Annomuricin E (Fatty acid) | *Annona muricata* |  | ↑ROS, ↑Cell cycle arrest, ↑cytochrome c release, ↓MMP, ↑ Bax ↓ Bcl-2, ↑caspase actvity | Colon cancer- HT-29, HCT-116 cells | (Ilango et al. 2022) |
| Chikusetsu (Saponin) | *Aralia taibaiensis* |  | ↑ ROS, ↓Mitochondrial dysfunction, ↑Cytochrome c release, ↑Endo G, ↑caspase activity | Prostate cancer- PC-3, LNCaP cells. | (Zhu, Tian, and Liu 2017) |
| Sulforaphane  (isothiocyanate) | *Brassica species* |  | ↑ROS, ↓GSH, ↓Bcl-xL, ↑Bid↑Fas, ↑caspase-8 | Prostate cancer cells- PC-3 and DU145. Pancreatic cells- MIA PaCa-2, Panc-1. | (Singh et al. 2005) |
| Benzyl Isothiocyanate (isothiocyanate) | *Brassica species* |  | ↑ROS, ↓GSH, ↓ c-Myc, ↓STAT-3, p-STAT-3 | Pancreatic cancer- Capan-2, MIAPaCa-2, L3.6pL cells. Brain cancer- U87MG cells. Prostate cancer- CRW-22Rv1, PC3. | (Sahu et al. 2009; Kasiappan et al. 2016) |
| Deoxypodophyllotoxin (lignan) | *Juniperus communis* |  | ↑ROS, ↓EGFR, ↓MET, ↓pEGFR, ↓pMET, ↓pErbB3, ↓pAKT, and pERK, ↑MAPK, ↓ERK, ↓NFκB | Breast cancer- MDA MB-231 cells. Lung cancer- HCC827GR cells. | (Benzina et al. 2015; Kim et al. 2021) |
| Phenethyl Isothiocyanate  (isothiocyanate) | *Brassica species* |  | ↑ROS, ↑Ca^+2^, ↓Mitochondrial membrane potential, ↓Bcl-2, ↑BAX, ↑caspase-3, 8, &9 | Breast cancer cells- MDA-MB-231, MCF-7 cells.  Ovarian cancer cells- T72Ras, SKOV-3, HEY cells. Lung cancer- A549 cells & A549 cells BALB/c female mouse xenograft model. Prostate cancer- LNCaP, PC-3 cells. Leukaemia- K-562 cells | (Gupta et al. 2014) |
| Purpurin (Anthraquinone) | *Rubia tinctorum* |  | ↑ROS, ↑ phospholysine phosphohistidine inorganic pyrophosphate phosphatase (LHPP), ↓PI3K/AKT, ↓EGFR, ↓cyclin-D1, ↓PCNA, ↑Bax, ↑caspase-3&9 | Lung cancer- A549 cells. Colon cancer- HCT-116 cells. | (Bo et al. 2021; Li et al. 2021) |
| Honokiol (Bisphenol lignin) | *Magnolia officinalis* |  | ↑ROS, ↑p53 ↓Membrane potential, ↑cell cycle arrest, ↑BAX, ↓Bcl-2, ↓PI3K, ↓Akt, ↓mTOR, ↓ERK1/2, ↑caspase-3&7, | Liver cancer-SMMC-7721 cells. Brain cancer- U87 MG cells. Bone cancer- HOS, U2OS cells & HOS cells Female BALB/c mouse xenograft model. Bladder cancer- BFTC‑905 cells. | (Han et al. 2009; Hsiao, Yao, et al. 2019; Huang, Chen, et al. 2018; Lin, Chen, et al. 2016)  ­ |
| Delphinidin (Anthocyanidin) | *Hibiscus sabdariffa, Solanum melongena* |  | ↑ROS, ↑Mitochondrial membrane potential, ↑DNA fragmentation, ↑Cytochrome c release, ↑cleaved PARP, ↑ JAK, ↓STAT3, ↓mTOR, ↑AMPK, ↑caspase-3, ↓NF-κB/p65, ↓Bcl2, ↓Ki67, ↓PCNA | Bone cancer- U2OS cells.  Bladder cancer- T24 cells. Breast cancer- MDA-MB-453, BT474 cells. Colon cancer- HCT-116 cells. Leukemia- HL-60 cells. | (Lee et al. 2018; Zhang, Pan, et al. 2021; Chen, Zhu, et al. 2018; Hou et al. 2005; Kang et al. 2021) |
| Genistein (Phytosterol) | *Glycine max* |  | ↑ROS, ↑Ca^2+^, ↓Mitochondrial membrane potential, ↑cleaved PARP, ↑Bax, ↑Bad, ↑Bak ↑ER stress, ↑IRE-1α, ↑Calpain 1, ↑GRP78, ↑GADD153, ↑caspase-3, 4, 7 & 9, ↑ATF-6α, | Leukemia-HL-60 cells & HL-60 cells male athymic BALB/c xenograft model. Liver cancer- HepG2 cells. | (Hsiao, Peng, et al. 2019; Zhang, Bao, and Yang 2019) |
| Daidzein (Phytoestrogen) | *Pueraria lobata, Glycine max, Psoralea corylifolia.* |  | ↑ROS, ↓Mitochondrial membrane potential, ↑cycle arrest, ↓Bcl-2, ↓Bcl-x, ↓Baid, ↑BAX, ↑JNK, ↓EGFR, ↓STAT-3, ↓AKT, ↓ERK, ↑caspase-7 & 9, ↓ER α/β ratio | Liver cancer-BEL-7402 cells. Breast cancer- MCF-7 cells. Cervical cancer- HeLa cells. Lung cancer- A549 cells. | (Kaufman et al. 1997; Mhone et al. 2022; Jin et al. 2010; Kumar and Chauhan 2021; Yao, Xu, and Huang 2021; Han et al. 2015) |
| Plumbagin (naphthoquinone drivative) | *Plumbago zeylanica* |  | ↑ROS, ↑ER stress, ↑cycle arrest, ↓cyclin B1, A & E2, ↓CDK 1, ↑Bax/Bcl2 ratio, ↑ caspase 3 & 9, ↑cleaved PARP, ↓1, 4-phopshatidylinositol 5-kinase (PI5K)-1B, | Cervical cancer- ME-180, SiHa, HeLa cells.  Prostate cancer-DU145, PC-3. Pancreatic cancer- PANC-1, MIA PaCa-2 cells. Leukaemia- NB4 cells & NB4 cells NOD/SCID mice xenograft model. Skin cancer-A431 cells. Breast cancer- MCF-7 cells. Canine cancer- CTAC, Denny, Payton, 17CM cells. | (Srinivas et al. 2004; Jaiswal et al. 2018; Huang, Xie, et al. 2018; Pandey, Tripathi, et al. 2020; Xu and Lu 2010; Nazeem et al. 2009; Lee et al. 2012; Alharbi et al. 2019). |
| Picropodophyllotoxin (lignin, epimer of podophyllotoxin) | *Podophyllum peltatum, Podophyllum hexandrum* |  | ↑ROS, ↑Mitochondrial dysfunction, ↑ cell cycle arrest, ↑ER stress, ↑GRP78, ↑CHOP, ↑DR5, ↑DR4 ↓EGFR, ↓MET, ↑p38 MAPK ↓AKT, ↓ERK | Colon cancer-HCT116 cell.  Esophageal cancer- KYSE 30, KYSE 70, KYSE 410, KYSE 450, KYSE 510 cells.  Lung cancer-HCC827GR cells | (Lee et al. 2021; Kwak et al. 2020; Lee et al. 2022) |
| Vitexin (Lignan) |  |  | ↑ROS, ↑cleaved caspase-3 & 9, ↑BID, ↑Bax. | Breast cancer- MDA-MB‑231 cells. Colon cancer- HCT-116 cells. Skin cancer- A375, Sk-Mel-5, Sk-Mel-28 cells | (Liu et al. 2014; Bhardwaj et al. 2018) |
| Α-Lipoic Acid  (Dithiol or Pentanoic acid) | *Solanum lycopersicum L,*  *Brassica species* |  | ↑ ROS, ↑p53, ↑ ER stress, ↑UPR, ↑JNK, ↑GADD153, ↑CHOP, ↑PERK, ↑IRE1, ↑ATF6 | Liver cancer-HepG2 cells | (Pibiri et al. 2020) |
| Oblongifolin C (polyprenylated benzoylphloroglucinol natural products) | [*Garcinia*](https://www.sciencedirect.com/topics/medicine-and-dentistry/garcinia) yunnanensis |  | ↑ROS, ↑cleaved casapse-3, ↑ cleaved PARP, ↑p-JNK | Colon cancer- HCT-116 cells | (Li, Meng, et al. 2017) |
| Guttiferone- K (polyisoprenylated benzophenones) | [*Garcinia*](https://www.sciencedirect.com/topics/medicine-and-dentistry/garcinia) yunnanensis |  | ↑ROS, ↑cleaved casapse-3, ↑cleaved PARP, ↑JNK, ↓ p62, ↓mTOR, ↓Akt | Colon cancer- HCT-116 cells.  Cervical cancer-HeLa cells. | (Li, Meng, et al. 2017; Wu et al. 2015) |
| Withaferin A (Steroidal lactone) | *Withania somnifera* |  | ↑ROS, ↑Nrf2, ↑KEAP1, ↑HO-1, ↓glutathione peroxidase 4 (gpx4), | Neuroblastoma cells-IMR-32, SK-N-SH, NB69, CHP-134 and SH-SY5Y cells | (Hassannia et al. 2018) |
| Bromelain  (Proteolytic enzyme) | *Ananas comosus* |  | ↑ROS, ↓ACSL-4, ↓EGFR, ↓MYC, ↓PCNA ↓ STAT1, ↑miR-223-3p | Colorectal cancer cells-HCT-116, DLD-1, CacO_2_ and NCI-H508 cells | (Park et al. 2018) |
| Ruscogenin (Steroidal sapogenin) | *Ruscus aculeatus* |  | ↑ROS, ↑transferrin, ↓ferroportin | Pancreatic cancer cells- BxPC-3, SW1990, PANC-1, and AsPC-1 cells | (Song, Xiang, et al. 2020) |
| Allicin (Organosulfur compound) | *Allium sativum* |  | ↑ROS, ↑p53, ↑Caspase activity, ↑MAPK, ↑JNK, ↓HIF-1α, ↓HIF-2α | Lung cancer cells- A549, NCI-H460 and Liver cancer cells- HepG2 | (Chu et al. 2013; Pandey, Tyagi, et al. 2020) |
| Emodin (Anthraquinone derivative) | *Rheum palmatum and Aloe vera* |  | ↑ROS, ↓ RhoA, ↑caspase-3 | Gastric cancer cells-SGC-7901 | (Cai et al. 2008) |
| Psoralidin (Coumarin derivative) | *Psoralea corylifolia* |  | ↑ROS, ↓Mitochondrial membrane potential, ↑DNA damage, ↑caspase-3&7, ↑c-JNK-1/2 | Colon cancer- HT-29, HCT-116. | (Sun, Zhao, et al. 2022) |
| p-Coumaric acid (Phenolic acid) | *Arachis hypogaea, Daucus carota, Solanum lycopersicum* |  | ↑ROS, ↑Cell cycle arrest | Colon cancer-HT-29, HCT-15 cells. | (Rosa et al. 2018) |
| 2-methoxy-1,4-naphthoquinone  (Quinone derivative) | *Impatiens balsamina, Impatiens glandulifera* |  | ↑ROS, ↑DNA damage, ↑JNK, ↑p38 MAPK | Lung cancer- A549 cells. | (Ong et al. 2015) |
| 8-Hydroxy-2-methoxy-1,4-naphthoquinone  (Quinone derivative) | *Juglans sinensis* |  | ↑ROS, ↑cleaved PARP-1 cleavage, ↑Bax, ↓Bcl-2, ↑JNK | Lung cancer- A549 cells, Breast cancer- MCF-7. Colon cancer-HCT-116. | (Lee et al. 2017) |
| Isobavachalcone (Chalcone derivative) | *Psoralea corylifolia* |  | ↑ROS, ↑ER stress, ↑Mitochondrial dysfunction, ↑Bax/Bcl-2 ratio, ↑cleaved caspases-3, ↑RIP3, ↑p-RIP3, ↑MLKL, ↓Akt, ↓p-Akt-473, ↓ TrxR1, | Breast cancer- MDA-MB-231, MCF-7 cells. Prostate cancer- PC-3 cells. | (Wu, Gao, et al. 2022; Li et al. 2018; Zhang, Gao, et al. 2022) |
| Fucoxanthin  (Xanthophyll carotenoid) | *Sargassum siliquastrum* |  | ↑ ROS, ↓Mitochondrial membrane potential, ↓CAT, ↓SOD, ↓GSH | Oral cancer- KB cells | (Heo and Jeon 2009; Iyappan et al. 2021) |
| Shikonin (Naphthoquinone derivative) | *Lithospermum*  *erythrorhizon* |  | ↑ ROS, ↑ER stress, ↓Mitochondrial membrane, PARP, ↑γH2AX, ↑FOXO3, ↑Bax, ↑Bim, ↑Bad, ↑p21, ↑p27 ↑JNK, ↑p38, ↑caspase-3, -8 & 9, ↓pAkt, ↓Bcl-2, ↓Bcl-xL, ↓peroxiredoxin, ↓thioredoxin 1, ↑PERK, ↑elF2α, ↑ATF4, ↑CHOP, ↑IRE1α | Brain cancer- U87MG, Hs683 cells.  Prostate cancer-PC-3, DU145 cells. Gastric cancer- BGC-823, SGC-7901 cells & SGC-7901 cells BALB/c nude mice. Colon cancer- HCT116, HCT-15, SW480 cells & SW480 cells male BALB/c nude mice xenograft model, HCT116 and HCT-15 cell BALB/c nude mice xenograft model. non-Hodgkin's lymphoma- BCBL-1, BC-1, BC-3, TY-1, GTO cells & GTO cells Nude Rag2/Jak3 mice xenograft model. Renal cancer- Caki-1 and ACHN cells. Bile duct cancer- RBE cells. Skin cancer- A375 cells. | (Yang et al. 2014; Nelson et al. 2022; Liang et al. 2016; Zhou et al. 2017; Qi et al. 2022; Alam et al. 2021; Tsai et al. 2021) |
| Gambogic acid (Xanthonoid) | *Garcinia hanburyi tree* |  | ↑ROS, ↓Mitochondria membrane potential, ↑cell cycle arrest, ↑p53, ↑BAX, ↑p66shc, ↑FOXO3a, ↑ Nrf2, ↑p27Kip1, ↑JNK, ↓SIRT1, ↑caspase-3 & 9 | Liver cancer- SMMC-7721 cells. Esophageal Cancer- EC9706 cells. Breast cancer- MCF-7 cells. bladder cancer- EJ cells. Pancreatic cancer- PANC-1, BxPC-3 cells. Skin cancer- A375 cells. | (Nie et al. 2009; Liang and Zhang 2016; Zhen et al. 2015; Wang, Zhao, et al. 2019; Lyu et al. 2018; Liu, Fan, et al. 2022) |
| Guggulsterone (Phytosterol) | *Commiphora mukul* |  | ↑ROS, ↑JNK, ↑ER stress, ↑CHOP, ↑caspase3, 8&9, ↑DR5 | Prostate cancer- LNCaP, PC-3 cells. Bile duct cancer- HuCC-T1, RBE cells. | (Nelson et al. 2022; Zhong et al. 2016) |

**Supplementary Table 7: Alkaloids induce ROS mediated apoptosis in colon cancer.**

| **Class** | **Phytocompounds** | **Biological source** | **structure** | **Combination (In colon cancer)** | **Cancer model (Cell/Animal)** | **Molecular Targets /Information** | **Reference** |
| --- | --- | --- | --- | --- | --- | --- | --- |
| **Isoquinoline Alkaloids** | Sanguinarine (Benzophenanthridine alkaloid) | *Macleaya cordata*, |  | Nil | SW480, HCT116 | ↑ROS, DNA damage, ↑cytochrome c, ↑ATP, ↑Bax, ↓association of STRAP and MELK, ↑caspase- 3, ↑Apoptosis. | (Gong et al. 2018) |
|  | 6-Methoxydihydrosanguinarine (Derivative of Sanguinarine) | Derivative of Sanguinarine |  | Nil | Various cancer cells | ↑ROS, ↑DNA damage, ↑Apoptosis | (Yin et al. 2005) |
|  | Berberine (Benzylisoquinoline alkaloid) | *Berberis genus* |  | Nil | HT-29, HCT-116 | ↑ROS, ↑ interaction of lncRNA CASC2 with AUF1, ↓ Bcl-2, ↑Apoptosis | (Dai et al. 2019) |
|  |  |  |  | NIl | IMCE cells with Apc min mutation | ↑ ROS, ↑AIF release, ↑caspase activity, ↑Apoptosis | (Wang et al. 2012) |
|  |  |  |  | Nil | SW620 | ↑ ROS, ↑JNK, ↑ p38 MAPK, ↑release of cytochrome c, ↑caspase -3 & 8, ↑Apoptosis. | (Hsu et al. 2007) |
|  |  |  |  | Nil | SW480 | ↑ ROS , ↑AMPK, ↓integrin β1, ↑Apoptosis. | (Park et al. 2012) |
|  |  |  |  | Nil | LoVo | ↑ROS, ↓COX-2, ↓PGE2, ↓JAK2, ↓STAT3 | (Liu et al. 2015) |
|  |  |  |  | Nil | Azoxymethane or Dextran sulfate sodium-induced colon tumors in mice | ↓IL)-6, ↓IL-1β, ↓TNF-α, ↓cyclin D1, ↓Ki-67, ↓COX-2, ↓survivin, ↓NF-κB, ↑caspase-3 | (Deng et al. 2022) |
|  |  |  |  | Nil | SW620 and LoVo, BALB/c mice solid tumor model | ↑ROS, ↓COX-2/↓PGE2, ↓JAK2, ↓STAT3, ↓MMP-2/-9, ↑Apoptosis. | (Li, Hua, et al. 2015; Liu et al. 2015) |
|  |  |  |  | Berberine with *Andrographis paniculata* extract | HT-29, RKO | ↑ ROS, ↑alteration in genes involved DNA replication, ↑Apoptosis | (Zhao, Roy, et al. 2022) |
|  |  |  |  | Berberine with evodamine (indole alkaloid) | Caco-2, HT-29 | ↑ ROS, ↑Nrf2, ↑Apoptosis. | (Guan et al. 2020) |
|  | Cepharanthine (Biscoclurine or bis benzylisoquinoline alkaloid) | *Stephania cepharantha Hayanta* |  | Nil | HT-29, SW620 | ↑ROS, ↑cycle arrest, ↑ p21Waf1/Cip1 level, ↑Apoptosis. | (Rattanawong et al. 2018) |
|  | Coptisine (Quaternary proberberine alkaloid) | *Coptis chinensis Franch* |  | Nil | HCT-116 & HCT-116 BALB/c mice solid tumor model | ↑ROS, ↓Bcl-2, ↓Bcl-XL, ↓XIAP, ↑Bax,↑ Bad, ↑cytochrome c release, ↑AIF, ↑caspase-3, ↓PI3K, ↓Akt, ↑Apoptosis. | (Han et al. 2018) |
|  | Neferine (Isoquinoline alkaloid) | *Nelumbo nucifera* |  | Nil | HCT-15 | ↑ROS, ↑caspase-3 ↑MAPK, ↓PI3K, ↓Akt, ↓mTOR, ↑Apoptosis | (Manogaran et al. 2019; Manogaran, Somasundaram, and Viswanadha 2022) |
|  | Palmatine (Proto berberine alkaloid) | *Coptis chinensis* |  | Nil | HCT-116, SW480, HT-29 | ↑ROS levels, ↓Mitochondrial membrane potential, ↓AURKA, ↓Bcl-xl, ↓Bcl2, ↑caspase 3 & 9, ↑Apoptosis. | (Liu, Zhang, et al. 2020) |
|  | Tetrandrine (bisbenzylisoquinoline alkaloid) | *Stephania tetrandra* |  | Nil | HCT-116 | ↑ROS, ↓E2F1 ↑p53/p21Cip1, ↑Apoptosis | (Meng et al. 2004; Qin et al. 2013) |
|  | Xylopine (Benzylisoquinoline alkaloid) | *Xylopia laevigata* |  | Nil | HCT-116 | ↑ROS, ↑cycle arrest ↑, caspase-3, ↑Apoptosis . | (Santos et al. 2017) |
| **Indole alkaloids** | Camptothecin (Pyranoindolizinoquinoline) | *Camptotheca acuminata* |  | Nil | HCT-116 | ↑ROS, ↑DNA damage, ↑Bax, ↑p53, ↑p21, ↓COX-2, ↑TRAIL, ↓ NF-κB, ↑Apoptosis | (Ha et al. 2009) |
|  |  |  |  | Nil | HT-29 cells | ↑superoxide ROS , ↑caspase-3, ↓ Bcl-2, ↑Bax, ↑Bak, ↑p21, ↓COX-2, ↓NF-κB | (Wenzel et al. 2004) |
|  |  |  |  | Nil | CT-26 induced tumor BALB/c mouse | ↑ROS, ↑Mitochondria dysfunction, ↑DNA damage, ↑Cell cycle arrest, ↑Apoptosis | (Guo et al. 2022) |
|  |  |  |  | Camptothecin with triptolide | DLD1, HCT-116 | ↑Mitochondrial fission, ↓complex I activity, ↓mitochondrial size, ↑ROS, ↑Apoptosis. | (Wenzel et al. 2004) |
|  | Irinotecan/  Irinotecan hydrochloride (IH) (SN-38 active metabolite) | Semi synthetic derivative of Camptothecin |  | With 6-Hydroxymethylacylfulvene and 5-fluorouracil (5-FU) | HT-29 cells generated tumor | ↑ROS, ↓ growth | (Britten et al. 1999) |
|  |  |  |  | With thrombospondin-1 (TS-1) | HT-29 cells | No effect | (Allegrini et al. 2004; Raymond et al. 2002) |
|  |  |  |  |  | HT-29 cell tumor model | ↓Size of tumor | (Allegrini et al. 2004; Raymond et al. 2002) |
|  |  |  |  | With oxaliplatin, capecitabine, and bevacizumab | Advanced colon tumor | Extented survival time | (Cai et al. 2018) |
|  |  |  |  | 5-FU and oxaliplatin | Advanced colon tumor | Significant recovery | (Cai et al. 2018) |
|  |  |  |  | with 5-fluorouracil and oxaliplatin | Metastatic colorectal cancer (CRC) | Improves the patients' colon cancer condition and survival rate up to 30 months | (Fujita et al. 2015) |
| **Capsaicinoids** | Capsaicin (Phenylpropanoid alkaloids) | *Capsicum annuum* |  | Nil | Colo320DM, LoVo cells | ↑ROS, ↓ Mitochondrial membrane potential ↑caspase activity , ↑Apoptosis | (Yang et al. 2009) |
|  |  |  |  | Nil | HT-29 cells | ↑PPAR-γ, ↑AMPK, ↑Apoptosis | (Kim et al. 2004) |
|  |  |  |  | Nil | Colo 205 and Its tumor xenograft | ↑ROS, ↑Ca^+2^, ↑p53, ↑p21, ↑Mitochondrial dysfunction, ↑cytochrome c release, ↑Bax, ↓Bcl-2, ↑caspase-3, 8, &9, ↑Fas, ↑Apoptosis. | (Lu et al. 2010) |
|  |  |  |  | With 3,3'-Diindolylmethane | HCT116, SW480, LoVo, Caco-2, HT-29 | ↑ p53, ↑Fas, ↓NF-κB, ↑Apoptosis. | (Clark, Lee, and Lee 2015) |
|  |  |  |  | With resveratrol | HCT-116 cells | ↑ NO, ↑p53, ↓Mdm2, ↑Bax ↑Apoptosis | (Kim, Trudel, and Wogan 2009) |
| **Carbazole alkaloids** | Clausenidin (Pyrano coumarin) | *Clausena excavate* |  | Nil | HT-29 cells | ↑ROS, ↑Mitochondrial membrane depolarization, ↑cell cycle arrest, ↑caspase-9 activity, ↑Apoptosis. | (Waziri et al. 2016) |
| **Diterpenoid alkaloids** | Lappaconitine hydrochloride (C 18-diterpenoid alkaloid) | *Aconitum sinomontanum Nakai* |  | Nil | HCT-116 & HCT-116 cell xenograft | ↑ROS, ↓Mitochondrial membrane potential, ↑MAPK. | (Song et al. 2021) |
| **Piperidine alkaloids** | Piperine (Piperidine derivative) | *Piper nigrum* |  | Nil | HT-29 | ↑ROS, ↑ER stress, ↑Cell cycle arrest, ↓cyclin D1, D2, ↓ cyclin-dependent kinase inhibitor, ↑Apoptosis | (Yaffe et al. 2015) |
| **Amide alkaloids** | Piperlongumine (Pyridine derivative) | *Piper longum* |  | Nil | HCT-116 | ↑ ROS, ↓ glutathione S-transferase, ↑JNK, ↑MEK, ↓ERK, ↑Apoptosis. | (F et al. 2018) |
|  |  |  |  | With Oxaliplatin | HCT-116, LoVo cells, Metastatic colon cancer | ↑ROS, ↑mitochondrial dysfunction, ER stress and its related markers, ↑Apoptosis | (F et al. 2018; W. Chen et al. 2019) |
| Carboline alkaloids | Harmine (Pyrido Indole derivative) | *Peganum harmala* |  | Nil | HCT-116 | ↑ROS, ↑BAX, ↓Bcl-2, ↑PARP, ↓ERK, ↓PI3K, ↓Akt, ↓mTOR, ↑capase-3, ↑Apoptosis | (Kim 2021) |
|  |  |  |  | Nil | SW620 | ↑ROS, ↓Mitochondrial membrane potential, ↑cell cycle arrest, ↓ERK, ↓PI3K, ↓Akt, ↓mTOR, ↑capase-3, ↑Apoptosis | (Liu et al. 2016) |
| **Quinolizidine alkaloids** | Oxymatrine (Quinolizidine derivative) | *Sophora flavescens* |  | With doxorubicin | HT-29 & SW620 cells | ↑ROS, ↑cleaved caspase-3, ↑cleaved caspase-9, ↑ Bax/Bcl-2 ratio, ↑Apoptosis. | ( Pan et al. 2021) |
|  |  |  |  | With doxorubicin | HT-29 cells generated xenograft | ↑ROS, ↑E-cadherin, ↓N-cadherin | ( Pan et al. 2021) |
|  |  |  |  | With 5-fluorouracil (5-FU) | HCT-8 cells | ↑ROS, ↓SNAI2, E-cadherin, ↑ ↓NF-κB | (Liang et al. 2020) |
| **Proto alkaloids** | **Colchicine** | *Colchicum autumnale* |  | Nil | HT-29 | ↑ROS, ↓mitochondrial membrane integrity, ↑BAX, ↑caspase-3, ↑p38, JNK, ↓ AKT, ↑Apoptosis | (Huang, Xu, and Peng 2015) |
|  |  |  |  | Nil | HCT-116 and Colo-205 | ↑ROS, ↑microtubules depolarization, ↑cell cycle arrest, ↑Apoptosis | (Kumar et al. 2016) |

**References:**

Abotaleb, M., S. M. Samuel, E. Varghese, S. Varghese, P. Kubatka, A. Liskova, and D. Büsselberg. 2018. "Flavonoids in Cancer and Apoptosis." *Cancers (Basel)* 11 (1). <https://doi.org/10.3390/cancers11010028>.

Afroze, N., S. Pramodh, J. Shafarin, K. Bajbouj, M. Hamad, M. K. Sundaram, S. Haque, and A. Hussain. 2022. "Fisetin Deters Cell Proliferation, Induces Apoptosis, Alleviates Oxidative Stress and Inflammation in Human Cancer Cells, HeLa." *Int J Mol Sci* 23 (3). <https://doi.org/10.3390/ijms23031707>.

Al-Tamimi, M., A. Q. Khan, R. Anver, F. Ahmad, M. Mateo J, S. S. Raza, M. Alam, J. Buddenkotte, M. Steinhoff, and S. Uddin. 2022. "Pristimerin mediated anticancer effects and sensitization of human skin cancer cells through modulation of MAPK signaling pathways." *Biomed Pharmacother* 156: 113950. <https://doi.org/10.1016/j.biopha.2022.113950>.

Al Dhaheri, Y., S. Attoub, G. Ramadan, K. Arafat, K. Bajbouj, N. Karuvantevida, S. AbuQamar, A. Eid, and R. Iratni. 2014. "Carnosol induces ROS-mediated beclin1-independent autophagy and apoptosis in triple negative breast cancer." *PLoS One* 9 (10): e109630. <https://doi.org/10.1371/journal.pone.0109630>.

Alam, M. M., R. Kariya, P. Boonnate, A. Kawaguchi, and S. Okada. 2021. "Induction of apoptosis by Shikonin through ROS-mediated intrinsic and extrinsic apoptotic pathways in primary effusion lymphoma." *Transl Oncol* 14 (3): 101006. <https://doi.org/10.1016/j.tranon.2020.101006>.

Alharbi, Y., A. Kapur, M. Felder, L. Barroilhet, T. Stein, B. R. Pattnaik, and M. S. Patankar. 2019. "Plumbagin-induced oxidative stress leads to inhibition of Na(+)/K(+)-ATPase (NKA) in canine cancer cells." *Sci Rep* 9 (1): 11471. <https://doi.org/10.1038/s41598-019-47261-x>.

Allegrini, G., F. A. Goulette, J. W. Darnowski, and P. Calabresi. 2004. "Thrombospondin-1 plus irinotecan: a novel antiangiogenic-chemotherapeutic combination that inhibits the growth of advanced human colon tumor xenografts in mice." *Cancer Chemother Pharmacol* 53 (3): 261-6. <https://doi.org/10.1007/s00280-003-0712-y>.

An, K., Y. Zhang, Y. Liu, S. Yan, Z. Hou, M. Cao, G. Liu, C. Dong, J. Gao, and G. Liu. 2020. "Neferine induces apoptosis by modulating the ROS‑mediated JNK pathway in esophageal squamous cell carcinoma." *Oncol Rep* 44 (3): 1116-1126. <https://doi.org/10.3892/or.2020.7675>.

Banerjee, A., V. Banerjee, S. Czinn, and T. Blanchard. 2017. "Increased reactive oxygen species levels cause ER stress and cytotoxicity in andrographolide treated colon cancer cells." *Oncotarget* 8 (16): 26142-26153. <https://doi.org/10.18632/oncotarget.15393>.

Banerjee, M., S. Chattopadhyay, T. Choudhuri, R. Bera, S. Kumar, B. Chakraborty, and S. K. Mukherjee. 2016. "Cytotoxicity and cell cycle arrest induced by andrographolide lead to programmed cell death of MDA-MB-231 breast cancer cell line." *J Biomed Sci* 23: 40. <https://doi.org/10.1186/s12929-016-0257-0>.

Benzina, S., J. Harquail, S. Jean, A. P. Beauregard, C. D. Colquhoun, M. Carroll, A. Bos, C. A. Gray, and G. A. Robichaud. 2015. "Deoxypodophyllotoxin isolated from Juniperus communis induces apoptosis in breast cancer cells." *Anticancer Agents Med Chem* 15 (1): 79-88. <https://doi.org/10.2174/1871520614666140608150448>.

Bhardwaj, M., H. J. Cho, S. Paul, R. Jakhar, I. Khan, S. J. Lee, B. Y. Kim, M. Krishnan, T. P. Khaket, H. G. Lee, and S. C. Kang. 2018. "Vitexin induces apoptosis by suppressing autophagy in multi-drug resistant colorectal cancer cells." *Oncotarget* 9 (3): 3278-3291. <https://doi.org/10.18632/oncotarget.22890>.

Bo, S., J. Lai, H. Lin, X. Luo, Y. Zeng, and T. Du. 2021. "Purpurin, a anthraquinone induces ROS-mediated A549 lung cancer cell apoptosis via inhibition of PI3K/AKT and proliferation." *J Pharm Pharmacol* 73 (8): 1101-1108. <https://doi.org/10.1093/jpp/rgab056>.

Britten, C. D., S. G. Hilsenbeck, S. G. Eckhardt, J. Marty, G. Mangold, J. R. MacDonald, E. K. Rowinsky, D. D. Von Hoff, and S. Weitman. 1999. "Enhanced antitumor activity of 6-hydroxymethylacylfulvene in combination with irinotecan and 5-fluorouracil in the HT29 human colon tumor xenograft model." *Cancer Res* 59 (5): 1049-53.

Cai, J., X. Niu, Y. Chen, Q. Hu, G. Shi, H. Wu, J. Wang, and J. Yi. 2008. "Emodin-induced generation of reactive oxygen species inhibits RhoA activation to sensitize gastric carcinoma cells to anoikis." *Neoplasia* 10 (1): 41-51. <https://doi.org/10.1593/neo.07754>.

Cai, Y., R. Deng, H. Hu, J. Zhang, J. Ling, Z. Wu, L. Yang, J. Li, and Y. Deng. 2018. "[Analysis on safety and preliminary efficacy of dose-modified regimen of 5-fluorouracil plus oxaliplatin and irinotecan (FOLFOXIRI) in advanced colorectal cancer]." *Zhonghua Wei Chang Wai Ke Za Zhi* 21 (9): 1045-1050.

Cao, J., H. Wang, F. Chen, J. Fang, A. Xu, W. Xi, S. Zhang, G. Wu, and Z. Wang. 2016. "Galangin inhibits cell invasion by suppressing the epithelial-mesenchymal transition and inducing apoptosis in renal cell carcinoma." *Mol Med Rep* 13 (5): 4238-44. <https://doi.org/10.3892/mmr.2016.5042>.

Chaitanya, Mvnl, A. K. Ramanunny, M. R. Babu, M. Gulati, S. Vishwas, T. G. Singh, D. K. Chellappan, J. Adams, K. Dua, and S. K. Singh. 2022. "Journey of Rosmarinic Acid as Biomedicine to Nano-Biomedicine for Treating Cancer: Current Strategies and Future Perspectives." *Pharmaceutics* 14 (11). <https://doi.org/10.3390/pharmaceutics14112401>.

Chang, Y. M., Y. L. Shih, C. P. Chen, K. L. Liu, M. H. Lee, M. Z. Lee, H. T. Hou, H. C. Huang, H. F. Lu, S. F. Peng, K. W. Chen, M. Y. Yeh, and J. G. Chung. 2019. "Ouabain induces apoptotic cell death in human prostate DU 145 cancer cells through DNA damage and TRAIL pathways." *Environ Toxicol* 34 (12): 1329-1339. <https://doi.org/10.1002/tox.22834>.

Chen, C. Y., K. C. Chen, T. Y. Yang, H. C. Liu, and S. L. Hsu. 2013. "Gallic Acid Induces a Reactive Oxygen Species-Provoked c-Jun NH2-Terminal Kinase-Dependent Apoptosis in Lung Fibroblasts." *Evid Based Complement Alternat Med* 2013: 613950. <https://doi.org/10.1155/2013/613950>.

Chen, J. C., K. W. Lu, J. H. Lee, C. C. Yeh, and J. G. Chung. 2006. "Gypenosides induced apoptosis in human colon cancer cells through the mitochondria-dependent pathways and activation of caspase-3." *Anticancer Res* 26 (6b): 4313-26.

Chen, J. C., K. W. Lu, M. L. Tsai, S. C. Hsu, C. L. Kuo, J. S. Yang, T. C. Hsia, C. S. Yu, S. T. Chou, M. C. Kao, J. G. Chung, and W. G. Wood. 2009. "Gypenosides induced G0/G1 arrest via CHk2 and apoptosis through endoplasmic reticulum stress and mitochondria-dependent pathways in human tongue cancer SCC-4 cells." *Oral Oncol* 45 (3): 273-83. <https://doi.org/10.1016/j.oraloncology.2008.05.012>.

Chen, J., B. Chen, Z. Zou, W. Li, Y. Zhang, J. Xie, and C. Liu. 2017. "Costunolide enhances doxorubicin-induced apoptosis in prostate cancer cells via activated mitogen-activated protein kinases and generation of reactive oxygen species." *Oncotarget* 8 (64): 107701-107715. <https://doi.org/10.18632/oncotarget.22592>.

Chen, J., Y. Zhu, W. Zhang, X. Peng, J. Zhou, F. Li, B. Han, X. Liu, Y. Ou, and X. Yu. 2018. "Delphinidin induced protective autophagy via mTOR pathway suppression and AMPK pathway activation in HER-2 positive breast cancer cells." *BMC Cancer* 18 (1): 342. <https://doi.org/10.1186/s12885-018-4231-y>.

Chen, W., P. Li, Y. Liu, Y. Yang, X. Ye, F. Zhang, and H. Huang. 2018. "Isoalantolactone induces apoptosis through ROS-mediated ER stress and inhibition of STAT3 in prostate cancer cells." *J Exp Clin Cancer Res* 37 (1): 309. <https://doi.org/10.1186/s13046-018-0987-9>.

Chen, W., W. Lian, Y. Yuan, and M. Li. 2019. "The synergistic effects of oxaliplatin and piperlongumine on colorectal cancer are mediated by oxidative stress." *Cell Death Dis* 10 (8): 600. <https://doi.org/10.1038/s41419-019-1824-6>.

Chen, X., L. Pei, Z. Zhong, J. Guo, Q. Zhang, and Y. Wang. 2011. "Anti-tumor potential of ethanol extract of Curcuma phaeocaulis Valeton against breast cancer cells." *Phytomedicine* 18 (14): 1238-43. <https://doi.org/10.1016/j.phymed.2011.06.017>.

Chen, X., Y. Zhao, W. Luo, S. Chen, F. Lin, X. Zhang, S. Fan, X. Shen, Y. Wang, and G. Liang. 2020. "Celastrol induces ROS-mediated apoptosis via directly targeting peroxiredoxin-2 in gastric cancer cells." *Theranostics* 10 (22): 10290-10308. <https://doi.org/10.7150/thno.46728>.

Chen, Y. J., C. S. Wu, J. J. Shieh, J. H. Wu, H. Y. Chen, T. W. Chung, Y. K. Chen, and C. C. Lin. 2013. "Baicalein Triggers Mitochondria-Mediated Apoptosis and Enhances the Antileukemic Effect of Vincristine in Childhood Acute Lymphoblastic Leukemia CCRF-CEM Cells." *Evid Based Complement Alternat Med* 2013: 124747. <https://doi.org/10.1155/2013/124747>.

Chen, Y., S. H. Tseng, H. S. Lai, and W. J. Chen. 2004. "Resveratrol-induced cellular apoptosis and cell cycle arrest in neuroblastoma cells and antitumor effects on neuroblastoma in mice." *Surgery* 136 (1): 57-66. <https://doi.org/10.1016/j.surg.2004.01.017>.

Cheng, Q., Y. He, G. Li, Y. Liu, W. Gao, and L. Huang. 2013. "Effects of combined elicitors on tanshinone metabolic profiling and SmCPS expression in Salvia miltiorrhiza hairy root cultures." *Molecules* 18 (7): 7473-85. <https://doi.org/10.3390/molecules18077473>.

Chien, C. C., M. S. Wu, S. W. Chou, G. Jargalsaikhan, and Y. C. Chen. 2021. "Roles of reactive oxygen species, mitochondrial membrane potential, and p53 in evodiamine-induced apoptosis and G2/M arrest of human anaplastic thyroid carcinoma cells." *Chin Med* 16 (1): 134. <https://doi.org/10.1186/s13020-021-00505-3>.

Cho, H. D., J. H. Lee, K. D. Moon, K. H. Park, M. K. Lee, and K. I. Seo. 2018. "Auriculasin-induced ROS causes prostate cancer cell death via induction of apoptosis." *Food Chem Toxicol* 111: 660-669. <https://doi.org/10.1016/j.fct.2017.12.007>.

Choi, E. O., C. Park, H. J. Hwang, S. H. Hong, G. Y. Kim, E. J. Cho, W. J. Kim, and Y. H. Choi. 2016. "Baicalein induces apoptosis via ROS-dependent activation of caspases in human bladder cancer 5637 cells." *Int J Oncol* 49 (3): 1009-18. <https://doi.org/10.3892/ijo.2016.3606>.

Choi, H. G., P. T. Tran, J. H. Lee, B. S. Min, and J. A. Kim. 2018. "Anti-inflammatory activity of caffeic acid derivatives isolated from the roots of Salvia miltiorrhiza Bunge." *Arch Pharm Res* 41 (1): 64-70. <https://doi.org/10.1007/s12272-017-0983-1>.

Choi, J. B., J. H. Kim, H. Lee, J. N. Pak, B. S. Shim, and S. H. Kim. 2018. "Reactive Oxygen Species and p53 Mediated Activation of p38 and Caspases is Critically Involved in Kaempferol Induced Apoptosis in Colorectal Cancer Cells." *J Agric Food Chem* 66 (38): 9960-9967. <https://doi.org/10.1021/acs.jafc.8b02656>.

Choi, Y. H. 2019. "Isorhamnetin induces ROS-dependent cycle arrest at G2/M phase and apoptosis in human hepatocarcinoma Hep3B cells." *Gen Physiol Biophys* 38 (6): 473-484. <https://doi.org/10.4149/gpb_2019038>.

Chou, G. L., S. F. Peng, C. L. Liao, H. C. Ho, K. W. Lu, J. C. Lien, M. J. Fan, K. C. La, and J. G. Chung. 2018. "Casticin impairs cell growth and induces cell apoptosis via cell cycle arrest in human oral cancer SCC-4 cells." *Environ Toxicol* 33 (2): 127-141. <https://doi.org/10.1002/tox.22497>.

Chou, W. H., K. L. Liu, Y. L. Shih, Y. Y. Chuang, J. Chou, H. F. Lu, H. W. Jair, M. Z. Lee, M. K. Au, and J. G. Chung. 2018. "Ouabain Induces Apoptotic Cell Death Through Caspase- and Mitochondria-dependent Pathways in Human Osteosarcoma U-2 OS Cells." *Anticancer Res* 38 (1): 169-178. <https://doi.org/10.21873/anticanres.12205>.

Chu, P., H. Li, R. Luo, A. Ahsan, E. Qaed, A. Shopit, X. Ma, Y. Lin, J. Peng, J. Zhang, S. Wang, and Z. Tang. 2017. "Oleanolic acid derivative SZC014 inhibit cell proliferation and induce apoptosis of human breast cancer cells in a ROS-dependent way." *Neoplasma* 64 (5): 681-692. <https://doi.org/10.4149/neo_2017_505>.

Chu, Y. L., C. T. Ho, J. G. Chung, R. Raghu, Y. C. Lo, and L. Y. Sheen. 2013. "Allicin induces anti-human liver cancer cells through the p53 gene modulating apoptosis and autophagy." *J Agric Food Chem* 61 (41): 9839-48. <https://doi.org/10.1021/jf403241s>.

Chung, Y. H., and D. Kim. 2016. "RIP kinase-mediated ROS production triggers XAF1 expression through activation of TAp73 in casticin-treated bladder cancer cells." *Oncol Rep* 36 (2): 1135-42. <https://doi.org/10.3892/or.2016.4895>.

Clark, R., J. Lee, and S. H. Lee. 2015. "Synergistic anticancer activity of capsaicin and 3,3'-diindolylmethane in human colorectal cancer." *J Agric Food Chem* 63 (17): 4297-304. <https://doi.org/10.1021/jf506098s>.

Dai, W., L. Mu, Y. Cui, Y. Li, P. Chen, H. Xie, and X. Wang. 2019. "Berberine Promotes Apoptosis of Colorectal Cancer via Regulation of the Long Non-Coding RNA (lncRNA) Cancer Susceptibility Candidate 2 (CASC2)/AU-Binding Factor 1 (AUF1)/B-Cell CLL/Lymphoma 2 (Bcl-2) Axis." *Med Sci Monit* 25: 730-738. <https://doi.org/10.12659/msm.912082>.

Deng, J., L. Zhao, X. Yuan, Y. Li, J. Shi, H. Zhang, Y. Zhao, L. Han, H. Wang, Y. Yan, H. Zhao, H. Wang, and F. Zou. 2022. "Pre-Administration of Berberine Exerts Chemopreventive Effects in AOM/DSS-Induced Colitis-Associated Carcinogenesis Mice via Modulating Inflammation and Intestinal Microbiota." *Nutrients* 14 (4). <https://doi.org/10.3390/nu14040726>.

Dey, D. K., S. N. Chang, Y. Vadlamudi, J. G. Park, and S. C. Kang. 2020. "Synergistic therapy with tangeretin and 5-fluorouracil accelerates the ROS/JNK mediated apoptotic pathway in human colorectal cancer cell." *Food Chem Toxicol* 143: 111529. <https://doi.org/10.1016/j.fct.2020.111529>.

Doi, H., T. Matsui, J. M. Dijkstra, A. Ogasawara, Y. Higashimoto, S. Imamura, T. Ohye, H. Takematsu, I. Katsuda, and H. Akiyama. 2021. "Andrographolide, isolated from Andrographis paniculata, induces apoptosis in monocytic leukemia and multiple myeloma cells via augmentation of reactive oxygen species production." *F1000Res* 10: 542. <https://doi.org/10.12688/f1000research.53595.3>.

Dong, Y., A. Cao, J. Shi, P. Yin, L. Wang, G. Ji, J. Xie, and D. Wu. 2014. "Tangeretin, a citrus polymethoxyflavonoid, induces apoptosis of human gastric cancer AGS cells through extrinsic and intrinsic signaling pathways." *Oncol Rep* 31 (4): 1788-94. <https://doi.org/10.3892/or.2014.3034>.

Duan, W. J., Q. S. Li, M. Y. Xia, S. Tashiro, S. Onodera, and T. Ikejima. 2011. "Silibinin activated p53 and induced autophagic death in human fibrosarcoma HT1080 cells via reactive oxygen species-p38 and c-Jun N-terminal kinase pathways." *Biol Pharm Bull* 34 (1): 47-53. <https://doi.org/10.1248/bpb.34.47>.

Efferth, T., M. Giaisi, A. Merling, P. H. Krammer, and M. Li-Weber. 2007. "Artesunate induces ROS-mediated apoptosis in doxorubicin-resistant T leukemia cells." *PLoS One* 2 (8): e693. <https://doi.org/10.1371/journal.pone.0000693>.

Estévez, S., M. T. Marrero, J. Quintana, and F. Estévez. 2014. "Eupatorin-induced cell death in human leukemia cells is dependent on caspases and activates the mitogen-activated protein kinase pathway." *PLoS One* 9 (11): e112536. <https://doi.org/10.1371/journal.pone.0112536>.

F, D. A. Silva Machado, F. M. Munari, F. J. Scariot, S. Echeverrigaray, C. Aguzzoli, C. T. Pich, M. J. Kato, L. Yamaguchi, S. Moura, J. A. P. Henriques, and M. Roesch-Ely. 2018. "Piperlongumine Induces Apoptosis in Colorectal Cancer HCT 116 Cells Independent of Bax, p21 and p53 Status." *Anticancer Res* 38 (11): 6231-6236. <https://doi.org/10.21873/anticanres.12978>.

Fan, C., Y. Yang, Y. Liu, S. Jiang, S. Di, W. Hu, Z. Ma, T. Li, Y. Zhu, Z. Xin, G. Wu, J. Han, X. Li, and X. Yan. 2016. "Icariin displays anticancer activity against human esophageal cancer cells via regulating endoplasmic reticulum stress-mediated apoptotic signaling." *Sci Rep* 6: 21145. <https://doi.org/10.1038/srep21145>.

Fang, C., J. Zhang, D. Qi, X. Fan, J. Luo, L. Liu, and Q. Tan. 2014. "Evodiamine induces G2/M arrest and apoptosis via mitochondrial and endoplasmic reticulum pathways in H446 and H1688 human small-cell lung cancer cells." *PLoS One* 9 (12): e115204. <https://doi.org/10.1371/journal.pone.0115204>.

Flores-Lopez, G., D. Moreno-Lorenzana, M. Ayala-Sanchez, S. Aviles-Vazquez, H. Torres-Martinez, P. A. Crooks, M. L. Guzman, H. Mayani, and A. Chávez-González. 2018. "Parthenolide and DMAPT induce cell death in primitive CML cells through reactive oxygen species." *J Cell Mol Med* 22 (10): 4899-4912. <https://doi.org/10.1111/jcmm.13755>.

Fofaria, N. M., S. H. Kim, and S. K. Srivastava. 2014. "Piperine causes G1 phase cell cycle arrest and apoptosis in melanoma cells through checkpoint kinase-1 activation." *PLoS One* 9 (5): e94298. <https://doi.org/10.1371/journal.pone.0094298>.

Fu, D., D. Wu, W. Cheng, J. Gao, Z. Zhang, J. Ge, W. Zhou, and Z. Xu. 2020. "Costunolide Induces Autophagy and Apoptosis by Activating ROS/MAPK Signaling Pathways in Renal Cell Carcinoma." *Front Oncol* 10: 582273. <https://doi.org/10.3389/fonc.2020.582273>.

Fujita, K., Y. Kubota, H. Ishida, and Y. Sasaki. 2015. "Irinotecan, a key chemotherapeutic drug for metastatic colorectal cancer." *World J Gastroenterol* 21 (43): 12234-48. <https://doi.org/10.3748/wjg.v21.i43.12234>.

Gao, H., H. Wang, and J. Peng. 2014. "Hispidulin induces apoptosis through mitochondrial dysfunction and inhibition of P13k/Akt signalling pathway in HepG2 cancer cells." *Cell Biochem Biophys* 69 (1): 27-34. <https://doi.org/10.1007/s12013-013-9762-x>.

Gong, L. H., X. X. Chen, H. Wang, Q. W. Jiang, S. S. Pan, J. G. Qiu, X. L. Mei, Y. Q. Xue, W. M. Qin, F. Y. Zheng, Z. Shi, and X. J. Yan. 2014. "Piperlongumine induces apoptosis and synergizes with cisplatin or paclitaxel in human ovarian cancer cells." *Oxid Med Cell Longev* 2014: 906804. <https://doi.org/10.1155/2014/906804>.

Gong, X., Z. Chen, Q. Han, C. Chen, L. Jing, Y. Liu, L. Zhao, X. Yao, and X. Sun. 2018. "Sanguinarine triggers intrinsic apoptosis to suppress colorectal cancer growth through disassociation between STRAP and MELK." *BMC Cancer* 18 (1): 578. <https://doi.org/10.1186/s12885-018-4463-x>.

Gu, J. J., K. S. Qiao, P. Sun, P. Chen, and Q. Li. 2018. "Study of EGCG induced apoptosis in lung cancer cells by inhibiting PI3K/Akt signaling pathway." *Eur Rev Med Pharmacol Sci* 22 (14): 4557-4563. <https://doi.org/10.26355/eurrev_201807_15511>.

Guo, Y. X., Z. M. Lin, M. J. Wang, Y. W. Dong, H. M. Niu, C. Y. Young, H. X. Lou, and H. Q. Yuan. 2016. "Jungermannenone A and B induce ROS- and cell cycle-dependent apoptosis in prostate cancer cells in vitro." *Acta Pharmacol Sin* 37 (6): 814-24. <https://doi.org/10.1038/aps.2016.26>.

Guo, Y., W. Zhang, Y. Y. Yan, C. G. Ma, X. Wang, C. Wang, and J. L. Zhao. 2013. "Triterpenoid pristimerin induced HepG2 cells apoptosis through ROS-mediated mitochondrial dysfunction." *J buon* 18 (2): 477-85.

Guo, Z., Z. Wang, R. Liang, H. Tian, X. Chen, and M. Chen. 2022. "Reactive oxygen species activated by mitochondria-specific camptothecin prodrug for enhanced chemotherapy." *Bosn J Basic Med Sci* 22 (6): 934-948. <https://doi.org/10.17305/bjbms.2022.7194>.

Gupta, P., S. E. Wright, S. H. Kim, and S. K. Srivastava. 2014. "Phenethyl isothiocyanate: a comprehensive review of anti-cancer mechanisms." *Biochim Biophys Acta* 1846 (2): 405-24. <https://doi.org/10.1016/j.bbcan.2014.08.003>.

Gurunathan, S., M. Jeyaraj, M. H. Kang, and J. H. Kim. 2019. "Tangeretin-Assisted Platinum Nanoparticles Enhance the Apoptotic Properties of Doxorubicin: Combination Therapy for Osteosarcoma Treatment." *Nanomaterials (Basel)* 9 (8). <https://doi.org/10.3390/nano9081089>.

Ha, S. W., Y. J. Kim, W. Kim, and C. S. Lee. 2009. "Antitumor Effects of Camptothecin Combined with Conventional Anticancer Drugs on the Cervical and Uterine Squamous Cell Carcinoma Cell Line SiHa." *Korean J Physiol Pharmacol* 13 (2): 115-21. <https://doi.org/10.4196/kjpp.2009.13.2.115>.

Hamacher-Brady, A., H. A. Stein, S. Turschner, I. Toegel, R. Mora, N. Jennewein, T. Efferth, R. Eils, and N. R. Brady. 2011. "Artesunate activates mitochondrial apoptosis in breast cancer cells via iron-catalyzed lysosomal reactive oxygen species production." *J Biol Chem* 286 (8): 6587-601. <https://doi.org/10.1074/jbc.M110.210047>.

Han, B. J., W. Li, G. B. Jiang, S. H. Lai, C. Zhang, C. C. Zeng, and Y. J. Liu. 2015. "Effects of daidzein in regards to cytotoxicity in vitro, apoptosis, reactive oxygen species level, cell cycle arrest and the expression of caspase and Bcl-2 family proteins." *Oncol Rep* 34 (3): 1115-20. <https://doi.org/10.3892/or.2015.4133>.

Han, B., P. Jiang, Z. Li, Y. Yu, T. Huang, X. Ye, and X. Li. 2018. "Coptisine-induced apoptosis in human colon cancer cells (HCT-116) is mediated by PI3K/Akt and mitochondrial-associated apoptotic pathway." *Phytomedicine* 48: 152-160. <https://doi.org/10.1016/j.phymed.2017.12.027>.

Han, L. L., L. P. Xie, L. H. Li, X. W. Zhang, R. Q. Zhang, and H. Z. Wang. 2009. "Reactive oxygen species production and Bax/Bcl-2 regulation in honokiol-induced apoptosis in human hepatocellular carcinoma SMMC-7721 cells." *Environ Toxicol Pharmacol* 28 (1): 97-103. <https://doi.org/10.1016/j.etap.2009.03.005>.

Han, Q., H. Wang, C. Xiao, B. D. Fu, and C. T. Du. 2017. "Oroxylin A inhibits H(2)O(2)-induced oxidative stress in PC12 cells." *Nat Prod Res* 31 (11): 1339-1342. <https://doi.org/10.1080/14786419.2016.1244193>.

Hassannia, B., B. Wiernicki, I. Ingold, F. Qu, S. Van Herck, Y. Y. Tyurina, H. Bayır, B. A. Abhari, J. P. F. Angeli, S. M. Choi, E. Meul, K. Heyninck, K. Declerck, C. S. Chirumamilla, M. Lahtela-Kakkonen, G. Van Camp, D. V. Krysko, P. G. Ekert, S. Fulda, B. G. De Geest, M. Conrad, V. E. Kagan, W. Vanden Berghe, P. Vandenabeele, and T. Vanden Berghe. 2018. "Nano-targeted induction of dual ferroptotic mechanisms eradicates high-risk neuroblastoma." *J Clin Invest* 128 (8): 3341-3355. <https://doi.org/10.1172/jci99032>.

He, F., Q. Wang, X. L. Zheng, J. Q. Yan, L. Yang, H. Sun, L. N. Hu, Y. Lin, and X. Wang. 2012. "Wogonin potentiates cisplatin-induced cancer cell apoptosis through accumulation of intracellular reactive oxygen species." *Oncol Rep* 28 (2): 601-5. <https://doi.org/10.3892/or.2012.1841>.

Heng, W. S., and S. C. Cheah. 2020. "Chelerythrine Chloride Downregulates β-Catenin and Inhibits Stem Cell Properties of Non-Small Cell Lung Carcinoma." *Molecules* 25 (1). <https://doi.org/10.3390/molecules25010224>.

Heo, J. R., S. M. Kim, K. A. Hwang, J. H. Kang, and K. C. Choi. 2018. "Resveratrol induced reactive oxygen species and endoplasmic reticulum stress‑mediated apoptosis, and cell cycle arrest in the A375SM malignant melanoma cell line." *Int J Mol Med* 42 (3): 1427-1435. <https://doi.org/10.3892/ijmm.2018.3732>.

Heo, S. J., and Y. J. Jeon. 2009. "Protective effect of fucoxanthin isolated from Sargassum siliquastrum on UV-B induced cell damage." *J Photochem Photobiol B* 95 (2): 101-7. <https://doi.org/10.1016/j.jphotobiol.2008.11.011>.

Hoffmann, R., K. von Schwarzenberg, N. López-Antón, A. Rudy, G. Wanner, V. M. Dirsch, and A. M. Vollmar. 2011. "Helenalin bypasses Bcl-2-mediated cell death resistance by inhibiting NF-κB and promoting reactive oxygen species generation." *Biochem Pharmacol* 82 (5): 453-63. <https://doi.org/10.1016/j.bcp.2011.05.029>.

Hou, D. X., X. Tong, N. Terahara, D. Luo, and M. Fujii. 2005. "Delphinidin 3-sambubioside, a Hibiscus anthocyanin, induces apoptosis in human leukemia cells through reactive oxygen species-mediated mitochondrial pathway." *Arch Biochem Biophys* 440 (1): 101-9. <https://doi.org/10.1016/j.abb.2005.06.002>.

Hsiao, C. H., C. J. Yao, G. M. Lai, L. M. Lee, J. Whang-Peng, and P. H. Shih. 2019. "Honokiol induces apoptotic cell death by oxidative burst and mitochondrial hyperpolarization of bladder cancer cells." *Exp Ther Med* 17 (5): 4213-4222. <https://doi.org/10.3892/etm.2019.7419>.

Hsiao, Y. C., S. F. Peng, K. C. Lai, C. L. Liao, Y. P. Huang, C. C. Lin, M. L. Lin, K. C. Liu, C. C. Tsai, Y. S. Ma, and J. G. Chung. 2019. "Genistein induces apoptosis in vitro and has antitumor activity against human leukemia HL-60 cancer cell xenograft growth in vivo." *Environ Toxicol* 34 (4): 443-456. <https://doi.org/10.1002/tox.22698>.

Hsu, H. Y., J. S. Yang, K. W. Lu, C. S. Yu, S. T. Chou, J. J. Lin, Y. Y. Chen, M. L. Lin, F. S. Chueh, S. S. Chen, and J. G. Chung. 2011. "An experimental study on the antileukemia effects of gypenosides in vitro and in vivo." *Integr Cancer Ther* 10 (1): 101-12. <https://doi.org/10.1177/1534735410377198>.

Hsu, W. H., Y. S. Hsieh, H. C. Kuo, C. Y. Teng, H. I. Huang, C. J. Wang, S. F. Yang, Y. S. Liou, and W. H. Kuo. 2007. "Berberine induces apoptosis in SW620 human colonic carcinoma cells through generation of reactive oxygen species and activation of JNK/p38 MAPK and FasL." *Arch Toxicol* 81 (10): 719-28. <https://doi.org/10.1007/s00204-006-0169-y>.

Huang, H., H. Xie, Y. Pan, K. Zheng, Y. Xia, and W. Chen. 2018. "Plumbagin Triggers ER Stress-Mediated Apoptosis in Prostate Cancer Cells via Induction of ROS." *Cell Physiol Biochem* 45 (1): 267-280. <https://doi.org/10.1159/000486773>.

Huang, H., J. K. Yi, S. G. Lim, S. Park, H. Zhang, E. Kim, S. Jang, M. H. Lee, K. Liu, K. R. Kim, E. K. Kim, Y. Lee, S. H. Kim, Z. Y. Ryoo, and M. O. Kim. 2021. "Costunolide Induces Apoptosis via the Reactive Oxygen Species and Protein Kinase B Pathway in Oral Cancer Cells." *Int J Mol Sci* 22 (14). <https://doi.org/10.3390/ijms22147509>.

Huang, K., Y. Chen, R. Zhang, Y. Wu, Y. Ma, X. Fang, and S. Shen. 2018. "Honokiol induces apoptosis and autophagy via the ROS/ERK1/2 signaling pathway in human osteosarcoma cells in vitro and in vivo." *Cell Death Dis* 9 (2): 157. <https://doi.org/10.1038/s41419-017-0166-5>.

Huang, Z., S. Gan, X. Zhuang, Y. Chen, L. Lu, Y. Wang, X. Qi, Q. Feng, Q. Huang, B. Du, R. Zhang, and Z. Liu. 2022. "Artesunate Inhibits the Cell Growth in Colorectal Cancer by Promoting ROS-Dependent Cell Senescence and Autophagy." *Cells* 11 (16). <https://doi.org/10.3390/cells11162472>.

Huang, Z., Y. Xu, and W. Peng. 2015. "Colchicine induces apoptosis in HT‑29 human colon cancer cells via the AKT and c-Jun N-terminal kinase signaling pathways." *Mol Med Rep* 12 (4): 5939-44. <https://doi.org/10.3892/mmr.2015.4222>.

Ilango, S., D. K. Sahoo, B. Paital, K. Kathirvel, J. I. Gabriel, K. Subramaniam, P. Jayachandran, R. K. Dash, A. K. Hati, T. R. Behera, P. Mishra, and R. Nirmaladevi. 2022. "A Review on Annona muricata and Its Anticancer Activity." *Cancers (Basel)* 14 (18). <https://doi.org/10.3390/cancers14184539>.

Iyappan, P., M. D. Bala, M. Sureshkumar, V. P. Veeraraghavan, and A. Palanisamy. 2021. "Fucoxanthin induced apoptotic cell death in oral squamous carcinoma (KB) cells." *Bioinformation* 17 (1): 181-191. <https://doi.org/10.6026/97320630017181>.

Jaganathan, S. K., A. Mazumdar, D. Mondhe, and M. Mandal. 2011. "Apoptotic effect of eugenol in human colon cancer cell lines." *Cell Biol Int* 35 (6): 607-15. <https://doi.org/10.1042/cbi20100118>.

Jaiswal, A., A. Sabarwal, J. P. Narayan Mishra, and R. P. Singh. 2018. "Plumbagin induces ROS-mediated apoptosis and cell cycle arrest and inhibits EMT in human cervical carcinoma cells." *RSC Adv* 8 (56): 32022-32037. <https://doi.org/10.1039/c8ra05339a>.

Jang, J. H., T. Iqbal, K. J. Min, S. Kim, J. W. Park, E. I. Son, T. J. Lee, and T. K. Kwon. 2013. "Helenalin-induced apoptosis is dependent on production of reactive oxygen species and independent of induction of endoplasmic reticulum stress in renal cell carcinoma." *Toxicol In Vitro* 27 (2): 588-96. <https://doi.org/10.1016/j.tiv.2012.10.014>.

Jeong, C. H., H. Ryu, D. H. Kim, W. N. Cheng, J. E. Yoon, S. Kang, and S. G. Han. 2019. "Piperlongumine Induces Cell Cycle Arrest via Reactive Oxygen Species Accumulation and IKKβ Suppression in Human Breast Cancer Cells." *Antioxidants (Basel)* 8 (11). <https://doi.org/10.3390/antiox8110553>.

Ji, B. C., W. H. Hsu, J. S. Yang, T. C. Hsia, C. C. Lu, J. H. Chiang, J. L. Yang, C. H. Lin, J. J. Lin, L. J. Suen, W. Gibson Wood, and J. G. Chung. 2009. "Gallic acid induces apoptosis via caspase-3 and mitochondrion-dependent pathways in vitro and suppresses lung xenograft tumor growth in vivo." *J Agric Food Chem* 57 (16): 7596-604. <https://doi.org/10.1021/jf901308p>.

Jiang, C., M. Masood, A. Rasul, W. Wei, Y. Wang, M. Ali, M. Mustaqeem, J. Li, and X. Li. 2017. "Altholactone Inhibits NF-κB and STAT3 Activation and Induces Reactive Oxygen Species-Mediated Apoptosis in Prostate Cancer DU145 Cells." *Molecules* 22 (2). <https://doi.org/10.3390/molecules22020240>.

Jiang, K., W. Wang, X. Jin, Z. Wang, Z. Ji, and G. Meng. 2015. "Silibinin, a natural flavonoid, induces autophagy via ROS-dependent mitochondrial dysfunction and loss of ATP involving BNIP3 in human MCF7 breast cancer cells." *Oncol Rep* 33 (6): 2711-8. <https://doi.org/10.3892/or.2015.3915>.

Jiang, Y., X. Wang, and D. Hu. 2017. "Furanodienone induces G0/G1 arrest and causes apoptosis via the ROS/MAPKs-mediated caspase-dependent pathway in human colorectal cancer cells: a study in vitro and in vivo." *Cell Death Dis* 8 (5): e2815. <https://doi.org/10.1038/cddis.2017.220>.

Jin, H. O., Y. H. Lee, J. A. Park, H. N. Lee, J. H. Kim, J. Y. Kim, B. Kim, S. E. Hong, H. A. Kim, E. K. Kim, W. C. Noh, J. I. Kim, Y. H. Chang, S. I. Hong, Y. J. Hong, I. C. Park, and J. K. Lee. 2014. "Piperlongumine induces cell death through ROS-mediated CHOP activation and potentiates TRAIL-induced cell death in breast cancer cells." *J Cancer Res Clin Oncol* 140 (12): 2039-46. <https://doi.org/10.1007/s00432-014-1777-1>.

Jin, S., Q. Y. Zhang, X. M. Kang, J. X. Wang, and W. H. Zhao. 2010. "Daidzein induces MCF-7 breast cancer cell apoptosis via the mitochondrial pathway." *Ann Oncol* 21 (2): 263-268. <https://doi.org/10.1093/annonc/mdp499>.

Kajimoto, S., N. Takanashi, T. Kajimoto, M. Xu, J. Cao, Y. Masuda, T. Aiuchi, S. Nakajo, Y. Ida, and K. Nakaya. 2002. "Sophoranone, extracted from a traditional Chinese medicine Shan Dou Gen, induces apoptosis in human leukemia U937 cells via formation of reactive oxygen species and opening of mitochondrial permeability transition pores." *Int J Cancer* 99 (6): 879-90. <https://doi.org/10.1002/ijc.10414>.

Kaleem, S., S. Siddiqui, H. H. Siddiqui, Badruddeen, A. Hussain, M. Arshad, J. Akhtar, and A. Rizvi. 2016. "Eupalitin induces apoptosis in prostate carcinoma cells through ROS generation and increase of caspase-3 activity." *Cell Biol Int* 40 (2): 196-203. <https://doi.org/10.1002/cbin.10552>.

Kang, X., H. Wang, Y. Li, Y. Xiao, L. Zhao, T. Zhang, S. Zhou, X. Zhou, Y. Li, Z. Shou, C. Chen, and B. Li. 2019. "Alantolactone induces apoptosis through ROS-mediated AKT pathway and inhibition of PINK1-mediated mitophagy in human HepG2 cells." *Artif Cells Nanomed Biotechnol* 47 (1): 1961-1970. <https://doi.org/10.1080/21691401.2019.1593854>.

Kang, Yufu, Jingchao Li, Li Jing, Yanling Zhang, and Xiangbo Wang. 2021. "Antiproliferative and apoptosis inducing effect of delphinidin against human bladder cancer cell line." *Pharmacognosy Magazine* 17: 101. <https://doi.org/10.4103/pm.pm_548_19>.

Kasiappan, R., I. Jutooru, K. Karki, E. Hedrick, and S. Safe. 2016. "Benzyl Isothiocyanate (BITC) Induces Reactive Oxygen Species-dependent Repression of STAT3 Protein by Down-regulation of Specificity Proteins in Pancreatic Cancer." *J Biol Chem* 291 (53): 27122-27133. <https://doi.org/10.1074/jbc.M116.746339>.

Kaufman, P. B., J. A. Duke, H. Brielmann, J. Boik, and J. E. Hoyt. 1997. "A comparative survey of leguminous plants as sources of the isoflavones, genistein and daidzein: implications for human nutrition and health." *J Altern Complement Med* 3 (1): 7-12. <https://doi.org/10.1089/acm.1997.3.7>.

Khaket, T. P., M. P. Singh, I. Khan, and S. C. Kang. 2020. "In vitro and in vivo studies on potentiation of curcumin-induced lysosomal-dependent apoptosis upon silencing of cathepsin C in colorectal cancer cells." *Pharmacol Res* 161: 105156. <https://doi.org/10.1016/j.phrs.2020.105156>.

Khan, F., I. Khan, A. Farooqui, and I. A. Ansari. 2017. "Carvacrol Induces Reactive Oxygen Species (ROS)-mediated Apoptosis Along with Cell Cycle Arrest at G(0)/G(1) in Human Prostate Cancer Cells." *Nutr Cancer* 69 (7): 1075-1087. <https://doi.org/10.1080/01635581.2017.1359321>.

Khan, F., V. K. Singh, M. Saeed, M. A. Kausar, and I. A. Ansari. 2019. "Carvacrol Induced Program Cell Death and Cell Cycle Arrest in Androgen-Independent Human Prostate Cancer Cells via Inhibition of Notch Signaling." *Anticancer Agents Med Chem* 19 (13): 1588-1608. <https://doi.org/10.2174/1871520619666190731152942>.

Khan, I., A. Bahuguna, P. Kumar, V. K. Bajpai, and S. C. Kang. 2018. "In vitro and in vivo antitumor potential of carvacrol nanoemulsion against human lung adenocarcinoma A549 cells via mitochondrial mediated apoptosis." *Sci Rep* 8 (1): 144. <https://doi.org/10.1038/s41598-017-18644-9>.

Khan, M., C. Ding, A. Rasul, F. Yi, T. Li, H. Gao, R. Gao, L. Zhong, K. Zhang, X. Fang, and T. Ma. 2012. "Isoalantolactone induces reactive oxygen species mediated apoptosis in pancreatic carcinoma PANC-1 cells." *Int J Biol Sci* 8 (4): 533-47. <https://doi.org/10.7150/ijbs.3753>.

Khan, M., F. Yi, A. Rasul, T. Li, N. Wang, H. Gao, R. Gao, and T. Ma. 2012. "Alantolactone induces apoptosis in glioblastoma cells via GSH depletion, ROS generation, and mitochondrial dysfunction." *IUBMB Life* 64 (9): 783-94. <https://doi.org/10.1002/iub.1068>.

Kim, B., J. H. Seo, K. Y. Lee, and B. Park. 2020. "Icariin sensitizes human colon cancer cells to TRAIL‑induced apoptosis via ERK‑mediated upregulation of death receptors." *Int J Oncol* 56 (3): 821-834. <https://doi.org/10.3892/ijo.2020.4970>.

Kim, C. S., W. H. Park, J. Y. Park, J. H. Kang, M. O. Kim, T. Kawada, H. Yoo, I. S. Han, and R. Yu. 2004. "Capsaicin, a spicy component of hot pepper, induces apoptosis by activation of the peroxisome proliferator-activated receptor gamma in HT-29 human colon cancer cells." *J Med Food* 7 (3): 267-73. <https://doi.org/10.1089/jmf.2004.7.267>.

Kim, D. H., K. W. Park, I. G. Chae, J. Kundu, E. H. Kim, J. K. Kundu, and K. S. Chun. 2016. "Carnosic acid inhibits STAT3 signaling and induces apoptosis through generation of ROS in human colon cancer HCT116 cells." *Mol Carcinog* 55 (6): 1096-110. <https://doi.org/10.1002/mc.22353>.

Kim, G. D. 2021. "Harmine Hydrochloride Triggers G2/M Cell Cycle Arrest and Apoptosis in HCT116 Cells through ERK and PI3K/AKT/mTOR Signaling Pathways." *Prev Nutr Food Sci* 26 (4): 445-452. <https://doi.org/10.3746/pnf.2021.26.4.445>.

Kim, H. S., H. N. Oh, A. W. Kwak, E. Kim, M. H. Lee, J. H. Seo, S. S. Cho, G. Yoon, J. I. Chae, and J. H. Shim. 2021. "Deoxypodophyllotoxin Inhibits Cell Growth and Induces Apoptosis by Blocking EGFR and MET in Gefitinib-Resistant Non-Small Cell Lung Cancer." *J Microbiol Biotechnol* 31 (4): 559-569. <https://doi.org/10.4014/jmb.2101.01029>.

Kim, M. Y., L. J. Trudel, and G. N. Wogan. 2009. "Apoptosis induced by capsaicin and resveratrol in colon carcinoma cells requires nitric oxide production and caspase activation." *Anticancer Res* 29 (10): 3733-40.

Kim, T. H., J. H. Park, and J. S. Woo. 2019. "Resveratrol induces cell death through ROS‑dependent downregulation of Notch1/PTEN/Akt signaling in ovarian cancer cells." *Mol Med Rep* 19 (4): 3353-3360. <https://doi.org/10.3892/mmr.2019.9962>.

Knickle, A., W. Fernando, A. L. Greenshields, H. P. V. Rupasinghe, and D. W. Hoskin. 2018. "Myricetin-induced apoptosis of triple-negative breast cancer cells is mediated by the iron-dependent generation of reactive oxygen species from hydrogen peroxide." *Food Chem Toxicol* 118: 154-167. <https://doi.org/10.1016/j.fct.2018.05.005>.

Kong, L., X. Wang, K. Zhang, W. Yuan, Q. Yang, J. Fan, P. Wang, and Q. Liu. 2015. "Gypenosides Synergistically Enhances the Anti-Tumor Effect of 5-Fluorouracil on Colorectal Cancer In Vitro and In Vivo: A Role for Oxidative Stress-Mediated DNA Damage and p53 Activation." *PLoS One* 10 (9): e0137888. <https://doi.org/10.1371/journal.pone.0137888>.

Kumar, A., D. Fan, D. J. Dipette, and U. S. Singh. 2014. "Sparstolonin B, a novel plant derived compound, arrests cell cycle and induces apoptosis in N-myc amplified and N-myc nonamplified neuroblastoma cells." *PLoS One* 9 (5): e96343. <https://doi.org/10.1371/journal.pone.0096343>.

Kumar, A., B. Singh, P. R. Sharma, S. B. Bharate, A. K. Saxena, and D. M. Mondhe. 2016. "A novel microtubule depolymerizing colchicine analogue triggers apoptosis and autophagy in HCT-116 colon cancer cells." *Cell Biochem Funct* 34 (2): 69-81. <https://doi.org/10.1002/cbf.3166>.

Kumar, V., and S. S. Chauhan. 2021. "Daidzein Induces Intrinsic Pathway of Apoptosis along with ER α/β Ratio Alteration and ROS Production." *Asian Pac J Cancer Prev* 22 (2): 603-610. <https://doi.org/10.31557/apjcp.2021.22.2.603>.

Kung, F. P., Y. P. Lim, W. Y. Chao, Y. S. Zhang, H. I. Yu, T. S. Tai, C. H. Lu, S. H. Chen, Y. Z. Li, P. W. Zhao, Y. P. Yen, and Y. R. Lee. 2021. "Piperlongumine, a Potent Anticancer Phytotherapeutic, Induces Cell Cycle Arrest and Apoptosis In Vitro and In Vivo through the ROS/Akt Pathway in Human Thyroid Cancer Cells." *Cancers (Basel)* 13 (17). <https://doi.org/10.3390/cancers13174266>.

Kwak, A. W., G. Yoon, M. H. Lee, S. S. Cho, J. H. Shim, and J. I. Chae. 2020. "Picropodophyllotoxin, an Epimer of Podophyllotoxin, Causes Apoptosis of Human Esophageal Squamous Cell Carcinoma Cells Through ROS-Mediated JNK/P38 MAPK Pathways." *Int J Mol Sci* 21 (13). <https://doi.org/10.3390/ijms21134640>.

Lee, D. H., J. G. Rhee, and Y. J. Lee. 2009. "Reactive oxygen species up-regulate p53 and Puma; a possible mechanism for apoptosis during combined treatment with TRAIL and wogonin." *Br J Pharmacol* 157 (7): 1189-202. <https://doi.org/10.1111/j.1476-5381.2009.00245.x>.

Lee, D. Y., Y. J. Park, S. C. Hwang, K. D. Kim, D. K. Moon, and D. H. Kim. 2018. "Cytotoxic effects of delphinidin in human osteosarcoma cells." *Acta Orthop Traumatol Turc* 52 (1): 58-64. <https://doi.org/10.1016/j.aott.2017.11.011>.

Lee, E. B., M. G. Cheon, J. Cui, Y. J. Lee, E. K. Seo, and H. H. Jang. 2017. "The quinone-based derivative, HMNQ induces apoptotic and autophagic cell death by modulating reactive oxygen species in cancer cells." *Oncotarget* 8 (59): 99637-99648. <https://doi.org/10.18632/oncotarget.21005>.

Lee, E. R., Y. J. Kang, H. J. Kim, H. Y. Choi, G. H. Kang, J. H. Kim, B. W. Kim, H. S. Jeong, Y. S. Park, and S. G. Cho. 2008. "Regulation of apoptosis by modified naringenin derivatives in human colorectal carcinoma RKO cells." *J Cell Biochem* 104 (1): 259-73. <https://doi.org/10.1002/jcb.21622>.

Lee, J. H., Y. C. Li, S. W. Ip, S. C. Hsu, N. W. Chang, N. Y. Tang, C. S. Yu, S. T. Chou, S. S. Lin, C. C. Lino, J. S. Yang, and J. G. Chung. 2008. "The role of Ca2+ in baicalein-induced apoptosis in human breast MDA-MB-231 cancer cells through mitochondria- and caspase-3-dependent pathway." *Anticancer Res* 28 (3a): 1701-11.

Lee, J. H., J. H. Yeon, H. Kim, W. Roh, J. Chae, H. O. Park, and D. M. Kim. 2012. "The natural anticancer agent plumbagin induces potent cytotoxicity in MCF-7 human breast cancer cells by inhibiting a PI-5 kinase for ROS generation." *PLoS One* 7 (9): e45023. <https://doi.org/10.1371/journal.pone.0045023>.

Lee, J. Y., B. Y. Kang, S. J. Jung, A. W. Kwak, S. O. Lee, J. W. Park, S. H. Joo, G. Yoon, M. H. Lee, and J. H. Shim. 2022. "Picropodophyllotoxin Inhibits Cell Growth and Induces Apoptosis in Gefitinib-Resistant Non-Small Lung Cancer Cells by Dual-Targeting EGFR and MET." *Biomol Ther (Seoul)*. <https://doi.org/10.4062/biomolther.2022.113>.

Lee, M. G., K. T. Lee, S. G. Chi, and J. H. Park. 2001. "Costunolide induces apoptosis by ROS-mediated mitochondrial permeability transition and cytochrome C release." *Biol Pharm Bull* 24 (3): 303-6. <https://doi.org/10.1248/bpb.24.303>.

Lee, S. O., A. W. Kwak, M. H. Lee, J. H. Seo, S. S. Cho, G. Yoon, J. I. Chae, S. H. Joo, and J. H. Shim. 2021. "Picropodophyllotoxin Induces G1 Cell Cycle Arrest and Apoptosis in Human Colorectal Cancer Cells via ROS Generation and Activation of p38 MAPK Signaling Pathway." *J Microbiol Biotechnol* 31 (12): 1615-1623. <https://doi.org/10.4014/jmb.2109.09012>.

Lee, Y. J., K. S. Park, H. S. Nam, M. K. Cho, and S. H. Lee. 2020. "Apigenin causes necroptosis by inducing ROS accumulation, mitochondrial dysfunction, and ATP depletion in malignant mesothelioma cells." *Korean J Physiol Pharmacol* 24 (6): 493-502. <https://doi.org/10.4196/kjpp.2020.24.6.493>.

Li, B., M. Lu, X. X. Jiang, M. X. Pan, J. W. Mao, and M. Chen. 2017. "Inhibiting reactive oxygen species-dependent autophagy enhanced baicalein-induced apoptosis in oral squamous cell carcinoma." *J Nat Med* 71 (2): 433-441. <https://doi.org/10.1007/s11418-017-1076-7>.

Li, H., X. X. Meng, L. Zhang, B. J. Zhang, X. Y. Liu, W. W. Fu, H. S. Tan, Y. Z. Lao, and H. X. Xu. 2017. "Oblongifolin C and guttiferone K extracted from Garcinia yunnanensis fruit synergistically induce apoptosis in human colorectal cancer cells in vitro." *Acta Pharmacol Sin* 38 (2): 252-263. <https://doi.org/10.1038/aps.2016.101>.

Li, H. Y., J. Zhang, L. L. Sun, B. H. Li, H. L. Gao, T. Xie, N. Zhang, and Z. M. Ye. 2015. "Celastrol induces apoptosis and autophagy via the ROS/JNK signaling pathway in human osteosarcoma cells: an in vitro and in vivo study." *Cell Death Dis* 6 (1): e1604. <https://doi.org/10.1038/cddis.2014.543>.

Li, J., C. C. Sharkey, and M. R. King. 2015. "Piperlongumine and immune cytokine TRAIL synergize to promote tumor death." *Sci Rep* 5: 9987. <https://doi.org/10.1038/srep09987>.

Li, K., Q. Zheng, X. Chen, Y. Wang, D. Wang, and J. Wang. 2018. "Isobavachalcone Induces ROS-Mediated Apoptosis via Targeting Thioredoxin Reductase 1 in Human Prostate Cancer PC-3 Cells." *Oxid Med Cell Longev* 2018: 1915828. <https://doi.org/10.1155/2018/1915828>.

Li, M., L. H. Song, G. G. Yue, J. K. Lee, L. M. Zhao, L. Li, X. Zhou, S. K. Tsui, S. S. Ng, K. P. Fung, N. H. Tan, and C. B. Lau. 2017. "Bigelovin triggered apoptosis in colorectal cancer in vitro and in vivo via upregulating death receptor 5 and reactive oxidative species." *Sci Rep* 7: 42176. <https://doi.org/10.1038/srep42176>.

Li, S. J., S. J. Sun, J. Gao, and F. B. Sun. 2016. "Wogonin induces Beclin-1/PI3K and reactive oxygen species-mediated autophagy in human pancreatic cancer cells." *Oncol Lett* 12 (6): 5059-5067. <https://doi.org/10.3892/ol.2016.5367>.

Li, W., B. Hua, S. M. Saud, H. Lin, W. Hou, M. S. Matter, L. Jia, N. H. Colburn, and M. R. Young. 2015. "Berberine regulates AMP-activated protein kinase signaling pathways and inhibits colon tumorigenesis in mice." *Mol Carcinog* 54 (10): 1096-109. <https://doi.org/10.1002/mc.22179>.

Li, W., S. Nie, Q. Yu, and M. Xie. 2009. "(-)-Epigallocatechin-3-gallate induces apoptosis of human hepatoma cells by mitochondrial pathways related to reactive oxygen species." *J Agric Food Chem* 57 (15): 6685-91. <https://doi.org/10.1021/jf901396f>.

Li, X., H. Liu, C. Lv, J. Du, F. Lian, S. Zhang, Z. Wang, and Y. Zeng. 2022. "Gypenoside-Induced Apoptosis via the PI3K/AKT/mTOR Signaling Pathway in Bladder Cancer." *Biomed Res Int* 2022: 9304552. <https://doi.org/10.1155/2022/9304552>.

Li, Z., B. Qin, X. Qi, J. Mao, and D. Wu. 2016. "Isoalantolactone induces apoptosis in human breast cancer cells via ROS-mediated mitochondrial pathway and downregulation of SIRT1." *Arch Pharm Res* 39 (10): 1441-1453. <https://doi.org/10.1007/s12272-016-0815-8>.

Li, Z., X. Zhou, H. Zhu, X. Song, H. Gao, Z. Niu, and J. Lu. 2021. "Purpurin binding interacts with LHPP protein that inhibits PI3K/AKT phosphorylation and induces apoptosis in colon cancer cells HCT-116." *J Biochem Mol Toxicol* 35 (3): e22665. <https://doi.org/10.1002/jbt.22665>.

Liang, L., J. Wu, J. Luo, L. Wang, Z. X. Chen, C. L. Han, T. Q. Gan, J. A. Huang, and Z. W. Cai. 2020. "Oxymatrine reverses 5-fluorouracil resistance by inhibition of colon cancer cell epithelial-mesenchymal transition and NF-κB signaling in vitro." *Oncol Lett* 19 (1): 519-526. <https://doi.org/10.3892/ol.2019.11090>.

Liang, L., and Z. Zhang. 2016. "Gambogic Acid Inhibits Malignant Melanoma Cell Proliferation Through Mitochondrial p66shc/ROS-p53/Bax-Mediated Apoptosis." *Cell Physiol Biochem* 38 (4): 1618-30. <https://doi.org/10.1159/000443102>.

Liang, W., A. Cai, G. Chen, H. Xi, X. Wu, J. Cui, K. Zhang, X. Zhao, J. Yu, B. Wei, and L. Chen. 2016. "Shikonin induces mitochondria-mediated apoptosis and enhances chemotherapeutic sensitivity of gastric cancer through reactive oxygen species." *Sci Rep* 6: 38267. <https://doi.org/10.1038/srep38267>.

Liang, X., P. Wang, C. Yang, F. Huang, H. Wu, H. Shi, and X. Wu. 2021. "Galangin Inhibits Gastric Cancer Growth Through Enhancing STAT3 Mediated ROS Production." *Front Pharmacol* 12: 646628. <https://doi.org/10.3389/fphar.2021.646628>.

Lim, H. M., J. Lee, M. J. Nam, and S. H. Park. 2021. "Acetylshikonin Induces Apoptosis in Human Colorectal Cancer HCT-15 and LoVo Cells via Nuclear Translocation of FOXO3 and ROS Level Elevation." *Oxid Med Cell Longev* 2021: 6647107. <https://doi.org/10.1155/2021/6647107>.

Lim, H. M., J. Lee, S. H. Yu, M. J. Nam, H. S. Cha, K. Park, Y. H. Yang, K. Y. Jang, and S. H. Park. 2022. "Acetylshikonin, A Novel CYP2J2 Inhibitor, Induces Apoptosis in RCC Cells via FOXO3 Activation and ROS Elevation." *Oxid Med Cell Longev* 2022: 9139338. <https://doi.org/10.1155/2022/9139338>.

Lim, S. C., H. J. Jeon, K. H. Kee, M. J. Lee, R. Hong, and S. I. Han. 2017. "Andrographolide induces apoptotic and non-apoptotic death and enhances tumor necrosis factor-related apoptosis-inducing ligand-mediated apoptosis in gastric cancer cells." *Oncol Lett* 13 (5): 3837-3844. <https://doi.org/10.3892/ol.2017.5923>.

Lim, W., J. Ham, F. W. Bazer, and G. Song. 2019. "Carvacrol induces mitochondria-mediated apoptosis via disruption of calcium homeostasis in human choriocarcinoma cells." *J Cell Physiol* 234 (2): 1803-1815. <https://doi.org/10.1002/jcp.27054>.

Lin, C. J., T. L. Chen, Y. Y. Tseng, G. J. Wu, M. H. Hsieh, Y. W. Lin, and R. M. Chen. 2016. "Honokiol induces autophagic cell death in malignant glioma through reactive oxygen species-mediated regulation of the p53/PI3K/Akt/mTOR signaling pathway." *Toxicol Appl Pharmacol* 304: 59-69. <https://doi.org/10.1016/j.taap.2016.05.018>.

Lin, Y. J., S. F. Peng, M. L. Lin, C. L. Kuo, K. W. Lu, C. L. Liao, Y. S. Ma, F. S. Chueh, K. C. Liu, F. S. Yu, and J. G. Chung. 2016. "Tetrandrine Induces Apoptosis of Human Nasopharyngeal Carcinoma NPC-TW 076 Cells through Reactive Oxygen Species Accompanied by an Endoplasmic Reticulum Stress Signaling Pathway." *Molecules* 21 (10). <https://doi.org/10.3390/molecules21101353>.

Lin, Y., Q. Zhang, B. Xie, H. Jiang, J. Shen, S. Tang, and C. Dai. 2022. "Chelerythrine-Induced Apoptotic Cell Death in HepG2 Cells Involves the Inhibition of Akt Pathway and the Activation of Oxidative Stress and Mitochondrial Apoptotic Pathway." *Antioxidants (Basel)* 11 (9). <https://doi.org/10.3390/antiox11091837>.

Liu, C., K. Gong, X. Mao, and W. Li. 2011. "Tetrandrine induces apoptosis by activating reactive oxygen species and repressing Akt activity in human hepatocellular carcinoma." *Int J Cancer* 129 (6): 1519-31. <https://doi.org/10.1002/ijc.25817>.

Liu, J., S. Fan, Y. Xiang, J. Xia, H. Jin, J. F. Xu, F. Yang, J. Cai, and J. Pi. 2022. "Nanoscale Features of Gambogic Acid Induced ROS-Dependent Apoptosis in Esophageal Cancer Cells Imaged by Atomic Force Microscopy." *Scanning* 2022: 1422185. <https://doi.org/10.1155/2022/1422185>.

Liu, J., Q. Li, Z. Liu, L. Lin, X. Zhang, M. Cao, and J. Jiang. 2016. "Harmine induces cell cycle arrest and mitochondrial pathway-mediated cellular apoptosis in SW620 cells via inhibition of the Akt and ERK signaling pathways." *Oncol Rep* 35 (6): 3363-70. <https://doi.org/10.3892/or.2016.4695>.

Liu, L. H., Y. J. Zhou, L. Ding, S. Z. Zhang, J. Sun, and J. G. Cao. 2014. "Induction of apoptosis by VB1 in breast cancer cells: the role of reactive oxygen species and Bcl-2 family proteins." *Int J Mol Med* 33 (2): 423-30. <https://doi.org/10.3892/ijmm.2013.1567>.

Liu, L., X. Sun, Y. Guo, and K. Ge. 2022. "Evodiamine induces ROS-Dependent cytotoxicity in human gastric cancer cells via TRPV1/Ca(2+) pathway." *Chem Biol Interact* 351: 109756. <https://doi.org/10.1016/j.cbi.2021.109756>.

Liu, N., Y. Li, G. Chen, and K. Ge. 2020. "Evodiamine induces reactive oxygen species-dependent apoptosis and necroptosis in human melanoma A-375 cells." *Oncol Lett* 20 (4): 121. <https://doi.org/10.3892/ol.2020.11983>.

Liu, S., J. Hu, C. Shi, L. Sun, W. Yan, and Y. Song. 2021. "Sparstolonin B exerts beneficial effects on prostate cancer by acting on the reactive oxygen species-mediated PI3K/AKT pathway." *J Cell Mol Med* 25 (12): 5511-5524. <https://doi.org/10.1111/jcmm.16560>.

Liu, T., X. Liu, and W. Li. 2016. "Tetrandrine, a Chinese plant-derived alkaloid, is a potential candidate for cancer chemotherapy." *Oncotarget* 7 (26): 40800-40815. <https://doi.org/10.18632/oncotarget.8315>.

Liu, X., Q. Ji, N. Ye, H. Sui, L. Zhou, H. Zhu, Z. Fan, J. Cai, and Q. Li. 2015. "Berberine Inhibits Invasion and Metastasis of Colorectal Cancer Cells via COX-2/PGE2 Mediated JAK2/STAT3 Signaling Pathway." *PLoS One* 10 (5): e0123478. <https://doi.org/10.1371/journal.pone.0123478>.

Liu, X., Y. Zhang, S. Wu, M. Xu, Y. Shen, M. Yu, J. Fan, S. Wei, C. Xu, L. Huang, H. Zhao, X. Li, and X. Ye. 2020. "Palmatine induces G2/M phase arrest and mitochondrial-associated pathway apoptosis in colon cancer cells by targeting AURKA." *Biochem Pharmacol* 175: 113933. <https://doi.org/10.1016/j.bcp.2020.113933>.

Liu, Y. B., X. Gao, D. Deeb, A. S. Arbab, and S. C. Gautam. 2013. "Pristimerin Induces Apoptosis in Prostate Cancer Cells by Down-regulating Bcl-2 through ROS-dependent Ubiquitin-proteasomal Degradation Pathway." *J Carcinog Mutagen* Suppl 6: 005. <https://doi.org/10.4172/2157-2518.S6-005>.

Liu, Z. H., C. X. Yang, L. Zhang, C. Y. Yang, and X. Q. Xu. 2019. "Baicalein, as a Prooxidant, Triggers Mitochondrial Apoptosis in MCF-7 Human Breast Cancer Cells Through Mobilization of Intracellular Copper and Reactive Oxygen Species Generation." *Onco Targets Ther* 12: 10749-10761. <https://doi.org/10.2147/ott.S222819>.

Lopes, T. Z., F. R. de Moraes, A. C. Tedesco, R. K. Arni, P. Rahal, and M. F. Calmon. 2020. "Berberine associated photodynamic therapy promotes autophagy and apoptosis via ROS generation in renal carcinoma cells." *Biomed Pharmacother* 123: 109794. <https://doi.org/10.1016/j.biopha.2019.109794>.

Lu, H. F., Y. L. Chen, J. S. Yang, Y. Y. Yang, J. Y. Liu, S. C. Hsu, K. C. Lai, and J. G. Chung. 2010. "Antitumor activity of capsaicin on human colon cancer cells in vitro and colo 205 tumor xenografts in vivo." *J Agric Food Chem* 58 (24): 12999-3005. <https://doi.org/10.1021/jf103335w>.

Lv, L., W. Zhang, T. Li, L. Jiang, X. Lu, and J. Lin. 2020. "Hispidulin exhibits potent anticancer activity in vitro and in vivo through activating ER stress in non‑small‑cell lung cancer cells." *Oncol Rep* 43 (6): 1995-2003. <https://doi.org/10.3892/or.2020.7568>.

Lyu, L., L. Q. Huang, T. Huang, W. Xiang, J. D. Yuan, and C. H. Zhang. 2018. "Cell-penetrating peptide conjugates of gambogic acid enhance the antitumor effect on human bladder cancer EJ cells through ROS-mediated apoptosis." *Drug Des Devel Ther* 12: 743-756. <https://doi.org/10.2147/dddt.S161821>.

Manogaran, P., N. M. Beeraka, C. Y. Huang, and V. Vijaya Padma. 2019. "Neferine and isoliensinine enhance 'intracellular uptake of cisplatin' and induce 'ROS-mediated apoptosis' in colorectal cancer cells - A comparative study." *Food Chem Toxicol* 132: 110652. <https://doi.org/10.1016/j.fct.2019.110652>.

Manogaran, P., B. Somasundaram, and V. P. Viswanadha. 2022. "Reversal of cisplatin resistance by neferine/isoliensinine and their combinatorial regimens with cisplatin-induced apoptosis in cisplatin-resistant colon cancer stem cells (CSCs)." *J Biochem Mol Toxicol* 36 (3): e22967. <https://doi.org/10.1002/jbt.22967>.

Mehmood, T., A. Maryam, H. Zhang, Y. Li, M. Khan, and T. Ma. 2017. "Deoxyelephantopin induces apoptosis in HepG2 cells via oxidative stress, NF-κB inhibition and mitochondrial dysfunction." *Biofactors* 43 (1): 63-72. <https://doi.org/10.1002/biof.1324>.

Meng, L. H., H. Zhang, L. Hayward, H. Takemura, R. G. Shao, and Y. Pommier. 2004. "Tetrandrine induces early G1 arrest in human colon carcinoma cells by down-regulating the activity and inducing the degradation of G1-S-specific cyclin-dependent kinases and by inducing p53 and p21Cip1." *Cancer Res* 64 (24): 9086-92. <https://doi.org/10.1158/0008-5472.Can-04-0313>.

Mhlanga, P., P. O. Perumal, A. M. Somboro, D. G. Amoako, H. M. Khumalo, and R. B. Khan. 2019. "Mechanistic Insights into Oxidative Stress and Apoptosis Mediated by Tannic Acid in Human Liver Hepatocellular Carcinoma Cells." *Int J Mol Sci* 20 (24). <https://doi.org/10.3390/ijms20246145>.

Mhone, T. G., M. C. Chen, C. H. Kuo, T. C. Shih, C. M. Yeh, T. F. Wang, R. J. Chen, Y. C. Chang, W. W. Kuo, and C. Y. Huang. 2022. "Daidzein Synergizes with Gefitinib to Induce ROS/JNK/c-Jun Activation and Inhibit EGFR-STAT/AKT/ERK Pathways to enhance Lung Adenocarcinoma cells chemosensitivity." *Int J Biol Sci* 18 (9): 3636-3652. <https://doi.org/10.7150/ijbs.71870>.

Min, K. J., J. O. Nam, and T. K. Kwon. 2017. "Fisetin Induces Apoptosis Through p53-Mediated Up-Regulation of DR5 Expression in Human Renal Carcinoma Caki Cells." *Molecules* 22 (8). <https://doi.org/10.3390/molecules22081285>.

Morrissey, C., A. O'Neill, B. Spengler, V. Christoffel, J. M. Fitzpatrick, and R. W. Watson. 2005. "Apigenin drives the production of reactive oxygen species and initiates a mitochondrial mediated cell death pathway in prostate epithelial cells." *Prostate* 63 (2): 131-42. <https://doi.org/10.1002/pros.20167>.

Nagesh, P. K. B., P. Chowdhury, E. Hatami, S. Jain, N. Dan, V. K. Kashyap, S. C. Chauhan, M. Jaggi, and M. M. Yallapu. 2020. "Tannic acid inhibits lipid metabolism and induce ROS in prostate cancer cells." *Sci Rep* 10 (1): 980. <https://doi.org/10.1038/s41598-020-57932-9>.

Namazi Sarvestani, N., H. Sepehri, L. Delphi, and M. Moridi Farimani. 2018. "Eupatorin and Salvigenin Potentiate Doxorubicin-Induced Apoptosis and Cell Cycle Arrest in HT-29 and SW948 Human Colon Cancer Cells." *Asian Pac J Cancer Prev* 19 (1): 131-139. <https://doi.org/10.22034/apjcp.2018.19.1.131>.

NavaneethaKrishnan, S., J. L. Rosales, and K. Y. Lee. 2019. "ROS-Mediated Cancer Cell Killing through Dietary Phytochemicals." *Oxid Med Cell Longev* 2019: 9051542. <https://doi.org/10.1155/2019/9051542>.

Nazeem, S., A. S. Azmi, S. Hanif, A. Ahmad, R. M. Mohammad, S. M. Hadi, and K. S. Kumar. 2009. "Plumbagin induces cell death through a copper-redox cycle mechanism in human cancer cells." *Mutagenesis* 24 (5): 413-8. <https://doi.org/10.1093/mutage/gep023>.

Nelson, V. K., C. P. Pullaiah, M. Saleem Ts, S. Roychoudhury, S. Chinnappan, B. Vishnusai, R. Ram Mani, G. Birudala, and K. S. Bottu. 2022. "Natural Products as the Modulators of Oxidative Stress: An Herbal Approach in the Management of Prostate Cancer." *Adv Exp Med Biol* 1391: 161-179. <https://doi.org/10.1007/978-3-031-12966-7_10>.

Nie, F., X. Zhang, Q. Qi, L. Yang, Y. Yang, W. Liu, N. Lu, Z. Wu, Q. You, and Q. Guo. 2009. "Reactive oxygen species accumulation contributes to gambogic acid-induced apoptosis in human hepatoma SMMC-7721 cells." *Toxicology* 260 (1-3): 60-7. <https://doi.org/10.1016/j.tox.2009.03.010>.

Niu, W., J. Wang, Q. Wang, and J. Shen. 2020. "Celastrol Loaded Nanoparticles With ROS-Response and ROS-Inducer for the Treatment of Ovarian Cancer." *Front Chem* 8: 574614. <https://doi.org/10.3389/fchem.2020.574614>.

Oh, H. N., M. H. Lee, E. Kim, A. W. Kwak, G. Yoon, S. S. Cho, K. Liu, J. I. Chae, and J. H. Shim. 2020. "Licochalcone D Induces ROS-Dependent Apoptosis in Gefitinib-Sensitive or Resistant Lung Cancer Cells by Targeting EGFR and MET." *Biomolecules* 10 (2). <https://doi.org/10.3390/biom10020297>.

Oh, H. N., M. H. Lee, E. Kim, G. Yoon, J. I. Chae, and J. H. Shim. 2019. "Licochalcone B inhibits growth and induces apoptosis of human non-small-cell lung cancer cells by dual targeting of EGFR and MET." *Phytomedicine* 63: 153014. <https://doi.org/10.1016/j.phymed.2019.153014>.

Ong, J. Y., P. V. Yong, Y. M. Lim, and A. S. Ho. 2015. "2-Methoxy-1,4-naphthoquinone (MNQ) induces apoptosis of A549 lung adenocarcinoma cells via oxidation-triggered JNK and p38 MAPK signaling pathways." *Life Sci* 135: 158-64. <https://doi.org/10.1016/j.lfs.2015.03.019>.

Palit, S., S. Kar, G. Sharma, and P. K. Das. 2015. "Hesperetin Induces Apoptosis in Breast Carcinoma by Triggering Accumulation of ROS and Activation of ASK1/JNK Pathway." *J Cell Physiol* 230 (8): 1729-39. <https://doi.org/10.1002/jcp.24818>.

Pan, D., W. Zhang, N. Zhang, Y. Xu, Y. Chen, J. Peng, Y. Chen, Y. Zhang, and X. Shen. 2021. "Oxymatrine Synergistically Enhances Doxorubicin Anticancer Effects in Colorectal Cancer." *Front Pharmacol* 12: 673432. <https://doi.org/10.3389/fphar.2021.673432>.

Pan, M. H., C. S. Lai, P. C. Hsu, and Y. J. Wang. 2005. "Acacetin induces apoptosis in human gastric carcinoma cells accompanied by activation of caspase cascades and production of reactive oxygen species." *J Agric Food Chem* 53 (3): 620-30. <https://doi.org/10.1021/jf048430m>.

Pandey, K., S. K. Tripathi, M. Panda, and B. K. Biswal. 2020. "Prooxidative activity of plumbagin induces apoptosis in human pancreatic ductal adenocarcinoma cells via intrinsic apoptotic pathway." *Toxicol In Vitro* 65: 104788. <https://doi.org/10.1016/j.tiv.2020.104788>.

Pandey, N., G. Tyagi, P. Kaur, S. Pradhan, M. V. Rajam, and T. Srivastava. 2020. "Allicin Overcomes Hypoxia Mediated Cisplatin Resistance in Lung Cancer Cells through ROS Mediated Cell Death Pathway and by Suppressing Hypoxia Inducible Factors." *Cell Physiol Biochem* 54 (4): 748-766. <https://doi.org/10.33594/000000253>.

Pang, H., T. Wu, Z. Peng, Q. Tan, X. Peng, Z. Zhan, L. Song, and B. Wei. 2022. "Baicalin induces apoptosis and autophagy in human osteosarcoma cells by increasing ROS to inhibit PI3K/Akt/mTOR, ERK1/2 and β-catenin signaling pathways." *J Bone Oncol* 33: 100415. <https://doi.org/10.1016/j.jbo.2022.100415>.

Park, J. J., S. M. Seo, E. J. Kim, Y. J. Lee, Y. G. Ko, J. Ha, and M. Lee. 2012. "Berberine inhibits human colon cancer cell migration via AMP-activated protein kinase-mediated downregulation of integrin β1 signaling." *Biochem Biophys Res Commun* 426 (4): 461-7. <https://doi.org/10.1016/j.bbrc.2012.08.091>.

Park, S., J. Oh, M. Kim, and E. J. Jin. 2018. "Bromelain effectively suppresses Kras-mutant colorectal cancer by stimulating ferroptosis." *Anim Cells Syst (Seoul)* 22 (5): 334-340. <https://doi.org/10.1080/19768354.2018.1512521>.

Pereyra-Vergara, F., I. M. Olivares-Corichi, A. G. Perez-Ruiz, J. P. Luna-Arias, and J. R. García-Sánchez. 2020. "Apoptosis Induced by (-)-Epicatechin in Human Breast Cancer Cells is Mediated by Reactive Oxygen Species." *Molecules* 25 (5). <https://doi.org/10.3390/molecules25051020>.

Pibiri, M., P. Sulas, T. Camboni, V. P. Leoni, and G. Simbula. 2020. "α-Lipoic acid induces Endoplasmic Reticulum stress-mediated apoptosis in hepatoma cells." *Sci Rep* 10 (1): 7139. <https://doi.org/10.1038/s41598-020-64004-5>.

Poornima, P., R. S. Quency, and V. V. Padma. 2013. "Neferine induces reactive oxygen species mediated intrinsic pathway of apoptosis in HepG2 cells." *Food Chem* 136 (2): 659-67. <https://doi.org/10.1016/j.foodchem.2012.07.112>.

Pramanik, K. C., S. R. Boreddy, and S. K. Srivastava. 2011. "Role of mitochondrial electron transport chain complexes in capsaicin mediated oxidative stress leading to apoptosis in pancreatic cancer cells." *PLoS One* 6 (5): e20151. <https://doi.org/10.1371/journal.pone.0020151>.

Prasad, N., J. R. Sharma, and U. C. S. Yadav. 2020. "Induction of growth cessation by acacetin via β-catenin pathway and apoptosis by apoptosis inducing factor activation in colorectal carcinoma cells." *Mol Biol Rep* 47 (2): 987-1001. <https://doi.org/10.1007/s11033-019-05191-x>.

Pushpalatha, Rajangam, Ganapathy Sindhu, Ramalingam Sharmila, G. Tamizharasi, Lakshmanan Vennila, and Annamalai Vijayalakshmi. 2021. "Linalool Induces Reactive Oxygen Species Mediated Apoptosis in Human Oral Squamous Carcinoma Cells." *Indian Journal of Pharmaceutical Sciences* 83. <https://doi.org/10.36468/pharmaceutical-sciences.842>.

Qi, H., X. Zhang, H. Liu, M. Han, X. Tang, S. Qu, X. Wang, and Y. Yang. 2022. "Shikonin induced Apoptosis Mediated by Endoplasmic Reticulum Stress in Colorectal Cancer Cells." *J Cancer* 13 (1): 243-252. <https://doi.org/10.7150/jca.65297>.

Qian, C., Y. Wang, Y. Zhong, J. Tang, J. Zhang, Z. Li, Q. Wang, and R. Hu. 2014. "Wogonin-enhanced reactive oxygen species-induced apoptosis and potentiated cytotoxic effects of chemotherapeutic agents by suppression Nrf2-mediated signaling in HepG2 cells." *Free Radic Res* 48 (5): 607-21. <https://doi.org/10.3109/10715762.2014.897342>.

Qiao, C., N. Lu, Y. Zhou, T. Ni, Y. Dai, Z. Li, Q. Guo, and L. Wei. 2016. "Oroxylin A modulates mitochondrial function and apoptosis in human colon cancer cells by inducing mitochondrial translocation of wild-type p53." *Oncotarget* 7 (13): 17009-20. <https://doi.org/10.18632/oncotarget.7927>.

Qiao, Z., Y. Cheng, S. Liu, Z. Ma, S. Li, and W. Zhang. 2019. "Casticin inhibits esophageal cancer cell proliferation and promotes apoptosis by regulating mitochondrial apoptotic and JNK signaling pathways." *Naunyn Schmiedebergs Arch Pharmacol* 392 (2): 177-187. <https://doi.org/10.1007/s00210-018-1574-5>.

Qin, R., H. Shen, Y. Cao, Y. Fang, H. Li, Q. Chen, and W. Xu. 2013. "Tetrandrine induces mitochondria-mediated apoptosis in human gastric cancer BGC-823 cells." *PLoS One* 8 (10): e76486. <https://doi.org/10.1371/journal.pone.0076486>.

Rajendran, P., U. Maheshwari, A. Muthukrishnan, R. Muthuswamy, K. Anand, B. Ravindran, P. Dhanaraj, B. Balamuralikrishnan, S. W. Chang, and W. J. Chung. 2021. "Myricetin: versatile plant based flavonoid for cancer treatment by inducing cell cycle arrest and ROS-reliant mitochondria-facilitated apoptosis in A549 lung cancer cells and in silico prediction." *Mol Cell Biochem* 476 (1): 57-68. <https://doi.org/10.1007/s11010-020-03885-6>.

Rampogu, S., R. G. Gajula, and K. W. Lee. 2021. "A comprehensive review on chemotherapeutic potential of galangin." *Biomed Pharmacother* 141: 111808. <https://doi.org/10.1016/j.biopha.2021.111808>.

Rasul, A., R. Bao, M. Malhi, B. Zhao, I. Tsuji, J. Li, and X. Li. 2013. "Induction of apoptosis by costunolide in bladder cancer cells is mediated through ROS generation and mitochondrial dysfunction." *Molecules* 18 (2): 1418-33. <https://doi.org/10.3390/molecules18021418>.

Rasul, A., J. Di, F. M. Millimouno, M. Malhi, I. Tsuji, M. Ali, J. Li, and X. Li. 2013. "Reactive oxygen species mediate isoalantolactone-induced apoptosis in human prostate cancer cells." *Molecules* 18 (8): 9382-96. <https://doi.org/10.3390/molecules18089382>.

Rattanawong, A., V. Payon, W. Limpanasittikul, C. Boonkrai, A. Mutirangura, and P. Wonganan. 2018. "Cepharanthine exhibits a potent anticancer activity in p53-mutated colorectal cancer cells through upregulation of p21Waf1/Cip1." *Oncol Rep* 39 (1): 227-238. <https://doi.org/10.3892/or.2017.6084>.

Rawat, L., H. Hegde, S. L. Hoti, and V. Nayak. 2020. "Piperlongumine induces ROS mediated cell death and synergizes paclitaxel in human intestinal cancer cells." *Biomed Pharmacother* 128: 110243. <https://doi.org/10.1016/j.biopha.2020.110243>.

Rawat, L., and V. Nayak. 2022. "Piperlongumine induces ROS mediated apoptosis by transcriptional regulation of SMAD4/P21/P53 genes and synergizes with doxorubicin in osteosarcoma cells." *Chem Biol Interact* 354: 109832. <https://doi.org/10.1016/j.cbi.2022.109832>.

Raymond, E., C. Louvet, C. Tournigand, A. M. Coudray, S. Faivre, A. De Gramont, and C. Gespach. 2002. "Pemetrexed disodium combined with oxaliplatin, SN38, or 5-fluorouracil, based on the quantitation of drug interactions in human HT29 colon cancer cells." *Int J Oncol* 21 (2): 361-7.

Ren, G., T. Sha, J. Guo, W. Li, J. Lu, and X. Chen. 2015. "Cucurbitacin B induces DNA damage and autophagy mediated by reactive oxygen species (ROS) in MCF-7 breast cancer cells." *J Nat Med* 69 (4): 522-30. <https://doi.org/10.1007/s11418-015-0918-4>.

Ren, J., J. Yang, Y. Xu, Q. Huang, M. Yang, and K. Hu. 2015. "Lupiwighteone induces cell cycle arrest and apoptosis and activates the Nrf2/ARE pathway in human neuroblastoma cells." *Biomed Pharmacother* 69: 153-61. <https://doi.org/10.1016/j.biopha.2014.11.016>.

Rosa, L. S., N. A. Jordão, N. da Costa Pereira Soares, J. F. deMesquita, M. Monteiro, and A. J. Teodoro. 2018. "Pharmacokinetic, Antiproliferative and Apoptotic Effects of Phenolic Acids in Human Colon Adenocarcinoma Cells Using In Vitro and In Silico Approaches." *Molecules* 23 (10). <https://doi.org/10.3390/molecules23102569>.

Russell, L. H., Jr., E. Mazzio, R. B. Badisa, Z. P. Zhu, M. Agharahimi, E. T. Oriaku, and C. B. Goodman. 2012. "Autoxidation of gallic acid induces ROS-dependent death in human prostate cancer LNCaP cells." *Anticancer Res* 32 (5): 1595-602.

Ryu, S., W. Lim, F. W. Bazer, and G. Song. 2017. "Chrysin induces death of prostate cancer cells by inducing ROS and ER stress." *J Cell Physiol* 232 (12): 3786-3797. <https://doi.org/10.1002/jcp.25861>.

Sahu, R. P., R. Zhang, S. Batra, Y. Shi, and S. K. Srivastava. 2009. "Benzyl isothiocyanate-mediated generation of reactive oxygen species causes cell cycle arrest and induces apoptosis via activation of MAPK in human pancreatic cancer cells." *Carcinogenesis* 30 (10): 1744-53. <https://doi.org/10.1093/carcin/bgp157>.

Sampaio, L. A., L. T. S. Pina, M. R. Serafini, D. D. S. Tavares, and A. G. Guimarães. 2021. "Antitumor Effects of Carvacrol and Thymol: A Systematic Review." *Front Pharmacol* 12: 702487. <https://doi.org/10.3389/fphar.2021.702487>.

Santos, L. S., V. R. Silva, L. R. A. Menezes, M. B. P. Soares, E. V. Costa, and D. P. Bezerra. 2017. "Xylopine Induces Oxidative Stress and Causes G(2)/M Phase Arrest, Triggering Caspase-Mediated Apoptosis by p53-Independent Pathway in HCT116 Cells." *Oxid Med Cell Longev* 2017: 7126872. <https://doi.org/10.1155/2017/7126872>.

Seydi, E., H. R. Rasekh, A. Salimi, Z. Mohsenifar, and J. Pourahmad. 2016. "Myricetin Selectively Induces Apoptosis on Cancerous Hepatocytes by Directly Targeting Their Mitochondria." *Basic Clin Pharmacol Toxicol* 119 (3): 249-58. <https://doi.org/10.1111/bcpt.12572>.

Shendge, A. K., D. Chaudhuri, and N. Mandal. 2021. "The natural flavones, acacetin and apigenin, induce Cdk-Cyclin mediated G2/M phase arrest and trigger ROS-mediated apoptosis in glioblastoma cells." *Mol Biol Rep* 48 (1): 539-549. <https://doi.org/10.1007/s11033-020-06087-x>.

Shi, C. S., J. M. Li, C. C. Chin, Y. H. Kuo, Y. R. Lee, and Y. C. Huang. 2017. "Evodiamine Induces Cell Growth Arrest, Apoptosis and Suppresses Tumorigenesis in Human Urothelial Cell Carcinoma Cells." *Anticancer Res* 37 (3): 1149-1159. <https://doi.org/10.21873/anticanres.11428>.

Shi, J. M., L. L. Bai, D. M. Zhang, A. Yiu, Z. Q. Yin, W. L. Han, J. S. Liu, Y. Li, D. Y. Fu, and W. C. Ye. 2013. "Saxifragifolin D induces the interplay between apoptosis and autophagy in breast cancer cells through ROS-dependent endoplasmic reticulum stress." *Biochem Pharmacol* 85 (7): 913-26. <https://doi.org/10.1016/j.bcp.2013.01.009>.

Shukla, S., and S. Gupta. 2008. "Apigenin-induced prostate cancer cell death is initiated by reactive oxygen species and p53 activation." *Free Radic Biol Med* 44 (10): 1833-45. <https://doi.org/10.1016/j.freeradbiomed.2008.02.007>.

Singh, S. V., S. K. Srivastava, S. Choi, K. L. Lew, J. Antosiewicz, D. Xiao, Y. Zeng, S. C. Watkins, C. S. Johnson, D. L. Trump, Y. J. Lee, H. Xiao, and A. Herman-Antosiewicz. 2005. "Sulforaphane-induced cell death in human prostate cancer cells is initiated by reactive oxygen species." *J Biol Chem* 280 (20): 19911-24. <https://doi.org/10.1074/jbc.M412443200>.

Song, L., X. Chen, L. Mi, C. Liu, S. Zhu, T. Yang, X. Luo, Q. Zhang, H. Lu, and X. Liang. 2020. "Icariin-induced inhibition of SIRT6/NF-κB triggers redox mediated apoptosis and enhances anti-tumor immunity in triple-negative breast cancer." *Cancer Sci* 111 (11): 4242-4256. <https://doi.org/10.1111/cas.14648>.

Song, N., J. Ma, W. Hu, Y. Guo, L. Hui, M. Aamer, and J. Ma. 2021. "Lappaconitine hydrochloride inhibits proliferation and induces apoptosis in human colon cancer HCT-116 cells via mitochondrial and MAPK pathway." *Acta Histochem* 123 (5): 151736. <https://doi.org/10.1016/j.acthis.2021.151736>.

Song, Z., X. Xiang, J. Li, J. Deng, Z. Fang, L. Zhang, and J. Xiong. 2020. "Ruscogenin induces ferroptosis in pancreatic cancer cells." *Oncol Rep* 43 (2): 516-524. <https://doi.org/10.3892/or.2019.7425>.

Sp, N., D. Y. Kang, E. S. Jo, A. Rugamba, W. S. Kim, Y. M. Park, D. Y. Hwang, J. S. Yoo, Q. Liu, K. J. Jang, and Y. M. Yang. 2020. "Tannic Acid Promotes TRAIL-Induced Extrinsic Apoptosis by Regulating Mitochondrial ROS in Human Embryonic Carcinoma Cells." *Cells* 9 (2). <https://doi.org/10.3390/cells9020282>.

Srinivas, P., G. Gopinath, A. Banerji, A. Dinakar, and G. Srinivas. 2004. "Plumbagin induces reactive oxygen species, which mediate apoptosis in human cervical cancer cells." *Mol Carcinog* 40 (4): 201-11. <https://doi.org/10.1002/mc.20031>.

Su, C. H., C. L. Kuo, K. W. Lu, F. S. Yu, Y. S. Ma, J. L. Yang, Y. L. Chu, F. S. Chueh, K. C. Liu, and J. G. Chung. 2017. "Fisetin-induced apoptosis of human oral cancer SCC-4 cells through reactive oxygen species production, endoplasmic reticulum stress, caspase-, and mitochondria-dependent signaling pathways." *Environ Toxicol* 32 (6): 1725-1741. <https://doi.org/10.1002/tox.22396>.

Su, P., V. P. Veeraraghavan, S. Krishna Mohan, and W. Lu. 2019. "A ginger derivative, zingerone-a phenolic compound-induces ROS-mediated apoptosis in colon cancer cells (HCT-116)." *J Biochem Mol Toxicol* 33 (12): e22403. <https://doi.org/10.1002/jbt.22403>.

Sun, C., L. Zhao, X. Wang, Y. Hou, X. Guo, J. J. Lu, and X. Chen. 2022. "Psoralidin, a natural compound from Psoralea corylifolia, induces oxidative damage mediated apoptosis in colon cancer cells." *J Biochem Mol Toxicol* 36 (7): e23051. <https://doi.org/10.1002/jbt.23051>.

Sun, X., X. Zhong, W. Ma, W. Feng, Q. Huang, M. Ma, M. Lv, R. Hu, Z. Han, J. Li, and X. Zhou. 2022. "Germacrone induces caspase-3/GSDME activation and enhances ROS production, causing HepG2 pyroptosis." *Exp Ther Med* 24 (1): 456. <https://doi.org/10.3892/etm.2022.11383>.

Sun, Y., W. Guo, Y. Guo, Z. Lin, D. Wang, Q. Guo, and Y. Zhou. 2022. "Apoptosis induction in human prostate cancer cells related to the fatty acid metabolism by wogonin-mediated regulation of the AKT-SREBP1-FASN signaling network." *Food Chem Toxicol* 169: 113450. <https://doi.org/10.1016/j.fct.2022.113450>.

Sun, Y., Y. Qiao, Y. Liu, J. Zhou, X. Wang, H. Zheng, Z. Xu, J. Zhang, Y. Zhou, L. Qian, C. Zhang, and H. Lou. 2021. "ent-Kaurane diterpenoids induce apoptosis and ferroptosis through targeting redox resetting to overcome cisplatin resistance." *Redox Biol* 43: 101977. <https://doi.org/10.1016/j.redox.2021.101977>.

Sun, Z. L., J. L. Dong, and J. Wu. 2017. "Juglanin induces apoptosis and autophagy in human breast cancer progression via ROS/JNK promotion." *Biomed Pharmacother* 85: 303-312. <https://doi.org/10.1016/j.biopha.2016.11.030>.

Tang, S. Y., M. Z. Zhong, G. J. Yuan, S. P. Hou, L. L. Yin, H. Jiang, and ZΥ Yu. 2013. "Casticin, a flavonoid, potentiates TRAIL-induced apoptosis through modulation of anti-apoptotic proteins and death receptor 5 in colon cancer cells." *Oncol Rep* 29 (2): 474-80. <https://doi.org/10.3892/or.2012.2127>.

Tang, Z. Y., Y. Li, Y. T. Tang, X. D. Ma, and Z. Y. Tang. 2022. "Anticancer activity of oleanolic acid and its derivatives: Recent advances in evidence, target profiling and mechanisms of action." *Biomed Pharmacother* 145: 112397. <https://doi.org/10.1016/j.biopha.2021.112397>.

Termini, D., D. J. Den Hartogh, A. Jaglanian, and E. Tsiani. 2020. "Curcumin against Prostate Cancer: Current Evidence." *Biomolecules* 10 (11). <https://doi.org/10.3390/biom10111536>.

Totta, P., F. Acconcia, S. Leone, I. Cardillo, and M. Marino. 2004. "Mechanisms of naringenin-induced apoptotic cascade in cancer cells: involvement of estrogen receptor alpha and beta signalling." *IUBMB Life* 56 (8): 491-9. <https://doi.org/10.1080/15216540400010792>.

Tsai, C. F., W. L. Yeh, S. M. Huang, T. W. Tan, and D. Y. Lu. 2012. "Wogonin induces reactive oxygen species production and cell apoptosis in human glioma cancer cells." *Int J Mol Sci* 13 (8): 9877-9892. <https://doi.org/10.3390/ijms13089877>.

Tsai, M. F., S. M. Chen, A. Z. Ong, Y. H. Chung, P. N. Chen, Y. H. Hsieh, Y. T. Kang, and L. S. Hsu. 2021. "Shikonin Induced Program Cell Death through Generation of Reactive Oxygen Species in Renal Cancer Cells." *Antioxidants (Basel)* 10 (11). <https://doi.org/10.3390/antiox10111831>.

Vallejo, M. J., L. Salazar, and M. Grijalva. 2017. "Oxidative Stress Modulation and ROS-Mediated Toxicity in Cancer: A Review on In Vitro Models for Plant-Derived Compounds." *Oxid Med Cell Longev* 2017: 4586068. <https://doi.org/10.1155/2017/4586068>.

Vidhya, N., and S. N. Devaraj. 2011. "Induction of apoptosis by eugenol in human breast cancer cells." *Indian J Exp Biol* 49 (11): 871-8.

Wan, D., and H. Ouyang. 2018. "Baicalin induces apoptosis in human osteosarcoma cell through ROS-mediated mitochondrial pathway." *Nat Prod Res* 32 (16): 1996-2000. <https://doi.org/10.1080/14786419.2017.1359173>.

Wang, B., T. Y. Zhou, C. H. Nie, D. L. Wan, and S. S. Zheng. 2018. "Bigelovin, a sesquiterpene lactone, suppresses tumor growth through inducing apoptosis and autophagy via the inhibition of mTOR pathway regulated by ROS generation in liver cancer." *Biochem Biophys Res Commun* 499 (2): 156-163. <https://doi.org/10.1016/j.bbrc.2018.03.091>.

Wang, C., and C. Cui. 2019. "Inhibition of Lung Cancer Proliferation by Wogonin is Associated with Activation of Apoptosis and Generation of Reactive Oxygen Species." *Balkan Med J* 37 (1): 29-33. <https://doi.org/10.4274/balkanmedj.galenos.2019.2019.7.75>.

Wang, C. X., L. H. Chen, H. B. Zhuang, Z. S. Shi, Z. C. Chen, J. P. Pan, and Z. S. Hong. 2022. "Auriculasin enhances ROS generation to regulate colorectal cancer cell apoptosis, ferroptosis, oxeiptosis, invasion and colony formation." *Biochem Biophys Res Commun* 587: 99-106. <https://doi.org/10.1016/j.bbrc.2021.11.101>.

Wang, H., Z. Zhao, S. Lei, S. Li, Z. Xiang, X. Wang, X. Huang, G. Xia, and X. Huang. 2019. "Gambogic acid induces autophagy and combines synergistically with chloroquine to suppress pancreatic cancer by increasing the accumulation of reactive oxygen species." *Cancer Cell Int* 19: 7. <https://doi.org/10.1186/s12935-018-0705-x>.

Wang, J., A. M. Liao, K. Thakur, J. G. Zhang, J. H. Huang, and Z. J. Wei. 2019. "Licochalcone B Extracted from Glycyrrhiza uralensis Fisch Induces Apoptotic Effects in Human Hepatoma Cell HepG2." *J Agric Food Chem* 67 (12): 3341-3353. <https://doi.org/10.1021/acs.jafc.9b00324>.

Wang, J., Y. Yu, F. Hashimoto, Y. Sakata, M. Fujii, and D. X. Hou. 2004. "Baicalein induces apoptosis through ROS-mediated mitochondrial dysfunction pathway in HL-60 cells." *Int J Mol Med* 14 (4): 627-32.

Wang, J., Y. S. Zhang, K. Thakur, S. S. Hussain, J. G. Zhang, G. R. Xiao, and Z. J. Wei. 2018. "Licochalcone A from licorice root, an inhibitor of human hepatoma cell growth via induction of cell apoptosis and cell cycle arrest." *Food Chem Toxicol* 120: 407-417. <https://doi.org/10.1016/j.fct.2018.07.044>.

Wang, L., L. Liu, Y. Shi, H. Cao, R. Chaturvedi, M. W. Calcutt, T. Hu, X. Ren, K. T. Wilson, D. B. Polk, and F. Yan. 2012. "Berberine induces caspase-independent cell death in colon tumor cells through activation of apoptosis-inducing factor." *PLoS One* 7 (5): e36418. <https://doi.org/10.1371/journal.pone.0036418>.

Wang, Q. F., C. W. Chiang, C. C. Wu, C. C. Cheng, S. J. Hsieh, J. C. Chen, Y. C. Hsieh, and S. L. Hsu. 2007. "Gypenosides induce apoptosis in human hepatoma Huh-7 cells through a calcium/reactive oxygen species-dependent mitochondrial pathway." *Planta Med* 73 (6): 535-44. <https://doi.org/10.1055/s-2007-967200>.

Wang, Q., H. Wang, Y. Jia, H. Pan, and H. Ding. 2017. "Luteolin induces apoptosis by ROS/ER stress and mitochondrial dysfunction in gliomablastoma." *Cancer Chemother Pharmacol* 79 (5): 1031-1041. <https://doi.org/10.1007/s00280-017-3299-4>.

Wang, R., D. Deng, N. Shao, Y. Xu, L. Xue, Y. Peng, Y. Liu, and F. Zhi. 2018. "Evodiamine activates cellular apoptosis through suppressing PI3K/AKT and activating MAPK in glioma." *Onco Targets Ther* 11: 1183-1192. <https://doi.org/10.2147/ott.S155275>.

Wang, S., H. Li, S. Chen, Z. Wang, Y. Yao, T. Chen, Z. Ye, and P. Lin. 2020. "Andrographolide induces apoptosis in human osteosarcoma cells via the ROS/JNK pathway." *Int J Oncol* 56 (6): 1417-1428. <https://doi.org/10.3892/ijo.2020.5032>.

Wang, S., B. Lin, W. Liu, G. Wei, Z. Li, N. Yu, X. Xue, and G. Ji. 2020. "Acacetin Induces Apoptosis in Human Osteosarcoma Cells by Modulation of ROS/JNK Activation." *Drug Des Devel Ther* 14: 5077-5085. <https://doi.org/10.2147/dddt.S275148>.

Wang, X., H. Bai, X. Zhang, J. Liu, P. Cao, N. Liao, W. Zhang, Z. Wang, and C. Hai. 2013. "Inhibitory effect of oleanolic acid on hepatocellular carcinoma via ERK-p53-mediated cell cycle arrest and mitochondrial-dependent apoptosis." *Carcinogenesis* 34 (6): 1323-30. <https://doi.org/10.1093/carcin/bgt058>.

Wang, Z., X. Zhao, and X. Gong. 2016. "Costunolide induces lung adenocarcinoma cell line A549 cells apoptosis through ROS (reactive oxygen species)-mediated endoplasmic reticulum stress." *Cell Biol Int* 40 (3): 289-97. <https://doi.org/10.1002/cbin.10564>.

Watanabe, K., S. Kanno, A. Tomizawa, S. Yomogida, and M. Ishikawa. 2012. "Acacetin induces apoptosis in human T cell leukemia Jurkat cells via activation of a caspase cascade." *Oncol Rep* 27 (1): 204-9. <https://doi.org/10.3892/or.2011.1498>.

Waziri, P. M., R. Abdullah, S. K. Yeap, A. R. Omar, N. K. Kassim, I. Malami, C. W. How, I. C. Etti, and M. L. Abu. 2016. "Clausenidin induces caspase-dependent apoptosis in colon cancer." *BMC Complement Altern Med* 16: 256. <https://doi.org/10.1186/s12906-016-1247-1>.

Wei, J., M. Liu, H. Liu, H. Wang, F. Wang, Y. Zhang, L. Han, and X. Lin. 2013. "Oleanolic acid arrests cell cycle and induces apoptosis via ROS-mediated mitochondrial depolarization and lysosomal membrane permeabilization in human pancreatic cancer cells." *J Appl Toxicol* 33 (8): 756-65. <https://doi.org/10.1002/jat.2725>.

Wei, Zhao, Lao Yongxia, Liu Yang, Niu Jiqin, Xiao Zhihui, Arulselvan Palanisamy, and Shen Jian. 2022. "Escin induces apoptosis in ovarian cancer cell line by triggering S-phase cell cycle arrest and p38 MAPK/ERK pathway inhibition." *Journal of King Saud University - Science* 34 (1): 101644. <https://doi.org/https://doi.org/10.1016/j.jksus.2021.101644>. <https://www.sciencedirect.com/science/article/pii/S1018364721003062>.

Wen, J., K. R. You, S. Y. Lee, C. H. Song, and D. G. Kim. 2002. "Oxidative stress-mediated apoptosis. The anticancer effect of the sesquiterpene lactone parthenolide." *J Biol Chem* 277 (41): 38954-64. <https://doi.org/10.1074/jbc.M203842200>.

Wenzel, U., A. Nickel, S. Kuntz, and H. Daniel. 2004. "Ascorbic acid suppresses drug-induced apoptosis in human colon cancer cells by scavenging mitochondrial superoxide anions." *Carcinogenesis* 25 (5): 703-12. <https://doi.org/10.1093/carcin/bgh079>.

Won, Y. S., and K. I. Seo. 2020. "Lupiwighteone induces caspase-dependent and -independent apoptosis on human breast cancer cells via inhibiting PI3K/Akt/mTOR pathway." *Food Chem Toxicol* 135: 110863. <https://doi.org/10.1016/j.fct.2019.110863>.

Wu, B., W. Zeng, W. Ouyang, Q. Xu, J. Chen, B. Wang, and X. Zhang. 2020. "Quercetin induced NUPR1-dependent autophagic cell death by disturbing reactive oxygen species homeostasis in osteosarcoma cells." *J Clin Biochem Nutr* 67 (2): 137-145. <https://doi.org/10.3164/jcbn.19-121>.

Wu, C. Z., M. J. Gao, J. Chen, X. L. Sun, K. Y. Zhang, Y. Q. Dai, T. Ma, H. M. Li, and Y. X. Zhang. 2022. "Isobavachalcone Induces Multiple Cell Death in Human Triple-Negative Breast Cancer MDA-MB-231 Cells." *Molecules* 27 (20). <https://doi.org/10.3390/molecules27206787>.

Wu, D., J. Zhang, J. Wang, J. Li, F. Liao, and W. Dong. 2016. "Hesperetin induces apoptosis of esophageal cancer cells via mitochondrial pathway mediated by the increased intracellular reactive oxygen species." *Tumour Biol* 37 (3): 3451-9. <https://doi.org/10.1007/s13277-015-4176-6>.

Wu, F., R. Shao, P. Zheng, T. Zhang, C. Qiu, H. Sui, S. Li, L. Jin, H. Pan, X. Jin, P. Zou, R. Cui, and C. Xie. 2022. "Isoalantolactone Enhances the Antitumor Activity of Doxorubicin by Inducing Reactive Oxygen Species and DNA Damage." *Front Oncol* 12: 813854. <https://doi.org/10.3389/fonc.2022.813854>.

Wu, M., Y. Lao, N. Xu, X. Wang, H. Tan, W. Fu, Z. Lin, and H. Xu. 2015. "Guttiferone K induces autophagy and sensitizes cancer cells to nutrient stress-induced cell death." *Phytomedicine* 22 (10): 902-10. <https://doi.org/10.1016/j.phymed.2015.06.008>.

Wu, S., Y. Yang, F. Li, L. Huang, Z. Han, G. Wang, H. Yu, and H. Li. 2018. "Chelerythrine induced cell death through ROS-dependent ER stress in human prostate cancer cells." *Onco Targets Ther* 11: 2593-2601. <https://doi.org/10.2147/ott.S157707>.

Xie, J., M. H. Chen, C. P. Ying, and M. Y. Chen. 2020. "Neferine induces p38 MAPK/JNK1/2 activation to modulate melanoma proliferation, apoptosis, and oxidative stress." *Ann Transl Med* 8 (24): 1643. <https://doi.org/10.21037/atm-20-7201>.

Xu, C., X. Huang, X. Lei, Z. Jin, M. Wu, X. Liu, Y. Huang, X. Zhao, Y. Xiong, J. Sun, X. Duan, and J. Wang. 2021. "Costunolide-Induced Apoptosis via Promoting the Reactive Oxygen Species and Inhibiting AKT/GSK3β Pathway and Activating Autophagy in Gastric Cancer." *Front Cell Dev Biol* 9: 722734. <https://doi.org/10.3389/fcell.2021.722734>.

Xu, K. H., and D. P. Lu. 2010. "Plumbagin induces ROS-mediated apoptosis in human promyelocytic leukemia cells in vivo." *Leuk Res* 34 (5): 658-65. <https://doi.org/10.1016/j.leukres.2009.08.017>.

Xu, Y., Y. Tong, J. Ying, Z. Lei, L. Wan, X. Zhu, F. Ye, P. Mao, X. Wu, R. Pan, B. Peng, Y. Liu, and J. Zhu. 2018. "Chrysin induces cell growth arrest, apoptosis, and ER stress and inhibits the activation of STAT3 through the generation of ROS in bladder cancer cells." *Oncol Lett* 15 (6): 9117-9125. <https://doi.org/10.3892/ol.2018.8522>.

Yaffe, P. B., M. R. Power Coombs, C. D. Doucette, M. Walsh, and D. W. Hoskin. 2015. "Piperine, an alkaloid from black pepper, inhibits growth of human colon cancer cells via G1 arrest and apoptosis triggered by endoplasmic reticulum stress." *Mol Carcinog* 54 (10): 1070-85. <https://doi.org/10.1002/mc.22176>.

Yan, H., X. Wang, J. Niu, Y. Wang, P. Wang, and Q. Liu. 2014. "Anti-cancer effect and the underlying mechanisms of gypenosides on human colorectal cancer SW-480 cells." *PLoS One* 9 (4): e95609. <https://doi.org/10.1371/journal.pone.0095609>.

Yan, H., X. Wang, Y. Wang, P. Wang, and Y. Xiao. 2014. "Antiproliferation and anti-migration induced by gypenosides in human colon cancer SW620 and esophageal cancer Eca-109 cells." *Hum Exp Toxicol* 33 (5): 522-33. <https://doi.org/10.1177/0960327113497771>.

Yan, Y. Y., J. P. Bai, Y. Xie, J. Z. Yu, and C. G. Ma. 2013. "The triterpenoid pristimerin induces U87 glioma cell apoptosis through reactive oxygen species-mediated mitochondrial dysfunction." *Oncol Lett* 5 (1): 242-248. <https://doi.org/10.3892/ol.2012.982>.

Yang, C., J. Song, S. Hwang, J. Choi, G. Song, and W. Lim. 2021. "Apigenin enhances apoptosis induction by 5-fluorouracil through regulation of thymidylate synthase in colorectal cancer cells." *Redox Biol* 47: 102144. <https://doi.org/10.1016/j.redox.2021.102144>.

Yang, J. T., Z. L. Li, J. Y. Wu, F. J. Lu, and C. H. Chen. 2014. "An oxidative stress mechanism of shikonin in human glioma cells." *PLoS One* 9 (4): e94180. <https://doi.org/10.1371/journal.pone.0094180>.

Yang, K. M., J. O. Pyo, G. Y. Kim, R. Yu, I. S. Han, S. A. Ju, W. H. Kim, and B. S. Kim. 2009. "Capsaicin induces apoptosis by generating reactive oxygen species and disrupting mitochondrial transmembrane potential in human colon cancer cell lines." *Cell Mol Biol Lett* 14 (3): 497-510. <https://doi.org/10.2478/s11658-009-0016-2>.

Yang, M., W. Zhang, X. Yu, F. Wang, Y. Li, Y. Zhang, and Y. Yang. 2021. "Helenalin Facilitates Reactive Oxygen Species-Mediated Apoptosis and Cell Cycle Arrest by Targeting Thioredoxin Reductase-1 in Human Prostate Cancer Cells." *Med Sci Monit* 27: e930083. <https://doi.org/10.12659/msm.930083>.

Yang, Y. I., J. H. Kim, K. T. Lee, and J. H. Choi. 2011. "Costunolide induces apoptosis in platinum-resistant human ovarian cancer cells by generating reactive oxygen species." *Gynecol Oncol* 123 (3): 588-96. <https://doi.org/10.1016/j.ygyno.2011.08.031>.

Yao, Z., X. Xu, and Y. Huang. 2021. "Daidzin inhibits growth and induces apoptosis through the JAK2/STAT3 in human cervical cancer HeLa cells." *Saudi J Biol Sci* 28 (12): 7077-7081. <https://doi.org/10.1016/j.sjbs.2021.08.011>.

Ye, F., H. Wang, L. Zhang, Y. Zou, H. Han, and J. Huang. 2015. "Baicalein induces human osteosarcoma cell line MG-63 apoptosis via ROS-induced BNIP3 expression." *Tumour Biol* 36 (6): 4731-40. <https://doi.org/10.1007/s13277-015-3122-y>.

Yin, H. Q., Y. H. Kim, C. K. Moon, and B. H. Lee. 2005. "Reactive oxygen species-mediated induction of apoptosis by a plant alkaloid 6-methoxydihydrosanguinarine in HepG2 cells." *Biochem Pharmacol* 70 (2): 242-8. <https://doi.org/10.1016/j.bcp.2005.04.020>.

Yoo, C. B., K. T. Han, K. S. Cho, J. Ha, H. J. Park, J. H. Nam, U. H. Kil, and K. T. Lee. 2005. "Eugenol isolated from the essential oil of Eugenia caryophyllata induces a reactive oxygen species-mediated apoptosis in HL-60 human promyelocytic leukemia cells." *Cancer Lett* 225 (1): 41-52. <https://doi.org/10.1016/j.canlet.2004.11.018>.

Yousef, B. A., H. M. Hassan, M. Guerram, A. M. Hamdi, B. Wang, L. Y. Zhang, and Z. Z. Jiang. 2016. "Pristimerin inhibits proliferation, migration and invasion, and induces apoptosis in HCT-116 colorectal cancer cells." *Biomed Pharmacother* 79: 112-9. <https://doi.org/10.1016/j.biopha.2016.02.003>.

Yu, J. S., and A. K. Kim. 2011. "Wogonin induces apoptosis by activation of ERK and p38 MAPKs signaling pathways and generation of reactive oxygen species in human breast cancer cells." *Mol Cells* 31 (4): 327-35. <https://doi.org/10.1007/s10059-011-0041-7>.

Yuan, S. Y., C. L. Cheng, S. S. Wang, H. C. Ho, K. Y. Chiu, C. S. Chen, C. C. Chen, M. Y. Shiau, and Y. C. Ou. 2017. "Escin induces apoptosis in human renal cancer cells through G2/M arrest and reactive oxygen species-modulated mitochondrial pathways." *Oncol Rep* 37 (2): 1002-1010. <https://doi.org/10.3892/or.2017.5348>.

Yue, Z., X. Xiao, J. Wu, X. Zhou, W. Liu, Y. Liu, H. Li, G. Chen, Y. Wu, and X. Lei. 2018. "ent-Jungermannenone C Triggers Reactive Oxygen Species-Dependent Cell Differentiation in Leukemia Cells." *J Nat Prod* 81 (2): 298-306. <https://doi.org/10.1021/acs.jnatprod.7b00722>.

Yun, D., S. Y. Yoon, S. J. Park, and Y. J. Park. 2021. "The Anticancer Effect of Natural Plant Alkaloid Isoquinolines." *Int J Mol Sci* 22 (4). <https://doi.org/10.3390/ijms22041653>.

Zhang, J., N. Wang, Y. Zhou, K. Wang, Y. Sun, H. Yan, W. Han, X. Wang, B. Wei, Y. Ke, and X. Xu. 2021. "Oridonin induces ferroptosis by inhibiting gamma-glutamyl cycle in TE1 cells." *Phytother Res* 35 (1): 494-503. <https://doi.org/10.1002/ptr.6829>.

Zhang, J., D. Wu, Vikash, J. Song, J. Wang, J. Yi, and W. Dong. 2015. "Hesperetin Induces the Apoptosis of Gastric Cancer Cells via Activating Mitochondrial Pathway by Increasing Reactive Oxygen Species." *Dig Dis Sci* 60 (10): 2985-95. <https://doi.org/10.1007/s10620-015-3696-7>.

Zhang, Q., J. Bao, and J. Yang. 2019. "Genistein-triggered anticancer activity against liver cancer cell line HepG2 involves ROS generation, mitochondrial apoptosis, G2/M cell cycle arrest and inhibition of cell migration." *Arch Med Sci* 15 (4): 1001-1009. <https://doi.org/10.5114/aoms.2018.78742>.

Zhang, Q., W. Chen, X. Lv, Q. Weng, M. Chen, R. Cui, G. Liang, and J. Ji. 2022. "Corrigendum: Piperlongumine, a Novel TrxR1 Inhibitor, Induces Apoptosis in Hepatocellular Carcinoma Cells by ROS-Mediated ER Stress." *Front Pharmacol* 13: 806724. <https://doi.org/10.3389/fphar.2022.806724>.

Zhang, R., J. Chen, L. Mao, Y. Guo, Y. Hao, Y. Deng, X. Han, Q. Li, W. Liao, and M. Yuan. 2020. "Nobiletin Triggers Reactive Oxygen Species-Mediated Pyroptosis through Regulating Autophagy in Ovarian Cancer Cells." *J Agric Food Chem* 68 (5): 1326-1336. <https://doi.org/10.1021/acs.jafc.9b07908>.

Zhang, T., Y. Li, K. A. Park, H. S. Byun, M. Won, J. Jeon, Y. Lee, J. H. Seok, S. W. Choi, S. H. Lee, J. Man Kim, J. H. Lee, C. G. Son, Z. W. Lee, H. M. Shen, and G. M. Hur. 2012. "Cucurbitacin induces autophagy through mitochondrial ROS production which counteracts to limit caspase-dependent apoptosis." *Autophagy* 8 (4): 559-76. <https://doi.org/10.4161/auto.18867>.

Zhang, X., and H. M. Zhang. 2019. "Alantolactone induces gastric cancer BGC-823 cell apoptosis by regulating reactive oxygen species generation and the AKT signaling pathway." *Oncol Lett* 17 (6): 4795-4802. <https://doi.org/10.3892/ol.2019.10172>.

Zhang, Y., M. Gao, M. Zhu, H. Li, T. Ma, and C. Wu. 2022. "[Isobavachalcone induces cell death through multiple pathways in human breast cancer MCF-7 cells]." *Nan Fang Yi Ke Da Xue Xue Bao* 42 (6): 878-885. <https://doi.org/10.12122/j.issn.1673-4254.2022.06.11>.

Zhang, Y., J. Soboloff, Z. Zhu, and S. A. Berger. 2006. "Inhibition of Ca2+ influx is required for mitochondrial reactive oxygen species-induced endoplasmic reticulum Ca2+ depletion and cell death in leukemia cells." *Mol Pharmacol* 70 (4): 1424-34. <https://doi.org/10.1124/mol.106.024323>.

Zhang, Z., Y. Pan, Y. Zhao, M. Ren, Y. Li, G. Lu, K. Wu, and S. He. 2021. "Delphinidin modulates JAK/STAT3 and MAPKinase signaling to induce apoptosis in HCT116 cells." *Environ Toxicol* 36 (8): 1557-1566. <https://doi.org/10.1002/tox.23152>.

Zhao, B., and X. Li. 2014. "Altholactone induces reactive oxygen species-mediated apoptosis in bladder cancer T24 cells through mitochondrial dysfunction, MAPK-p38 activation and Akt suppression." *Oncol Rep* 31 (6): 2769-75. <https://doi.org/10.3892/or.2014.3126>.

Zhao, Q., Y. Liu, J. Zhong, Y. Bi, Y. Liu, Z. Ren, X. Li, J. Jia, M. Yu, and X. Yu. 2019. "Pristimerin induces apoptosis and autophagy via activation of ROS/ASK1/JNK pathway in human breast cancer in vitro and in vivo." *Cell Death Discov* 5: 125. <https://doi.org/10.1038/s41420-019-0208-0>.

Zhao, Y., S. Roy, C. Wang, and A. Goel. 2022. "A Combined Treatment with Berberine and Andrographis Exhibits Enhanced Anti-Cancer Activity through Suppression of DNA Replication in Colorectal Cancer." *Pharmaceuticals (Basel)* 15 (3). <https://doi.org/10.3390/ph15030262>.

Zhao, Z., Y. Wang, Y. Gong, X. Wang, L. Zhang, H. Zhao, J. Li, J. Zhu, X. Huang, C. Zhao, L. Yang, and L. Wang. 2022. "Celastrol elicits antitumor effects by inhibiting the STAT3 pathway through ROS accumulation in non-small cell lung cancer." *J Transl Med* 20 (1): 525. <https://doi.org/10.1186/s12967-022-03741-9>.

Zhen, Y. Z., Y. J. Lin, K. J. Li, X. S. Yang, Y. F. Zhao, J. Wei, J. B. Wei, and G. Hu. 2015. "Gambogic Acid lysinate induces apoptosis in breast cancer mcf-7 cells by increasing reactive oxygen species." *Evid Based Complement Alternat Med* 2015: 842091. <https://doi.org/10.1155/2015/842091>.

Zhong, F., Z. T. Tong, L. L. Fan, L. X. Zha, F. Wang, M. Q. Yao, K. S. Gu, and Y. X. Cao. 2016. "Guggulsterone-induced apoptosis in cholangiocarcinoma cells through ROS/JNK signaling pathway." *Am J Cancer Res* 6 (2): 226-37.

Zhou, G., Z. Yang, X. Wang, R. Tao, and Y. Zhou. 2017. "TRAIL Enhances Shikonin Induced Apoptosis through ROS/JNK Signaling in Cholangiocarcinoma Cells." *Cell Physiol Biochem* 42 (3): 1073-1086. <https://doi.org/10.1159/000478758>.

Zhou, X. Y., X. Y. Liu, Z. Li, and X. H. Guo. 2022. "[Research progress on antitumor effect and molecular mechanism of capsaicin]." *Zhongguo Zhong Yao Za Zhi* 47 (16): 4277-4283. <https://doi.org/10.19540/j.cnki.cjcmm.20220421.601>.

Zhou, Y., Y. Peng, Q. Q. Mao, X. Li, M. W. Chen, J. Su, L. Tian, N. Q. Mao, L. Z. Long, M. F. Quan, F. Liu, S. F. Zhou, and Y. X. Zhao. 2013. "Casticin induces caspase-mediated apoptosis via activation of mitochondrial pathway and upregulation of DR5 in human lung cancer cells." *Asian Pac J Trop Med* 6 (5): 372-8. <https://doi.org/10.1016/s1995-7645(13)60041-3>.

Zhou, Y., L. Tian, L. Long, M. Quan, F. Liu, and J. Cao. 2013. "Casticin potentiates TRAIL-induced apoptosis of gastric cancer cells through endoplasmic reticulum stress." *PLoS One* 8 (3): e58855. <https://doi.org/10.1371/journal.pone.0058855>.

Zhu, J., W. Yu, B. Liu, Y. Wang, J. Shao, J. Wang, K. Xia, C. Liang, W. Fang, C. Zhou, and H. Tao. 2017. "Escin induces caspase-dependent apoptosis and autophagy through the ROS/p38 MAPK signalling pathway in human osteosarcoma cells in vitro and in vivo." *Cell Death Dis* 8 (10): e3113. <https://doi.org/10.1038/cddis.2017.488>.

Zhu, M. L., P. M. Zhang, M. Jiang, S. W. Yu, and L. Wang. 2020. "Myricetin induces apoptosis and autophagy by inhibiting PI3K/Akt/mTOR signalling in human colon cancer cells." *BMC Complement Med Ther* 20 (1): 209. <https://doi.org/10.1186/s12906-020-02965-w>.

Zhu, W. B., F. J. Tian, and L. Q. Liu. 2017. "Chikusetsu (CHI) triggers mitochondria-regulated apoptosis in human prostate cancer via reactive oxygen species (ROS) production." *Biomed Pharmacother* 90: 446-454. <https://doi.org/10.1016/j.biopha.2017.03.050>.

Zhuge, W., R. Chen, K. Vladimir, X. Dong, K. Zia, X. Sun, X. Dai, M. Bao, X. Shen, and G. Liang. 2018. "Costunolide specifically binds and inhibits thioredoxin reductase 1 to induce apoptosis in colon cancer." *Cancer Lett* 412: 46-58. <https://doi.org/10.1016/j.canlet.2017.10.006>.

Zou, J., Y. Zhang, J. Sun, X. Wang, H. Tu, S. Geng, R. Liu, Y. Chen, and Z. Bi. 2017. "Deoxyelephantopin Induces Reactive Oxygen Species-Mediated Apoptosis and Autophagy in Human Osteosarcoma Cells." *Cell Physiol Biochem* 42 (5): 1812-1821. <https://doi.org/10.1159/000479537>.

Zunino, S. J., J. M. Ducore, and D. H. Storms. 2007. "Parthenolide induces significant apoptosis and production of reactive oxygen species in high-risk pre-B leukemia cells." *Cancer Lett* 254 (1): 119-27. <https://doi.org/10.1016/j.canlet.2007.03.002>.
